# Neural correlates of peripartum depression: a systematic review, meta-analysis and comparison to major depressive disorder

**DOI:** 10.1038/s41380-025-03227-2

**Published:** 2025-09-08

**Authors:** Mónica Sobral, Raquel Guiomar, Manya Rezaeian, Maria Vasileiadi, Sara Cruz, Francisca Pacheco, Vera Mateus, Roser Palau-Costafreda, Johanna Pozo-Neira, Ana Weidenauer, Helena Moreira, Martin Tik, Ana Ganho-Ávila, Anna-Lisa Schuler

**Affiliations:** 1 Faculty of Psychology and Educational Sciences, University of Coimbra, Rua do Colégio Novo, Coimbra, 3000-315 Portugal; 2 Center for Research in Neuropsychology and Cognitive Behavioral Intervention, Faculty of Psychology and Educational Sciences, University of Coimbra, Rua do Colégio Novo, 3000-315 Coimbra, Portugal; 3 Human Developmental Sciences Graduate Program and Mackenzie Center for Research in Childhood and Adolescence, Center for Biological and Health Sciences, Mackenzie Presbyterian University, São Paulo, Brazil; 4 Counseling Center of Tehran University, Tehran, Iran; 5 Medical University of Vienna, Vienna, Austria; 6 Department of Psychology, School of Philosophy, Psychology & Language Sciences, University of Edinburgh, Edinburgh, UK; 7 The Psychology for Development Research Centre, Lusiada University, Porto, Portugal; 8 Social Determinants and Health Education Research Group (SDHEd), Hospital del Mar Research Institute. Hospital del Mar Nursing School (ESIHMar), Universitat Pompeu Fabra-affiliated, Barcelona, Spain; 9 PsyBrain Research Group, Institute of Neuroscience, Clinical Psychology School, Health and Wellness Academic Unit, Universidad Católica de Cuenca, Cuenca, Ecuador; 10 Stanford University Department of Psychiatry and Behavioral Sciences, Palo Alto, CA USA; 11 Research Group Cognition and Plasticity, Max Planck Institute for Human Cognitive and Brain Sciences, Leipzig, Germany

**Keywords:** Depression, Neuroscience

## Abstract

**Background:**

Peripartum depression (PPD) is a form of major depressive disorder (MDD) that begins during the peripartum period and poses a significant mental health challenge affecting 10 to 29% of women.

**Objective:**

This systematic review and multimodal activation likelihood estimation (ALE) meta-analysis explored the distinct structural, functional, and metabolic features of the PPD brain as compared to female non-peripartum MDD.

**Methods:**

For this purpose, we conducted a comprehensive literature search in PubMed, Embase and PsycINFO databases to identify peer-reviewed original studies investigating the neural correlates associated with PPD or fMDD.

**Results:**

Forty-five studies in PPD and 55 in fMDD were included in the qualitative synthesis. From these, 25 PPD and 32 fMDD studies were included in the meta-analysis. Both shared and distinct neural underpinnings of PPD and fMDD were observed. Specifically, we found alterations in the cognitive control, salience and default mode networks for both PPD and fMDD, although with reversed structural and functional activity patterns in the insula, amygdala, precentral gyrus and precuneus.

**Conclusions:**

These findings support the consistent pattern of dysregulation associated with emotional regulation, cognition and maternal caregiving in women with PPD, as well as possible differential sensitivity to hormonal influences, highlighting the need for targeted interventions.

## Introduction

Peripartum depression (PPD) is a major depressive disorder (MDD) with onset during pregnancy or after childbirth [1, 2]. It is characterised by sadness, restlessness/agitation, impaired concentration, and sleep/appetite disturbances [3]. Multiple systematic reviews and meta-analyses yielded an estimated prevalence of PPD ranging between 10 to 29% [2, 4–7], constituting a serious mental health issue with well-established detrimental effects on the mother’s well-being and infant’s emotional, behavioural and cognitive development [8]. Emerging literature has demonstrated that PPD renders altered brain structure and functional connectivity in peripartum women [9, 10]. However, the brain response patterns appear to differ from those reported in similar symptom profiles outside the peripartum period, such as MDD [9, 11].

The peripartum period encompasses profound environmental/social, psychological, and hormonal changes that impact brain plasticity, influencing maternal behavior and caregiving toward infants [12]. Neurobiological adaptations are observed in brain regions associated with emotion processing (e.g., prefrontal cortex [PFC]), salience/threat detection (e.g., dorsal anterior cingulate cortex [ACC], anterior insula), reward/motivation (e.g., striatum, medial PFC, thalamus) and social cognition (e.g., posterior cingulate [PCC], temporoparietal regions; [11, 13]). These adaptations can enhance maternal responsiveness and bonding by facilitating the acquisition of experience-dependent skills and knowledge related to motherhood tasks (e.g., threat vigilance, inferring what the infants’ feelings and needs are; [11, 12, 14]).

Maternal brain plasticity, alongside hormonal fluctuations and external stressors, may also increase vulnerability to peripartum mental disorders, including PPD [11]. Structural, functional, and molecular studies in PPD have consistently revealed changes in brain areas associated with both depression and maternal caregiving, such as the hypothalamus, amygdala (AMY), ACC, orbitofrontal (OFC) and dorsolateral prefrontal cortices (DLPFC), insula and striatum [9, 11, 15]. The abnormal correlates in these regions may be indicative of the neural mechanisms of PPD and consequent impaired caregiving abilities [16]. However, the literature is hindered by several limitations, including small sample sizes and underpowered studies (generally involving only 4 to 30 PPD women). This poses a significant challenge when attempting to interpret and synthesise the existing PPD imaging literature.

Diagnostically, PPD is often considered a subtype of MDD (with specifiers for peripartum onset in the Diagnostic and Statistical Manual of Mental Disorders, 5th Edition, Text Revision [DSM-5-TR] and the International Classification of Disorders, 11th Edition [ICD-11]), but other evidence suggests that PPD has distinct clinical characteristics compared to non-peripartum MDD. For instance, PPD is associated with more common and/or severe symptoms of anxiety, irritability, psychomotor restlessness and agitation, obsessive thoughts, fatigue, loss of energy and impaired concentration and decision-making, as well as specific guilt related to motherhood but less sad mood and suicidal ideation [17, 18]. However, evidence for differentiating PPD from MDD is inconsistent, partly due to differences in the definition of the postpartum period. Studies focusing on depression in the early postpartum period suggest that PPD may be characterized by unique features related to symptom severity, heritability, and epigenetic factors and may stem from biological factors (e.g., [19]). In contrast, depression occurring in the later postpartum period may resemble MDD observed outside the peripartum period and be more influenced by psychosocial factors [20].

Reviews comparing brain response patterns in PPD and non-peripartum MDD have also revealed notable differences [3, 9, 11], with reversed activation patterns in the AMY and PFC [16]. For example, women diagnosed with PPD typically show a blunted AMY response to non-infant-related negative stimuli (e.g., [21]), whereas MDD patients have a heightened AMY response [22]. Understanding these distinct neurobiological profiles is essential for developing appropriate treatment approaches, as depression related to the female reproductive cycle (such as PPD) may represent a distinct biotype [11, 19].

Several reviews have explored the neurobiological underpinnings of PPD (e.g., [15, 23, 24]), but a comprehensive meta-analysis or direct comparison with female-only non-peripartum unipolar depression (fMDD) remains lacking in the literature. To address this gap, our study aims to extend previous reviews of the neural correlates of PPD relative to the healthy postpartum brain and to conduct a formal comparison with fMDD in relation to the healthy female brain. Considering symptom presentations observed in PPD (e.g., obsessive thoughts, increased anxiety and impaired concentration and decision-making; [17]), our focus is on the cognitive control network (CCN), particularly the ACC-DLPFC axis, due to its involvement in emotional and social regulation, its interaction with attention and default mode networks (DMN) and impact on treatment outcomes [25–27].

## Methods

### Registration and protocol

The review followed the Preferred Reporting Items for Systematic Reviews and Meta-Analysis (PRISMA) guidelines ([28]; checklist in supplementary Table S1). The study protocol was preregistered on the International Prospective Register of Systematic Reviews (PROSPERO; CRD42021281870).

### Eligibility criteria

We included English peer-reviewed original studies in which: (1) participants presented PPD (search 1) or MDD (search 2) and no other clinical diagnoses, except for anxiety symptoms/diagnosis; (2) all participants were aged between 18–60 years old; (3) for PPD, participants were assessed from pregnancy up to 1 year postpartum (current pregnancy/postpartum); (4) structural, functional or metabolic differences were assessed using structural or functional magnetic resonance imaging (MRI), diffusion tensor/kurtosis imaging (DTI/DKI), computed tomography (CT), positron emission tomography (PET), near-infrared spectroscopy (NIRS) or magnetic resonance spectroscopy (MRS); (5) a within- or between-group comparison or correlation with symptom severity was reported. For the quantitative analysis, peak coordinates of significant contrast of interest findings were reported in Montreal Neurological Institute (MNI) or Talairach standard spaces. Studies with imaging modalities lacking coordinate information, such as NIRS and MRS, were included in the qualitative synthesis of results. Based on the authors’ expertise an additional search was performed for Portuguese, Spanish, German, French, Dutch, and Greek articles in the PubMed database. This search did not result in any additional hits.

Additional exclusion criteria were: (1) nonempirical studies (e.g., review, meta-analysis); (2) not published in English language or any language native to the authors; (3) depressive disorder diagnosed prior to pregnancy (past history only or euthymic patients excluded); (4) patients were subject to an intervention, unless the study reported a baseline comparison with healthy controls (HC); (5) electroencephalogram (EEG) or magnetoencephalogram (MEG) studies.

### Information sources and search strategy

A systematic literature search was performed in the PubMed, PsycINFO (through Ovid), and Embase electronic databases, from inception until 24 February 2021. Updated searches were conducted on 27 July 2024. Search terms related to different imaging modalities including “magnetic resonance imaging”, “magnetic resonance spectroscopy”, “diffusion tensor imaging”, “computed tomography”, “near infrared spectrometry” or “positron emission tomography” and terms denoting “pregnancy” and “birth” and “depression” were included. For the search strategy on fMDD-related correlates, terms denoting “pregnancy” and “birth” were removed. The complete search strategy for all databases is reported in supplementary Table S2. Manual searches of reference lists from relevant reviews and included studies were also conducted to identify any additional studies that met the eligibility criteria.

### Study selection and data extraction

In search 1, the title and abstract screening, as well as the full-text review and data extraction of retrieved reports, were conducted independently by two researchers (MR [search 1])/MS [updated search] and RG). For Search 2, due to the high number of results, seven reviewers (MS, RG, MR, MV, FP, RP-C, JP-N) were involved in screening the abstracts and full-texts and data extraction of the reports, with one designated reviewer (MS) cross-verifying all the decisions made by the team to ensure consistency and accuracy. The screening was performed using the Rayyan software [29]. Any conflicts or discrepancies during this process were resolved through discussions until a consensus was reached.

The extracted data consisted of demographic and clinical data (number of participants, age, age range, diagnostic criteria and/or assessment instrument and cut-off scores, comorbidity, symptom severity, treatment status, parity [only in search 1], peripartum timepoint [only in search 1] and pregnancy history [only in search 2]), methodological details (study design, imaging technique, task/measure) and main findings (direction of effect, qualitative and peak coordinates of significant correlates).

### Risk of bias assessment

We assessed the risk of bias in the included studies in PPD and fMDD using the Newcastle-Ottawa Scale (NOS; [30]) for cohort studies and the Joanna Briggs Institute (JBI) Critical Appraisal Checklist for Analytical Cross-Sectional Studies [31] for cross-sectional studies. The assessment was conducted independently by two reviewers (MS, RG). Disagreements were resolved by consensus. Following the literature, we derived an overall summary risk of bias judgement in NOS (low quality [0–2 items], moderate quality [3–5 items] and good/high quality [6–7 items]) and JBI (low quality [0–3 items]; moderate quality [4–5 items]; high quality [6–8 items]). While the overall appraisal results serve as an additional source of information regarding the quality of the studies, they were not used to exclude any reports.

### Synthesis methods and statistical analysis

Reports were grouped based on the imaging modality (structural, task-based and resting-state functional and molecular imaging) and synthesised using a tabular and narrative format. For the comparison analysis, we selected a subgroup of MDD studies (*n* = 55) focused exclusively on female participants to minimize sex effects. We also contacted the authors of these studies to request additional information about participants’ previous and current history of pregnancy (a possible confounding factor).

A coordinate-based ALE meta-analysis was conducted to combine peak coordinates from included studies, using GingerALE 3.0.2 (https://brainmap.org/; [32, 33]). Prior to analysis, all activation foci coordinates reported in Talairach space (*n* = 10) were transformed into MNI space using the Lancaster transform, implemented in the GingerALE toolbox (icbm2tal; [32]). To avoid repeated inclusion of the same sample and spurious findings [34], we carefully examined the included studies for overlap (team members, location, recruitment interval, sample size and age) and aggregated data when it coincided (*n* = 3; [35–37]). We used GingerALE’s less conservative gray matter mask and subject-based Full-Width Half-Max (FWHM) values [33].

To address our research question, we assessed imaging method-specific differences (PPD / fMDD vs HC as measured by structural MRI/DTI, resting-state and task-based fMRI). For the PPD / fMDD multimodal analyses, all coordinates from multiple experiments (i.e., different imaging modalities or tasks and contrasts) of the same study were merged, guaranteeing that each sample was only represented by one experiment. We then performed conjunction and contrast analyses between PPD vs fMDD. Exploratory analyses were conducted to contrast PDD and fMDD with HC considering direction of effect (PPD / fMDD brain structure / function greater than HC and PPD / fMDD less than HC). To assess the impact of age, we conducted an exploratory analysis by considering only a subgroup of studies with age-matched fMDD participants.

According to best practices [38], for the imaging-specific and multimodal analyses, results were thresholded for significance using cluster-level inference of p < 0.05 with a cluster-forming threshold of p < 0.001 with 10000 thresholding permutations. For contrast analyses (PPD vs. fMDD) significance level was set to a p-value of below 0.001 shuffling through 10000 permutations. For the exploratory direction of effect, conjunction and age-matched analyses, we adopted a less conservative statistical threshold of uncorrected p < 0.05. All resulting coordinate clusters are reported in MNI space and overlaid on an MNI-normalised template using MRIcroGL (http://www.mccauslandcenter.sc.edu/mricrogl/) or Surf Ice (https://www.nitrc.org/projects/surfice/).

Additionally, we submitted the MNI coordinates of peak values to Neurosynth (http://www.neurosynth.org) to explore functional networks in PPD and fMDD, through seed-based connectivity analysis. Moreover, in order to evaluate the overlap between common depression symptom networks and PPD and fMDD, we calculated dice indices between the dysphoric and anxiosomatic networks according to Sidiqqi et al. [39].

In order to account for specificity of our PPD ALE results we created a dummy dataset (*n* = 25) consisting of comparable numbers of participants and MNI-coordinates to the PPD dataset. The dummy data can be found in the Supplementary Materials (S9).

## Results

### Study selection

Our database search yielded 1048 records for search 1 (PPD) and 13338 for search 2 (MDD). After removing duplicates, we carefully screened 704 and 9399 records and conducted a thorough review of 60 and 916 full-text documents, respectively. We included 45 articles that met our inclusion criteria for PPD [21, 35–37, 40–80] and 55 for fMDD ([55, 81–134]; a flow chart is available in supplementary Fig. S1). For a multimodal meta-analysis of both female and male MDD participants (literature review until 2021), please refer to supplementary Table S3 and Fig. S2.

### Study characteristics and risk of bias

Demographic, methodological and outcome characteristics of the included studies in PPD and fMDD are summarised in Tables 1 and 2 (a comparison of PPD and fMDD studies main characteristics is available in supplementary Table S4 and Figs. S3–S5). PPD reports include data on DTI/DKI (*n* = 3), structural MRI (*n* = 7), resting-state (*n* = 21), and task-based fMRI (*n* = 8), fNIRS (*n* = 2), MRS (n = 5) and PET (*n* = 2). PPD was diagnosed according to standardized diagnostic criteria (e.g., DSM; major depressive episode with peripartum onset) in most studies, except for four studies where cut-off scores from validated self-report questionnaires were used [21, 68, 74, 76]. The majority of reports (96%) focused exclusively on the postpartum period, ranging from early [21, 35–37, 41–45, 49, 50, 52, 57–59, 61, 64–67, 69–72, 77, 80] to late postpartum [62, 68, 73, 75, 76, 78] and unspecified (up to 1 year postpartum; [40, 46–48, 51, 55, 56, 60, 63, 79]), with only two studies collecting data antenatally (2nd or 3rd trimester; [53, 54]).

**Table 1:** Summary of study characteristics and results of included Peripartum Depression studies.

Reference	Design	Paradigm/Measure	Group	Age (M, SD, range)	Parity	Timepoint	Diagnostic criteria	Severity (M, SD)	Comorbidity	Treatment status	Direction of effect	Main finding	Study quality	Included in ALE (yes/no)
*Diffusion tensor (DTI) or kurtosis imaging (DKI)*														
[40] Long et al.	Cross-sectional	FA, AD and RD	PPD (n = 51)	31.45 (3.63) 21–42	1 (0.34)	within 1 year pp	DSM-5 and CCMD-3 First episode	EPDS: 17.49 (3.94)	None	Treatment-naive	PPD > HC	↑ increased FA and AD in right anterior thalamic radiation tract and ↑ FA in cingulum tract	High	No
			HC (n = 49)	31.86 (4.04) 21–42	1.27 (0.44)						PPD < HC	↓ RD in cingulum tract		
[41] Sasaki et al.	Prospective cohort	MK, FA and MD	PPD (n = 8)	35.6 (6.3) 24–44	75% primiparous	1–2 months pp	DSM-5	Mild	None History of mental disorder excluded	NA	PPD > HC	↑ MD in widespread white matter (temporo-parietal regions, superior longitudinal fasciculus, corticospinal tract, cingulum, body/splenium of corpus callosum, external capsule, anterior/posterior limb of internal capsule, inferior longitudinal fasciculus and putamen)	High	No
			HC (n = 67)	35.1 (4.5) 20-NA	64.2% primiparous						PPD < HC	↓ FA in superior longitudinal fasciculus and corticospinal tract and thalamus		
[42] Silver et al.	Prospective cohort	FA	PPD (n = 16)	28.97 (4.91) 19–38	31.3% primiparous	2–8 weeks pp	DSM-IV	EPDS: 14.75 (3.45)	Anxiety disorders (87.50%) past MDD (62.5%) past PPD (25%)	Medication-free	PPD < HC	↓ FA in left anterior limb of internal capsule	High	Yes
			HC (n = 22)	28.15 (5.35) 19–38	40.9% primiparous									
*Structural magnetic resonance imaging (MRI)*														
[43] Chen et al.	Cross-sectional	GMV	PPD (n = 28)	29.75 (4.49) ≥20	85.7% primiparous	6 weeks pp	DSM-IV and CCMD-3 First episode	EPDS: 28.46 (4.64)	None	Treatment-naive	PPD > HC	↑ GMV in left DLPFC, right precentral gyrus and OFC	High	Yes
			HC (n = 30)	28.46 (4.64) ≥20	83.3% primiparous									
[44] Cheng et al.	Cross- sectional data from longitudinal project	GMV	PPD (n = 86)	31.25 (3.57) 21–42	1.21 (0.49)	6–8 weeks pp	DSM-5 and CCMD-3 First episode	EPDS: 16.58 (4.88)	None History of mental disorder excluded	Treatment-naive	PPD > HC	↑ regional GMV in left DLPFC and right AI	High	Yes
			HC (n = 74)	32.33 (3.81) 21–42	1.29 (0.46)									
[45] Hare et al.	Prospective cohort	GMV and CT	PPD (n = 40)	28.42 (4.87) NA	NA	10 days-10 weeks pp	DSM-IV	NA	Anxiety disorders (80%)	Medication-free	NA	Significant differences in volume in right ACC, left suborbital sulcus, right straight gyrus, and left middle-PCC No group differences in CT	High	No
			HC (n = 45)	30.00 (4.53) NA										
[46] Huang et al.	Experimen-tal	GMV	PPD (n = 52)	32.73 (3.93) NA	NA	Within 1 year pp	DSM-5	EPDS: 15.51 (5.24)	None	Unclear	PPD < HC	↓ GMV in bilateral lateral part of amygdala	High	No
			HC (n = 24)	32.41 (4.26) NA										
[47] Li et al. [48] Li et al.	Cross-sectional	CT, surface area, mean curvature and topological properties	PPD (n = 21)	31.29 (3.12) NA	NA	Within 1 year pp	DSM-5 First episode	EPDS: 16.71 (3.86)	None	Treatment-naive	PPD < HC	↓ CT in right inferior parietal lobule	High	Yes
			HC (n = 18)	31.22 (4.35) NA							PPD > HC	↑ surface area in left superior frontal gyrus, caudal middle frontal gyrus, middle temporal gyrus, insula, and right supramarginal cortex in PPD ↑ mean curvature in left superior and right inferior parietal lobule		
[49] Yang et al.	Cross-sectional	CT, local gyrification index and shape changes	PPD n = 29)	29.34 (2.62) 20–40	NA	1-2 months pp	DSM-5 First episode	EPDS: 12.21 (3.75)	None	Treatment-naive	PPD > HC	↑ CT in left superior frontal gyrus, cuneus, right lingual gyrus and fusiform gyrus Significant regional inflation in right pallidum	High	No
			HC (n = 23)	30.35 (3.42) 20–40										
*Resting-state functional MRI (fMRI)*														
[50] Chase et al.	Cross-sectional	FC in DMN	PPD (n = 14)	26.4 (5.1) NA	50% primiparous	8.1 (2.2) weeks pp	DSM-IV and HAMD score ≥15	EPDS: 14.0 (4.8)	Anxiety symptoms	Medication-free	PPD < HC	↓ PCC-right amygdala connectivity	High	Yes
			HC (n = 23)	27.7 (5.2) NA	52.2% primiparous	10.4 (1.9) weeks pp								
[51] Che et al.	Cross-sectional	fALFF and ReHo	PPD (n = 16)	31.16 (2.56) NA	NA	Within 1 year pp	DSM-5 First episode	EPDS: 16.13 (3.34)	None	Medication-free	PPD > HC	↑ fALFF in left middle frontal gyrus and DLPFC ↑ ReHo in left cerebrum (orbital part superior frontal gyrus, orbital part inferior frontal gyrus, middle frontal gyrus, precuneus, inferior parietal lobule, and superior frontal gyrus)	High	Yes
			HC (n = 16)	31.06 (4.42) NA							PPD < HC	↓ fALFF in left precentral gyrus ↓ ReHo in right cerebrum (inferior occipital gyrus and inferior frontal gyrus), bilateral precentral gyrus and left cerebellum inferior semilunar lobule		
[43] Chen et al.	Cross-sectional	FC	PPD (n = 28)	29.75 (4.49) ≥20	85.7% primiparous	6 weeks pp	DSM-IV and CCMD-3 First episode	EPDS: 28.46 (4.64)	None	Treatment-naive	PPD > HC	↑ FC of left DLPFC with right anterior cingulate and paracingulate gyri and right middle frontal gyrus, between right precentral gyrus and right median cingulate and paracingulate gyri, and between the OFC with right middle frontal gyrus and left inferior occipital gyrus	High	Yes
			HC (n = 30)	28.46 (4.64) ≥20	83.3% primiparous									
[35] Cheng et al. [36] Cheng et al. [52] Cheng et al. [37] Chen et al.	Cross-sectional data from a longitudinal project	ALFF, FCD, FC, FCS and sample entropy	PPD (n = 45)	31.11 (3.19) NA	NA	94.29 (56.29) days pp	DSM-5 and CCMD-3 First episode	EPDS: 16.2 (3.22)	None History of mental disorder excluded	Treatment-naive	PPD < HC	↓ dFC between sgACC with and left superior temporal gyrus ↓ sFC between sgACC and middle temporal gyrus ↓ rsFC between left ventral striatum and bilateral dorsomedial PFC ↓ long-range FCD in right lingual gyrus ↓ rsFC of right lingual gyrus with bilateral dorsomedial PFC and left precentral gyrus ↓ sample entropy in left medial PFC ↓ FCs between between left PCC and right paracentral lobule	High	Yes
			HC (n = 62)	32.42 (3.92) NA		96.27 (58.86) days pp					PPD > HC	↑ sFC between sgACC with ventral AI ↑ rsFC of right lingual gyrus with right angular gyrus ↑ FCS in right parahippocampus		
[53] Cheng et al.	Cross-sectional	ReHo	PPD (n = 21)	28.36 (2.18) 25–32	NA	2nd or 3rd trimester (≧ 24 weeks)	DSM-IV First episode	EPDS: 15.82 (4.46)	None History of mental disorder excluded	Treatment-naive	PPD < HC	↓ ReHo in left DLPFC, right insular and a cluster in right ventral temporal cortex, amygdala and hippocampus in PPD	High	Yes
			HC (n = 22)	28.62 (2.06) 25–32										
[54] Cheng et al.	Cross-sectional	fALFF	PPD (n = 20)	28.47 (2.48) 25–32	Primiparous	2nd or 3rd trimester (≧ 24 weeks)	DSM-IV	EPDS: 15.22 (4.56)	None History of mental disorder excluded	Treatment-naive	PPD > HC	↑ fALFF in left medial PFC, DLPFC and ACC	High	Yes
			HC (n = 22)	28.97 (2.56) 25–32							PPD < HC	↓ fALFF in bilateral OFC, right insular and cluster in right ventral temporal cortex and parahippocampus		
[55] Cheng et al.	Cross- sectional data from a longitudinal project	fALFF, ReHo and FC	PPD (n = 26)	34.5 (2.21) 21–42	NA	Within 1 year pp	DSM-5 and CCMD-3 First episode	EPDS: 17.46 (3.55)	None History of mental disorder excluded	Treatment-naive	PPD > HC	↑ fALFF in left temporal pole ↑ ReHo in sgACC and left thalamus	High	Yes
											PPD < HC	↓ fALFF in left supplementary motor area and left posterior middle temporal gyrus ↓ FC in left posterior middle temporal gyrus with precuneus and in sgACC		
			HC (n = 29)	32.48 (11.04) 21–42										
[56] Dong et al.	Cross-sectional	dALFF	PPD (n = 20)	31.05 (2.96) NA	Primiparous	Within 1 year pp	DSM-V First episode	EPDS: 17.35 (2.70)	None History of mental disorder excluded	Treatment-naive	PPD < HC	↓ dALFF in left cerebellum, right middle frontal gyrus, right inferior frontal gyrus, right precentral gyrus and right postcentral gyrus	High	Yes
			HC (n = 19)	30.95 (4.39) NA										
[57] Deligiannidis et al.	Prospective cohort	FC	PPD (n = 8)	28.62 (5.93) 18-40	25% primiparous	3–9 weeks pp	MINI	EPDS: 15.13 (5.14)	Anxiety disorders Past PPD (25%) Past MDD (87.5%)	No treatment		↓ FC between ACC and left DLPFC and bilateral amygdala; between bilateral amygdala and ACC and bilateral DLPFC; between left DLPFC and right amygdala, right hippocampus and right DLPFC	Moderate	Yes
			HC (n = 9)	30.67 (3.81) 18-40	33.3% primiparous									
[58] Deligiannidis et al.	Prospective cohort	FC	PPD (n = 23)	28.6 (4.9) 19–40	30.4% primiparous	Up to 8 weeks pp	DSM-IV	EPDS: 13.7 (3.7)	Anxiety disorder (73.9%) Past PPD (26.1%) Past MDD (82.6%)	No treatment	PPD > HC	↑ connectivity of dorsomedial PFC with DMN	Moderate	Yes
			HC (n = 28)	29.0 (5.0) 19-40	39.3% primiparous						PPD < HC	↓ connectivity of dorsomedial PFC with precuneus, posterior cingulate and postcentral gyrus and supramarginal gyrus/angular gyrus		
[46] Huang et al.	Experimen-tal	FC	PPD (n = 52)	32.73 (3.93) NA	NA	Within 1 year pp	DSM-5	EPDS: 15.51 (5.24)	None	Unclear	PPD = HC	No significant differences in FC for all four amygdala sub-regions	High	No
			HC (n = 24)	32.41 (4.26) NA										
[59] Li et al.	Cross-sectional	ReHo	PPD (n = 28)	29.27 (4.72) 21–38	53.6% primiparous	4 weeks pp	DSM-5 and CCMD-3 First episode	EPDS: 14.97 (1.66)	None	Treatment-naive	PPD > HC	↑ ReHo in left precuneus and right hippocampus ↑ FC of right hippocampus to left precuneus and left superior frontal gyrus	High	Yes
			HC (n = 29)	28.56 (4.57) 21–38	48.3% primiparous						PPD < HC	↓ ReHo in left DLPFC and right insula		
[60] Mao et al.	Cross-sectional	preferred information flow direction	PPD (n = 21)	31.65 (1.85) NA	NA	Within 1 year pp	DSM-5 First episode	EPDS: 16.53 (3.02)	None History of mental disorder excluded	Treatment-naive	PPD < HC	↓ preferred information flow direction from right superior frontal orbital part gyrus to left insula, left middle cingulum gyrus to right supramarginal gyrus, and from left middle temporal to right amygdala	High	No
			HC (n = 23)	31.13 (2.88) NA										
[61] Xiao-juan et al.	Cross-sectional	ReHo	PPD (n = 10)	27.58 (4.56) NA	NA	Within 16 weeks pp	DSM-IV and CCMD-3 First episode	NA	None	Treatment-naive	PPD > HC	↑ ReHo in posterior cingulate, cingulate gyrus, frontal lobe, parietal lobe, medial frontal gyrus and medial frontal gyrus	Moderate	Yes
			HC (n = 11)	27.16 (3.68) NA							PPD < HC	↓ ReHo in inferior temporal gyrus, middle temporal gyrus, superior temporal lobe and frontal lobe		
[62] Xu et al.	Cross-sectional	ALFF, DC and ReHo	PPD (n = 52)	32.73 (3.93) NA	NA	4.85 (3.76) months pp	DSM-5	EPDS: 15.51 (5.24)	None	No treatment	PPD > HC	↑ ALFF in left calcarine ↑ DC in left fusiform gyrus ↑ ReHo in middle occipital gyrus	High	Yes
			HC (n = 24)	32.41 (4.26) NA		6.22 (4.30) months pp					PPD < HC	↓ ALFF in left cerebellum and right ACC ↓ DC in right middle cingulate cortex ↓ ReHo in right ACC		
[63] Zhang et al.	Experimen-tal	VMHC	PPD (n = 31)	31.5 (3.4) NA	NA	NA pp	DSM-IV	EPDS: 16.7 (4.6)	None	No treatment at baseline	PPD < HC	↓ VMHC in bilateral insula, amygdala, medial frontal gyrus, putamen, pallidum, ACC and middle cingulate cortex	High	Yes
			HC (n = 31)	31.7 (6.3) NA										
[64] Zhang et al.	Cross-sectional	DC and FC	PPD (n = 29)	27.24 (3.55) 21–38	86.2% primiparous	4 weeks pp	DSM-5 and CCMD-3 First episode	EPDS: 15.79 (1.86)	None History of mental disorder excluded	Treatment-naive	PPD > HC	↑ DC in right hippocampus and left inferior frontal orbital gyrus ↑ FC of left inferior frontal orbital gyrus with right superior frontal gyrus	High	Yes
			HC (n = 30)	27.33 (4.10) 21–38	86.7% primiparous						PPD < HC	↓ FC of right hippocampus with right middle frontal gyrus and left median cingulate and paracingulate gyri ↓ FC of left inferior frontal orbital gyrus with left fusiform		
[65] Zhang et al.	Cross-sectional	VMHC	PPD (n = 26)	27.46 (4.15) 22–37	92.3% primiparous	4 weeks pp	DSM-5 and CCMD-3 First episode	EPDS: 14.84 (1.51)	None History of mental disorder excluded	Treatment-naive		↓ VMHC in bilateral dorsomedial PFC, ACC and OFC	High	Yes
			HC (n = 25)	27.0 (4.02) 21–37	88% primiparous									
*Task-based fMRI*														
[66] Dudin et al.	Cross-sectional	Affect rating task	PPD (n = 32)	30.13 (5.04) 20–40	65.6% primiparous	2‐5 months pp	DSM-IV-TR	EPDS: 7.38 (5.27)	None	Antidepressant or psychotherapy	PPD > HC	↑ right amygdala response to unfamiliar smiling infants	High	No
			HC (n = 25)	29.44 (4.20) 20–40	68% primiparous									
[67] Finnegan et al.	Prospective cohort	viewing infants during emotion-eliciting tasks	PPD (n = 2)	26.7 (3.9) 19–33	NA	3 months pp	DSM-IV	NA	Anxiety disorder (n = 4 current, n = 2 past)	Medication free	PPD < HC	↓ differential response in right dorsolateral superior, middle, and inferior frontal gyri, left inferior and middle temporal lobe, and bilateral angular gyri	High	No
			HC (n = 18)											
[68] Lenzi et al.	Cross-sectional	own / unknown child faces	PPD (n = 14)	31.5 (4.8) 23–42	Primiparous	7–12 months pp	CES-D > 20	CES-D: 29.4 (5.56)	None	No treatment	PPD > HC	↑ deactivation (greater activity during rest vs task) in orbital and medial PFC ↑ right amygdala reactivity	Moderate	Yes
			HC (n = 16)											
[69] Moses-Kolko et al.	Cross-sectional	Card-guessing task with monetary reward	PPD (n = 12)	27.5 (4.7) NA	50% primiparous	8.4 (2.1) weeks pp	DSM-IV	EPDS: 14.9 (4.5)	Anxiety disorders	No treatment	PPD > HC	No difference in left ventral striatum activity with reward between groups ↑ nonlinear attenuation of left ventral striatal activity after reward	High	Yes
			HC (n = 12)	28.6 (6.4) NA		10.3 (2.3) weeks pp								
[70] Moses-Kolko et al.	Cross-sectional	Adult emotional face matching task	PPD (n = 14)	26.8 (6.1) NA	50% primiparous	4–13 weeks pp	DSM-IV	EPDS: 14.7 (4.3)	Anxiety disorders Past PPD or MDD (n = 10)	No treatment	PPD < HC	↓ left dorsomedial PFC activity to negative emotional faces ↓ Left amygdala activity	High	Yes
			HC (n = 16)	26.7 (4.8) NA	37.5% primiparous									
[71] Silverman et al.	Cross-sectional	Word/non-word task for emotional valanced words	PPD (n = 6)	27 NA	BA	6–8 weeks pp	EPDS > 12 indicating probable depression DSM-IV	Mild or above	None History of mental disorder excluded	No treatment	PPD < HC	↓ right amygdala activation with negative stimuli	High	Yes
			HC (n = 11)											
[21] Silverman et al.	Cross-sectional	Word/non-word task for emotional valanced words	PPD (n = 4)	28 NA	NA	7–8 weeks pp	EPDS > 12 indicating probable depression	EPDS: 15.3	None History of mental disorder excluded	No treatment	PPD > HC	↑ activity in bilateral insula to negative stimuli	High	Yes
			HC (n = 4)								PPD < HC	↓ activity in bilateral OFC, right amygdala, precentral gyrus, cingulate, putamen, inferior temporal gyrus, fusiform, precuneus, DLPFC, superior temporal gryus and ACC to negative stimuli ↓ activity in striatum, cingulate gyrus, DLPFC and precentral gyrus to positive stimuli		
[72] Wonch et al.	Cross-sectional	own / unknown infant faces and non-infant images	PPD (n = 28)	30.64 (SEM = 0.93) 20–40	64.4% primiparous	2–5 months pp	DSM-IV-TR	EPDS: 8.29 (0.84)	Anxiety symptoms	Antidepressants	PPD > HC	↑ BOLD response across conditions in right amygdala	High	Yes
			HC (n = 17)	29.18 (SEM = 1.19) 20–40	70.59% primiparous						PPD < HC	↓ bilateral amygdala–right insular cortex connectivity with own-other infant faces		
*Functional near-infrared spectroscopy (fNIRS)*														
[73] Morgan et al.	Cross-sectional	mother-infant interaction in vivo	PPD (n = 11)	30.30 (4.19) 23–38	39.1% primiparous	1 year pp	depressive symptoms according to CES-D or DSM-IV	CES-D: 22.64 (8.71)	Anxiety disorders	Antidepressants	-	↑ depression severity associated with ↓ connectivity between right temporoparietal junction and lateral PFC ↑ depression severity associated with ↑ connectivity between right temporoparietal junction and anterior medial PFC	High	No
			HC (n = 12)	32.33 (2.81) 23-38										
[74] Song et al.	Cross-sectional	verbal fluency task	PPD likely (n = 55)	31.04 (4.10) NA	Between 1-3 children	42 days pp	EPDS: depression likely (≥11), possible depression (5–10), depression not likely (0–4)	NA	Yes (n = 21), but not specified	NA	PPD = HC	No statistically significant difference in integral or centroid values between subgroups of depression	Moderate	No
			Possible PPD (n = 12)											
			PPD not likely (n = 42)											
*Magnetic resonance spectroscopy (MRS)*														
[58] Deligiannidis et al.	Prospective cohort	GABA/Cr concentrations	PPD (n = 23)	28.6 (4.9) 19–40	30.4% primiparous	Up to 8 weeks pp	DSM-IV	EPDS: 13.7 (3.7)	Anxiety disorder (73.9%) Past PPD (26.1%) Past MDD (82.6%)	No treatment	PPD = HC	No difference between groups in pregenual anterior cingulate or occipital cortices GABA/Cr concentrations	Moderate	No
			HC (n = 28)	29.0 (5.0) 19–40	39.3% primiparous									
[75] De Rezende et al.	Cross-sectional	Cr levels	PPD (n = 20)	28.2 (4.8) NA	20% primiparous	mean 21.8 weeks pp	DSM-IV	EPDS: 16.65 (6.18)	Anxiety disorders	Treatment-naive	PPD = HC	No significant differences between groups in metabolite levels in anterior cingulate gyrus	High	No
			HC (n = 19)	28.8 (4.3) NA	31.6% primiparous									
[76] Epperson et al.	Cross-sectional	GABA levels	PPD (n = 9)	30 (5.3) NA	1.9 (0.9)	Within 6 months pp	clinical interview and HAMD ≥ 18	HAMD: 20.6 (2.6)	None Past MDD (n = 3) Past PPD (n = 1)	Medication-free for at least 9 months prior	PPD < HC	trend: ↓ cortical GABA levels	High	No
			HC (n = 14)	31 (2.9) NA	1.6 (0.6)									
[77] McEwen et al.	Cross-sectional	Glu levels	PPD (n = 12)	28.67 (7.45) NA	NA	3 weeks-3 months pp	DSM-IV-TR	NA	None Past MDD (n = 5)	No treatment	PPD > HC	↑ Glu levels in medial PFC no significant differences for other medial PFC metabolite levels	High	No
			HC (n = 12)	29.08 (4.89) NA										
[78] Rosa et al.	Cross-sectional	Glu/Glx and NAA levels	PPD (n = 33)	27.7 (4.8) NA	NA	mean 18.5-19.3 weeks pp	DSM-IV	moderate (55%) mild (24%) severe (21%)	Anxiety (52%) Previous MDD (64%)	Antidepressants	PPD < HC	↓ Glx and NAA levels in left DLPFC	High	No
			HC (n = 25)	29.0 (6.0) NA										
*Positron emission tomography (PET)*														
[79] Sacher et al.	Cross-sectional	MAO-A	PPD (n = 15)HC (n = 15)	30.32 (6.25) NA29.93 (5.54) NA	46.7% primiparous60% primiparous	within 18 months pp	DSM-IV First episode	HRSD: 20.8 (3.17)	None	Antidepressant naive	PPD > HC	↑ MAO-A VT in PFC and ACC	High	No
[80] Moses-Kolko et al.	Cross-sectional	striatal D2/3 receptor binding potential (BPND)	PPD (n = 13)	29.9 (7.5) NA	30.8% primiparous	10.6 (2.5) weeks pp	DSM-IV	EPDS: 14.0 (5.2)	Anxiety symptoms	Medication-free for 3 weeks	PPD = HC	No differences in D2/3 receptor BPND between groups	High	No
			HC (n = 13)	30.8 (5.6) NA										

*FA* fractional anisotropy, *AD* axial diffusivity, *RD* radial diffusivity, *PPD* peripartum depression, *HC* healthy controls, *pp* postpartum, *DSM* diagnostic and statistical manual of mental disorders, *CCMD-3* chinese classification of mental disorders, *EPDS* edinburgh postnatal depression scale, *MK* mean kurtosis, *MD* mean diffusivity, *NA* not available, *MDD* major depressive disorder, *GMV* gray matter volume, *DLPFC* dorsolateral prefrontal cortex, *OFC* orbitofrontal cortex, *AI* anterior insula, *CT* cortical thickness, *MINI* mini international neuropsychiatric interview, *ACC* anterior cingulate cortex, *PCC* posterior cingulate cortex, *FC* functional connectivity, *DMN* default-mode network, *HAMD/HDRS* hamilton depression rating scale*, fALFF* fractional amplitude of low-frequency fluctuations, *ReHo* regional homogeneity, *FCD* FC density, *FCS* FC strength, *dFC* dynamic FC, *sgACC* subgenual ACC, *sFC* static FC, *DC* degree centrality, *VMHC* voxel-mirrored homotopic connectivity, *CES-D* center for epidemiologic studies depression scale, *GABA* gamma-aminobutyric acid, *Cr* creatine, *Glu* Glutamate, *NAA* N-Acetyl-aspartate, *Glu/Glx* glutamate and glutamine (Glx), *MAO-A* monoamine oxidase-A.

**Table 2:** Summary of study characteristics and results of included female non-peripartum MDD studies.

Reference	Design	Paradigm/Measure	Group	Age (M, SD, range)	Pregnancy history	Diagnostic criteria	Severity (M, SD)	Comorbidity	Treatment status	Direction of effect	Main finding	Study quality	Included in ALE (yes/no)
*Diffusion tensor (DTI), kurtosis imaging (DKI) or diffusion-weighted imaging (DWI)*													
[81] Domain et al.	Cross-sectional	FA	fMDD (n = 26)	46.69 (11) NA	Current pregnancy excluded	DSM-IV-TR	MADRS: 29.92 (6.8)	None	Medication, ECT and neuromodulation	fMDD < HC	↓ FA in widespread white matter (forceps minor and major, bilateral inferior fronto-occipital fasciculus, bilateral uncinate fasciculi, inferior longitudinal fasciculi and superior longitudinal fasciculi)	High	No
			HC (n = 25)	49.5 (9.5) NA									
[82] Lyon et al.	Experimental	FD. FDC, FA, AD	fMDD (n = 115)	33.6 (11.7)* NA	Unclear	DSM-IV	HDRS-17: 21.4 (3.7)*	None	Antidepressant-free	fMDD < HC	↓ FDC in left and right frontal projection of corpus callosum ↓ FC in left and right frontal projection of corpus callosum, right anterior limb of internal capsule, tapetum and right inferior longitudinal fasciculus ↓ FA in gen of corpus callosum, bilateral cerebral penduncle and left uncinate fasciculs ↓ AD in left frontal projection of corpus callosum	High	Yes
			HC (n = 14)	30.3 (12.8)* NA									
*Structural magnetic resonance imaging (MRI)*													
[83] Carceller-Sindreu et al.	Cross-sectional	Volume	fMDD (n = 14)	NA	Unclear	DSM-IV-TR	NA	NA	Medication	fMDD > HC	↑ habenula white matter volumes	High	No
			HC (n = 24)	NA									
[84] Depping et al.	Cross-sectional	GMV	fMDD (n = 22)	33.5 (8.9) NA	Unclear (did not collect information)	DSM-IV	HAMD: 28.4 (4.7) BDI: 28.7 (8.9)	None	Medication and psychotherapy	fMDD < HC	↓ GMV in ACC and medial PFC	High	Yes
			HC (n = 22)	31.4 (11.2) NA									
[81] Domain et al.	Cross-sectional	CT	fMDD (n = 26)	46.69 (11) NA	Current pregnancy excluded	DSM-IV-TR	MADRS: 29.92 (6.8)	None	Medication, ECT and neuromodulation	fMDD = HC	No significant differences	High	No
			HC (n = 25)	49.5 (9.5) NA									
[85] Hastings et al.	Cross-sectional	Volume	fMDD (n = 10)	NA	Current pregnancy excluded	DSM-III-R	NA	None	NA	fMDD < HC	↓ amygdala volume	High	No
			HC (n = 10)	NA									
[86] Hu et al.	Cross-sectional	SA, CV	fMDD (n = 78)	32.7 (11.8) NA	Current pregnancy excluded	DSM-IV	HAMD: 27.7 (5.4)	None	Treatment-naive	fMDD < HC	↓ SA in left ventrolateral PFC and ↓ CV in right rostromedial PFC	High	Yes
			HC (n = 51)	35.2 (11.1) NA									
[87] Kim et al.	Cross-sectional	GMV	fMDD (n = 22)	38.5 (9.7) 21–55	Unclear (did not collect information)	DSM-IV	BDI: 22.3 (13.61)	None	Antidepressants	fMDD < HC	↓ GMV in bilateral caudate extending into anterior nucleus of thalamus	High	Yes
			HC (n = 25)	35.3 (11.3) 23–56						fMDD = HC	No group differences within the amygdala, hippocampus, subgenual ACC, globus pallidus and putamen		
[88] Kong et al.	Cross-sectional	GMD	fMDD (n = 16)	28.88 (9.71) 18–45	Unclear	DSM-IV	HDRS: 29.56 (5.11)	None	Medication -naive	fMDD < HC	↓ GMD in bilateral amygdala and hippocampus	High	Yes
			HC (n = 17)	28.00 (9.09) 18–44									
[89] Mak et al.	Cross-sectional	GMV and GMC	fMDD (n = 17)	45.5 (8.5) NA	Unclear	ICD-10	BDI-II: 29.7 (8.5)	None	Medication	fMDD < HC	↓ GMC in right anterior cingulate gyrus, right SFG, right medial SFG, left MFG, right inferior orbitofrontal gyrus, right precentral gyrus, right STG, left middle temporal gyrus, right fusiform gyrus and left precuneus ↓ GMV in right anterior cingulate gyrus, right precentral gyrus, right supplementary motor area, right superior temporal pole gyrus, left middle temporal gyrus, left angular gyrus and left precuneus	High	Yes
			HC (n = 17)	45.8 (9.8) NA									
[90] Siragusa et al.	Cross-sectional	Volume	fMDD (n = 25)	39.1 (11.4) 18–55	Current pregnancy excluded	DSM-IV	MADRS: 31.5 (5.2)	NA	Medication	fMDD < HC	↓ amygdala volume	High	No
			HC (n = 25)	39.7 (11.3) 18–55									
[91] Tang et al.	Cross-sectional	GMV	fMDD (n = 14)	29.5 (6.8) NA	Unclear	DSM-IV	NA	Anxiety disorders	Medication-free	fMDD < HC	↓ GMV in bilateral ventral ACC and right amygdala	High	Yes
			HC (n = 13)	29.5 (6.9) NA									
[92] Yang et al.	Cross-sectional	GMV	fMDD (n = 53)	30.2 (10.8) 18–55	Current pregnancy excluded	DSM-IV-TR	HAMD: 23.00 (4.11)	None	Medication-free	fMDD < HC	↓ GMV in left lingual gyrus extending to parahippocampal gyrus, dorsal medial prefrontal gyrus extending to supplementary motor area and cerebellum	High	Yes
			HC (n = 53)	29.1 (9.1) 18–55									
[93] Yang et al.	Cross-sectional	GMV	fMDD (n = 35)	44.5 (11.2) 18–60	Unclear	DSM-IV	HAMD: 28.29 (7.99)	Anxiety symptoms	Antidepressant-free	fMDD < HC	↓ GMV in right amygdala, right parahippocampus gyrus, bilateral insula, bilateral putamen, left lingual gyrus, cerebellum, and caudal middle-frontal region	High	Yes
			HC (n = 23)	39.1 (14.4) 18–60									
*Resting-state functional MRI (fMRI)*													
[94] Amiri et al.	Cross-sectional	FC (nodal degree)	fMDD (n = 21)	NA	Unclear	NA	Severe	None	Previous antidepressants	fMDD > HC	↑ degree values in the left and right ventral caudate, left lateral habenula, and right nucleus accumbens	Moderate	No
			HC (n = 18)	NA									
[95] Belleau et al.	Cross-sectional	CAPs	fMDD (n = 35)	NA 20–45	Unclear	DSM-IV	BDI-II: 27.44 (7.68)	Anxiety disorders	Medication-free	fMDD > HC	↑ time in a posterior DMN-FPN CAP and transitioned more frequently between posterior DMN-FPN and prototypical DMN	High	No
			HC (n = 36)	NA 20–45									
[96] Chen et al.	Cross-sectional	ALFF and FC	fMDD (n = 16)	24.26 (3.17) 18–35	Current pregnancy and breastfeeding excluded	DSM-5	HAMD: 30.20 (4.97)	None	Medication-free	fMDD < HC	↓ ALFF in right postcentral gyrus	High	Yes
			HC (n = 20)	23.40 (3.16) 18–35						fMDD > HC	↑ FC between left MFG and bilateral putamen		
[55] Cheng et al.	Cross-sectional data from longitudinal project	fALFF, DC, ReHo	fMDD (n = 22)	37.55 (11.5) 21-42	Unclear	DSM-5 and CCMD-3	HRSD: 23.95 (4.64)	None	Treatment-naive	fMDD > HC	↑ fALFF in left temporal pole ↑ DC in right cerebellum ↑ ReHo in left sgACC and left thalamus	High	Yes
			HC (n = 29)	32.48 (11.04) 21–42									
[97] Dong et al.	Cross-sectional	CAPs	fMDD (n = 83)	25.66 (7.79) NA	Unclear	DSM-IV-TR	HAMD: 22.69 (4.80)	None	Medication-free	fMDD < HC	↓ persistence in the DMN + SN- CAP (activation of the DMN and deactivation of the SN)	High	No
			HC (n = 137)	21.09 (3.32) NA									
[98] Li et al.	Cross-sectional	fALFF and ReHo	fMDD (n = 57)	32.84 (9.31) 18–50	Current pregnancy and breastfeeding excluded	DSM-IV-TR	HAMD: 14.95 (8.00)	None	Medication	fMDD = HC	No differences in fALFF and ReHo in ACC and insula	High	No
			HC (n = 121)	33.95 (11.62) 18–50									
[99] Mei et al.	Cross-sectional	ALFF	fMDD (n = 36)	36.1 (10.7) 18–60	Current pregnancy and breastfeeding excluded	DSM-IV	HAMD: 23.9 (4.5)	None	Medication-free	-	Positive association between mean ALFF of right caudate nucleus with illness duration	High	No
			HC (n = 36)	36.6 (11.8) 18–60									
[100] Pessin et al. [101] Philippi et al.	Cross-sectional	BOLD signal variability	fMDD (n = 34)	27.9 (7.1) 18–45	Recent pregnancy or breastfeeding (within the last 6 months) excluded	DSM-5	BDI-II: 20.3 (10.8)	None	Antidepressant-free	fMDD < HC	↓ BOLD signal variability in right and left cerebellum and DLPFC	High	Yes
			HC (n = 30)	27.1 (7.6) 18–45									
[102] Sun et al.	Cross-sectional	ALFF	fMDD (n = 18)	42.16 (10.34) 18–60	Current pregnancy excluded	DSM-5	HAMD-17: 23.72 (3.26)	None	Previous antidepressants	fMDD > HC	↑ ALFF in left MFG and left precentral gyrus	High	Yes
			HC (n = 19)	43.68 (10.98) 18–60									
[103] Tang et al.	Cross-sectional	FC	fMDD (n = 12)	35.4 (9.5) NA	Current pregnancy excluded	ICD-10	HAMD: 23.00 (2.90)	None	Antidepressants	fMDD < HC	↓ FC between left hippocampus and temporo-occipital areas including bilateral lingual gyrus and fusiform	High	Yes
			HC (n = 12)	35.1 (8.5) NA									
[104] Teng et al.	Cross-sectional	ALFF and FC	fMDD (n = 25)	35.8 (8.9) 20–50	Unclear	DSM-IV	HAMD: 25.67 (5.31)	None	Medication-free	fMDD < HC	↓ ALFF in left middle occipital gyrus ↓ FC between left middle occipital gyrus and left OFC	High	Yes
			HC (n = 13)	38.2 (10.1) 20–50						fMDD > HC	↑ FC between left middle occipital gyrus and left medial prefrontal gyrus and left hippocampus		
[105] Tu et al.	Cross-sectional	ReHo and ALFF	fMDD (n = 47)	29.98 (10.18) 18–51	Current pregnancy excluded	DSM-IV	HAMD-17: 26.34 (6.04)	None	Medication-naive	fMDD < HC	↓ ALFF in right superior occipital gyrus ↓ ReHo in left calcarine and left dorsolateral superior frontal gyrus	High	Yes
			HC (n = 47)	28.96 (10.18) 18–51									
[93] Yang et al.	Cross-sectional	FC	fMDD (n = 35)	44.5 (11.2) 18–60	Unclear	DSM-IV	HAMD: 28.29 (7.99)	Anxiety symptoms	Antidepressant- free	fMDD < HC	↓ FC of right amygdala with the ventrolateral PFC, bilateral insula, and bilateral putamen	High	Yes
			HC (n = 23)	39.1 (14.4) 18–60									
[106] Zhang et al.	Cross-sectional	ALFF	fMDD (n = 11)	34.1 (8.8) NA	Unclear	ICD-10	HAMD: 22.91 (3.015)	None	Antidepressants	fMDD < HC	↓ ALFF in right putamen and right middle temporal gyrus	High	Yes
			HC (n = 11)	33.6 (7.2) NA						fMDD > HC	↑ ALFF in left mPFC and left MFG		
*Task-based fMRI*													
[107] Abler et al.	Cross-sectional	positive, negative and neutral pictures	fMDD (n = 12)	41.2 (NA) 23–51	Unclear	ICD-10	Moderate or severe	None	Antidepressants	fMDD > HC	↑ activation in left and right sublenticular extended amygdala for expectation ↑ activation in sgACC and dorsal ACC for presentation	High	Yes
			HC (n = 12)	40.7 (NA) 23–54									
[108] Almeida et al.	Cross-sectional	emotional dynamic face processing task	fMDD (n = 12)	30.3 (7)* NA	Current pregnancy excluded	DSM-IV	HRSD-25: 28.1 (6.25)*	Anxiety disorders	Medication	fMDD < HC	↓ positive left-sided ventromedial PFC–sgACC connectivity to happy faces	High	No
			HC (n = 12)	31.8 (6.8)* NA						fMDD > HC	↑ inverse left-sided ventromedial PFC – amygdala connectivity to happy faces ↑ inverse left-sided sgACC – amygdala connectivity to happy faces ↑ positive left-sided sgACC – amygdala connectivity to fearful faces		
[109] Baeken et al.	Cross-sectional	emotionally valenced baby faces	fMDD (n = 12)	36.0 (10.9) NA	n = 5 mothers	MINI	BDI: 28.0 (9.7)	NA	Medication-free	fMDD > HC	↑ activity in bilateral sgACC in both emotional conditions (approach and withdrawal)	High	No
			HC (n = 12)	30.2 (8.1) NA	n = 3 mothers								
[110] Bär et al.	Cross-sectional	Painful stimuli	fMDD (n = 13)	35.9 (11.4) NA	Unclear (did not collect information)	DSM-IV	HAMD: 23.69 (5.19)	None	Antidepressant-free for 8 weeks prior	fMDD > HC	↑ BOLD signal in left ventrolateral thalamus, right ventrolateral PFC and DLPFC	High	Yes
			HC (n = 13)	34.3 (10.5) NA									
[111] Briceño et al.	Cross-sectional	Facial emotion perception	fMDD (n = 24)	37.8 (14.5) NA	Unclear	DSM-IV	HDRS-17: 15.8 (7.2)	None	Antidepressants	fMDD < HC	↓ activation in right parahippocampal	High	Yes
			HC (n = 22)	31.7 (14.4) NA						fMDD > HC	↑ activation in bilateral SFG, MFG and precentral gyri, anterior, dorsal, and posterior cingulate, lingual gyrus, STG and middle temporal gyrus, middle occipital gyrus, cuneus, putamen, pulvinar and substantia nigra		
[112] Briceño et al.	Cross-sectional	Facial Emotion Perception	fMDD (n = 15)	29.2 (7.8) NA	Unclear	DSM-IV	HDRS: 17.4 (4.3)	NA	Medication	fMDD > HC	↑ activity in Inferior/MFG, MFG, MFG/Cingulate and Cingulate	Moderate	Yes
			HC (n = 19)	26.4 (7.7) NA									
[113] Cane et al.	Cross-sectional	Go/No Go task	fMDD (n = 16)	30.56 (9.35) 19–47	Current pregnancy excluded	DSM-5	NA	NA	NA	fMDD = HC	No differences between groups	High	No
			HC (n = 21)	27.71 (8.28) 18–48									
[114] Dong et al.	Cross-sectional	Montreal Imaging Stress Task	fMDD (n = 76)	25.68 (7.90) NA	Unclear	DSM-IV-TR	HAMD: 22.49 (4.69)	None	Medication-free	fMDD < HC	↓ deactivation in amygdala, nucleus accumbens, hippocampus, and amygdala-nucleus accumbens-ACC network ↓ deactivation over time over stress exposure in amygdala, medial OFC and nucleus accumbens	High	No
			HC (n = 137)	21.33 (4.28) NA									
[115] Ironside et al.	Cross-sectional	acute laboratory stressor task	fMDD (n = 18)	21.06 (1.76) 18–25	Unclear	DSM-5	HDRS: 17.3 (4.24)	NA	Medication-free	fMDD < HC	↓ activation in FPN and SN, irrespective of stress	High	No
			HC (n = 17)	21.53 (2.45) 18–25									
[116] Kumari et al.	Cross-sectional	Cognitive generation of affect	fMDD (n = 6)	47.0 (3.6) 36–52	Unclear	DSM-IV	HAMD: 19.33 (1.03)	None	Medication or ECT	fMDD < HC	↓ activation in ACC, left posterior cingulate gyrus, left insula/striatum, bilateral cerebellum to negative stimuli ↓ activation in left MFG/anterior cingulate gyrus, right STG, left posterior cingulate/precuneus, left cerebellum to positive stimuli ↓ activation in anterior cingulate gyrus, left hippocampus and postcentral gyrus (positive vs negative)	Moderate	Yes
			HC (n = 6)	44.0 (2.4) 32–55						fMDD > HC	↑ activation in right inferior temporal gyrus, left middle temporal gyrus, and left precuneus to negative stimuli ↑ activation in the right PFC, right parahippocampal gyrus, right sgACC, right caudate nucleus/putamen, and left inferior frontal gyrus to positive stimuli ↑ activation in right parahippocampal gyrus, left brain stem, left inferior frontal gyrus, middle occipital gyrus and right pulvinar thalamus (positive vs negative)		
[117] Malejko et al.	Cross-sectional	Go/no go task	fMDD (n = 16)	28.7 (1.2) 18–38	Unclear	DSM-5	BDI: 33.63 (2.87)	Dysthymia (n = 2)	Antidepressants	fMDD > HC	↑ differential neural activations due to commission error within pre-supplementary motor area and dorsal ACC	High	Yes
			HC (n = 17)	23.1 (1.0) 18–38									
[118] Malejko et al.	Cross-sectional	parametric electric stimulation	fMDD (n = 12)	NA > 18	Unclear	DSM-IV	NA	None	Antidepressants	fMDD < HC	↓ activity in somatosensory cortex, posterior insula and dorsal ACC/supplementary motor area	High	Yes
			HC (n = 15)	NA > 18									
[119] Mitterschiffthaler et al.	Cross-sectional	Positive valence and neutral images	fMDD (n = 7)	46.3 (8.1) NA	Unclear	DSM-IV	HAMD: 19.4 (1)	None	Antidepressants	fMDD < HC	↓ activation in right precentral gyrus, precuneus, right inferior parietal gyrus, left medial frontal gyrus and left and right lingual gyrus	Moderate	Yes
			HC (n = 7)	48.3 (10.1) NA						fMDD > HC	↑ activation in right IFG, left superior and middle temporal gyrus, thalamus, ACC, PCC and left insula		
[120] Robert et al.	Cross-sectional	Variable Attention Affective Task	fMDD (n = 30)	47.6 (11) NA	Current pregnancy excluded	DSM-IV	NA	None	Medication and neuromodulation	fMDD < HC	↓ FC between left amygdala and left ACC during negative stimuli ↓ FC between right DLPFC and right ACC during high-attention stimuli	High	Yes
			HC (n = 25)	46.7 (10.4) NA						fMDD > HC	↑ FC between right DLPFC and right amygdala during high-attention stimuli		
[121] Shao et al.	Cross-sectional	Modified trust game	fMDD (n = 14)	39.7 (2.1) 25–55	Unclear	DSM-IV	BDI-II: 25.50 (3.36)	None	Antidepressants	fMDD < HC	↓ activity in dorsal putamen, anterior insula and DLPFC during low-risk cheating vs benevolent choices	High	Yes
			HC (n = 15)	41.0 (2.7) 25–55						fMDD > HC	↑ activity in parietal, occipital and frontal motor areas during cheating choices under high versus low risk		
[122] Tak et al.	Cross-sectional	emotional negative and neutral images	fMDD (n = 34)	24.5 (2.8) 20–31	Current pregnancy and breastfeeding excluded	DSM-5	HAM-D: 19.9 (6.1)	None	Medication-free	fMDD < HC	↓ activation of bilateral OFC	High	Yes
			HC (n = 28)	24.4 (2.6) 20–29						fMDD > HC	↑ activation of left parahippocampal gyrus		
[123] Wagner et al.	Cross-sectional	Stroop	fMDD (n = 16)	40.3 (9.7) 18–55	Unclear	DSM-IV	HRSD: 23.5 (4.9)	None	Medication-free	fMDD > HC	Incongruent condition: ↑ activation in rostral part of anterior cingulate gyrus and left ventrolateral PFC Incongruent > congruent contrast: ↑ activation in left DLPFC	High	Yes
			HC (n = 16)	38.8 (9.1) 18–55									
[124] Young et al.	Cross-sectional	Autobiogra-phical memory task	fMDD (n = 20)	35.5 (8.13) 18–55	Current pregnancy excluded	DSM-IV-TR	HDRS: 19.6 (6.63)	NA	Medication-free	fMDD < HC	↓ activity in right caudate during recall of positive specific memories	High	Yes
			HC (n = 20)	NA 18–55						fMDD > HC	↑ activity in PCC, insula and thalamus during recall of negative specific memories		
[125] Yttredahl et al.	Cross-sectional	Social feedback task	fMDD (n = 19)	29.6 (11.0) 18–55	Unclear	DSM-IV	HDRS-17: 14.8 (3.0)	None	Antidepressants	fMDD > HC	↑ activity in the left dorsal ACC during rejection	High	Yes
			HC (n = 19)	29.8 (11.1) 18–55									
*Functional near-infrared spectroscopy (fNIRS)*													
[126] Lyu et al.	Cross-sectional	verbal fluency task	fMDD (n = 75)	29.2 (7.9)* 18–45	Current pregnancy excluded	DSM-IV	HAMD: 23.01 (4.15)*	None	Antidepressants	-	Significant correlation between HAMD scores and [oxy-Hb] changes in the right frontal brain region	High	No
			HC (n = 48)	27.8 (8.5)* 18–45									
[127] Ma et al.	Cross-sectional	verbal fluency task	fMDD (n = 30)	37.5 (10.6) 18–60	Unclear	DSM-IV	HAMD: 22.93 (10.16)	None	Antidepressants	fMDD < HC	↓ Oxy-Hb activation in DLPFC	High	No
			HC (n = 30)	34.8 (8.8) 18–60									
*Magnetic resonance spectroscopy (MRS)*													
[115] Ironside et al.	Cross-sectional	GABA	fMDD (n = 17)	21.06 (1.76) 18–25	Unclear	DSM-5	HDRS: 17.3 (4.24)	NA	Medication-free	fMDD < HC	↓ GABA in rostral ACC	High	No
			HC (n = 13)	21.53 (2.45) 18–25									
[128] Kantrowitz et al.	Cross-sectional	GABA, Glx and NAA	fMDD (n = 22)	38.4 (11.0) NA	Unclear	DSM-IV	MADRS: 30.1 (3.4)	None	Medication-free	-	Positive correlation between MADRS and Glx/NAA	High	No
			HC (n = 13)	30.7 (5.1) NA									
[129] Song et al.	Cross-sectional	Taurine	fMDD (n = 41)	22.02 (SEM = 0.44) 18–28.5	Current pregnancy excluded	DSM-5	HDRS-17: 20.46 (0.91)	None	Medication-free	fMDD < HC	↓ taurine concentration in hippocampus, but not in ACC or occipital cortex	High	No
			HC (n = 43)	22.62 (SEM = 0.39) 18–27.85									
[103] Tang et al.	Cross-sectional	tCho, tNAA and tCr	fMDD (n = 12)	35.4 (9.5) NA	Current pregnancy excluded	ICD-10	HAMD: 23.00 (2.90)	None	Antidepressants	fMDD > HC	↑ tCho levels in left hippocampus, but not tNAA or tCr	High	No
			HC (n = 12)	35.1 (8.5) NA									
[130] Tran et al.	Cross-sectional	Glu and GABA+	fMDD (n = 13)	NA reproductive age	Current pregnancy excluded	DSM-5	moderate to severe	NA	Medication-free	fMDD = HC	no differences in medial PFC Glu or GABA+	High	No
			HC (n = 13)										
[131] Tran et al.	Cross-sectional	GABA+	fMDD (n = 13)	31.55 (8.90) 18–48	Current pregnancy excluded	DSM-5	BDI: median 27.0	NA	Medication-free	fMDD < HC	↓ GABA+ in left DLPFC ↓ ratios of GABA+ to glutamate in the left DLPFC	High	No
			HC (n = 20)	31.46 (9.66) 18–47						fMDD = HC	No differences in glutamate levels in the left DLPFC		
[132] Zhang et al.	Experimental	NAA/Cr, Cho/Cr	fMDD (n = 17)	43 (10) NA	Unclear	DSM-IV	NA	None	Antidepressant-free for 8 weeks prior	fMDD < HC	↓ Cho/Cr in bilateral ventral prefrontal white matter	High	No
			HC (n = 19)	41 (10) NA						fMDD = HC	No differences in NAA/Cr levels in bilateral ventral prefrontal white matter		
[106] Zhang et al.	Cross-sectional	Glu, GABA and Glx	fMDD (n = 11)	34.1 (8.8) NA	Unclear	ICD-10	HAMD: 22.91 (3.015)	None	Antidepressants	fMDD = HC	No differences in Glu, GABA and Glx concentrations in the medial PFC	High	No
			HC (n = 11)	33.6 (7.2) NA									
[133] Zhong et al.	Cross-sectional	NAA/PCr + Cr in ACC	fMDD (n = 61)	25.34 (6.46) 18–45	Current pregnancy or postpartum depression excluded	DSM-5	HDRS: 25.64 (4.07)	None	Treatment-naive	fMDD < HC	↓ NAA/PCr + Cr ratio in right ACC	High	No
			HC (n = 35)	23.60 (3.29) 18–45									
*Positron emission tomography (PET)*													
[134] Nugent et al.	Cross-sectional	rCBF during working memory n-back, fixation and handgrip	fMDD (n = 10)	33 (9.9) NA	Current pregnancy or breastfeeding excluded	DSM-IV	BDI: 26 (13.9)	None	Medication-free for at least 3–8 weeks	fMDD < HC	↓ globally normalized rCBF in DLPFC during all paradigms, and in fusiform gyrus in handgrip task	High	Yes
			HC (n = 7)	31 (9.3) NA									

*FA* fractional anisotropy, *fMDD* female non-peripartum major depressive disorder, *HC* healthy controls, *NA* not available, *DSM* diagnostic and statistical manual of mental disorders, *MADRS* montgomery–åsberg depression rating scale, *ECT* electroconvulsive therapy, *FD* fibre density, *FDC* fibre density cross-section, *AD* axial diffusivity, *HAMD/HDRS* hamilton depression rating scale, *GMV* gray matter volume, *BDI/BDI-II* beck’s depression inventory, *ACC* anterior cingulate cortex, *PFC* prefrontal cortex, *CT* cortical thickness, *SA* surface area. *CV* cortical volume, *GMD* gray matter density*, GMC* gray matter concentration, *ICD-10* international statistical classification of diseases and related health problems 10th revision, *SFG* superior frontal gyrus, *MFG* middle frontal gyrus, *STG* superior temporal gyrus, *FC* functional connectivity, *CAPs* functional network co-activation patterns, *DMN* default-mode network, *FPN* frontoparietal network, *ALFF* amplitude of low-frequency fluctuations, *DC* degree centrality, *ReHo* regional homogeneity, *CCMD-3* chinese classification of mental disorders, *sgACC* subgenual ACC, *SN* salience network, *DLPFC* dorsolateral prefrontal cortex, *OFC* orbitofrontal cortex, *MINI* mini international neuropsychiatric interview, *PCC* posterior cingulate cortex, *GABA* gamma-aminobutyric acid, *Glu/Glx* glutamate and glutamine, *NAA* N-acetyl-aspartate, *tCho* total choline, *Cr* creatine, *NAA/PCr + Cr* NAA/ phosphocreatine + creatine ratio, *rCBF* regional cerebral blood flow.

*values including both male and female participants.

Twelve studies indicated concomitant anxiety disorders/symptoms [42, 45, 50, 57, 58, 67, 69, 70, 72, 73, 75, 78], while the remaining studies did not report any clinical comorbidity. Additionally, several studies reported first episode PPD (history of previous mental disorder excluded, including depression; n = 23; [21, 35–37, 40, 41, 43, 44, 47–49, 51–56, 59–61, 64, 65, 71]), although others included participants with a previous history of non-peripartum MDD (n = 8; [42, 57, 58, 67, 70, 76–78]) and/or previous history of PPD (n = 5; [42, 57, 58, 70, 76]). Regarding treatment status, participants across studies were either treatment-naive, not undergoing treatment at the time of the experiment or medication-free, while in four studies antidepressants or psychotherapy were accepted [66, 72, 73, 78].

For fMDD, studies used DTI/DKI/DWI (*n* = 2), structural MRI (*n* = 12), resting-state (*n* = 15) and task-based fMRI (*n* = 19), NIRS (*n* = 2), MRS (*n* = 9) and PET (*n* = 1). The majority of studies did not provide information on the previous history or current pregnancy status of participants, with 24 studies explicitly excluding pregnant and/or breastfeeding participants [81, 85, 86, 90, 92, 96, 98–103, 105, 108, 113, 120, 122, 124, 126, 129–131, 133, 134]. Eighteen studies included participants in the reproductive stage (18–49 years old; [55, 88, 95, 96, 98, 100, 101, 104, 105, 113, 115, 117, 122, 126, 129–131, 133]), while in the remaining studies the age range surpassed 50 years or was unspecified. All studies used standardized diagnostic criteria to assess MDD, except for one study where the method used is unclear [94].

Of the studies reviewed, most reported no comorbid conditions, while in four anxiety disorders or symptoms were present [91, 93, 95, 108]. Nine studies had either unavailable or unclear data regarding clinical comorbidity [83, 90, 109, 112, 113, 115, 118, 124, 130, 131]. Finally, 29 studies were conducted with participants who were antidepressant/medication free or naive [55, 82, 86, 88, 91–93, 95–97, 99–101, 104, 105, 109, 110, 114, 115, 122–124, 128–134], while in 22 studies participants were using antidepressants, undergoing neuromodulation, or receiving psychotherapy [81, 83, 84, 87, 89, 90, 98, 103, 106–108, 111, 112, 116–121, 125–127]. Four studies had unclear or unavailable data regarding current treatment status [85, 94, 102, 113].

Overall, studies ranged from moderate to high quality (supplementary tables S5–S7). Notably, bias in cross-sectional designs primarily stemmed from a lack of detailed descriptions regarding study participants and design (e.g., failing to specify the postpartum timepoint) and an inadequate identification and control for confounding factors. Concerning cohort studies, a prevalent source of bias centred around the adequacy of follow-up, with instances of follow-up rates falling below 80% or lacking sufficient information.

#### Peripartum depression vs healthy controls

##### Structural correlates

In white matter, PPD has been associated with increased mean diffusivity (MD) in temporo-parietal areas, superior longitudinal fasciculus, corticospinal tract, cingulum, body and splenium of the corpus callosum, external capsule, internal capsule, inferior longitudinal fasciculus, and putamen [41]. Additionally, decreased fractional anisotropy (FA) has been found in the superior longitudinal fasciculus, corticospinal tract, thalamus [41], and in the left anterior limb of the internal capsule [42], along with reduced radial diffusivity (RD) in the cingulum tract [40]. In contrast, increased FA was observed in the right anterior thalamic radiation and cingulum tracts [40].

In grey matter, PPD participants had increased volume (GMV) in the left DLPFC [43, 44], right anterior insula [44] and OFC [43] and reduced GMV in AMY [46]. Significant volumetric differences were also noted in the right ACC and left middle-PCC [45], as well as increased cortical thickness (CT) in the left superior frontal gyrus, cuneus and fusiform gyrus [49], but decreased CT in the right inferior parietal lobule [47]. Additionally, increased surface area was observed in the left superior frontal gyrus, caudal middle frontal gyrus (MFG), middle temporal gyrus (MTG) and insula, along with increased mean curvature in the parietal lobules [47].

Four studies [42–44, 47] on 136 participants were included in the structural meta-analysis and no significant clusters were identified.

##### Functional correlates

In the PFC, increased resting-state connectivity was observed between the left DLPFC and right ACC [43], while connectivity was decreased between the dorsomedial PFC and left ventral striatum [35], precuneus and PCC [58] and between the DLPFC, ACC and AMY [57]. Decreased sample entropy was found in the left medial PFC [37], as well as reduced regional homogeneity (ReHo) in left DLPFC [53, 59] and decreased voxel-mirrored homotopic connectivity (VMHC) in bilateral dorsomedial PFC [65]. Increased values in amplitude of low-frequency fluctuations (ALFF) were found in left medial PFC and DLPFC [54]. For the OFC, increased connectivity was observed with the right MFG and left inferior occipital gyrus [43], as well as decreased ALFF [54] and VMHC [65].

Regarding the ACC, its subgenual part (sgACC) showed increased connectivity with the ventral anterior insula [35, 36] and decreased connectivity with the superior and MTG [36]. Within the right hippocampus, degree centrality and ReHo were increased [59, 64], as well as connectivity with the left precuneus and left superior frontal gyrus [59], while connectivity was reduced with the right MFG and left median cingulate and paracingulate gyri [64]. The PCC showed reduced connectivity with the right AMY [50] and with the right paracentral lobule [52] and increased ReHo [61]. Finally, ReHo and VHMC reductions were found in the right insula [53, 54, 59, 63] and AMY [53, 63].

**Fig. 1:**
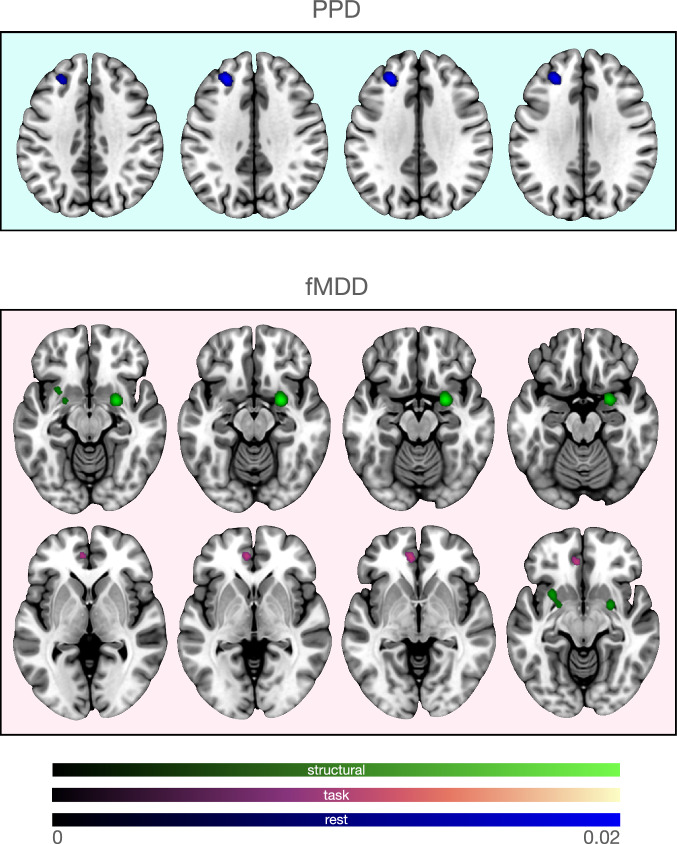
Results of the imaging (structural, resting-state and task-based fMRI) ALE meta-analyses showing clusters with significant ALE maxima in PPD and fMDD patients. While results for MFG in PPD seem to have been driven by resting state data (upper panel), for fMDD subcortical results in amygdala and putamina were driven by structural and VLMT by functional investigations (lower panel).

Eighteen studies were included in the resting-state meta-analysis (16 experiments; [35–37, 43, 50, 51, 53–59, 61–65]; 367 participants), which highlighted abnormalities in the left MFG (Fig. 1; Table S8).

Studies using infant stimuli in fMRI report differential emotional processing in PPD. Specifically, Dudin et al. [66] found an increased right AMY response to unfamiliar smiling infants, while Wonch et al. [72] observed a general increase in BOLD response in the right AMY. The latter also reported decreased bilateral AMY-right insular cortex connectivity when participants viewed faces of their own versus other infants. Lenzi et al. [68] found increased deactivation in the orbital and medial PFC and an increase in right AMY reactivity. Finnegan et al. [67] observed a differential response to infant versus non-infant stimuli in brain regions such as the right dorsolateral superior, middle, and inferior frontal gyri, the left inferior and middle temporal lobe, and bilateral angular gyri. Interestingly, the authors found that a history of depressive episodes did not independently impact these neural responses.

For non-infant stimuli, Moses-Kolko et al. [69] observed increased nonlinear attenuation of left ventral striatal activity after reward in PPD. In response to negative stimuli, decreased activation was observed in bilateral OFC, cingulate, putamen, precuneus, DLPFC, ACC [21], right AMY [21, 71], left AMY and left dorsomedial PFC [70], alongside increased activity in the bilateral insula [21]. For positive stimuli, decreased activity was found in striatum, cingulate gyrus, DLPFC and precentral gyrus [21]. Using fNIRS, increased depression severity was found to be associated with decreased connectivity between the temporoparietal junction (TPJ) with lateral PFC and increased connectivity between TPJ with anterior medial PFC [73]. In contrast, Song et al. [74] found no differences in integral or centroid values.

Six studies were included in the task-based meta-analysis [21, 68–72]; 67 participants) and no significant clusters were found.

##### Metabolic correlates

An increase in monoamine oxidase A in the PFC and ACC was found in PPD [79] but no differences in D2/3 receptor binding potential [80]. MRS studies identified a decrease in glutamate-glutamine (Glx) and N-acetylaspartate (NAA) levels in the left DLPFC [78]. Additionally, McEwen et al. [77] found increased glutamate (Glu) levels in the medial PFC in PPD, though other metabolite levels (NAA, creatine [Cr] and choline [Cho]) did not show significant differences. There was also a trend towards decreased cortical gamma-aminobutyric acid (GABA) levels in PPD [76], although Deligiannidis et al. [58] found no significant differences in GABA/Cr concentrations in the pregenual ACC or occipital cortex.

##### Multimodal meta-analysis of PPD correlates

**Fig. 2:**
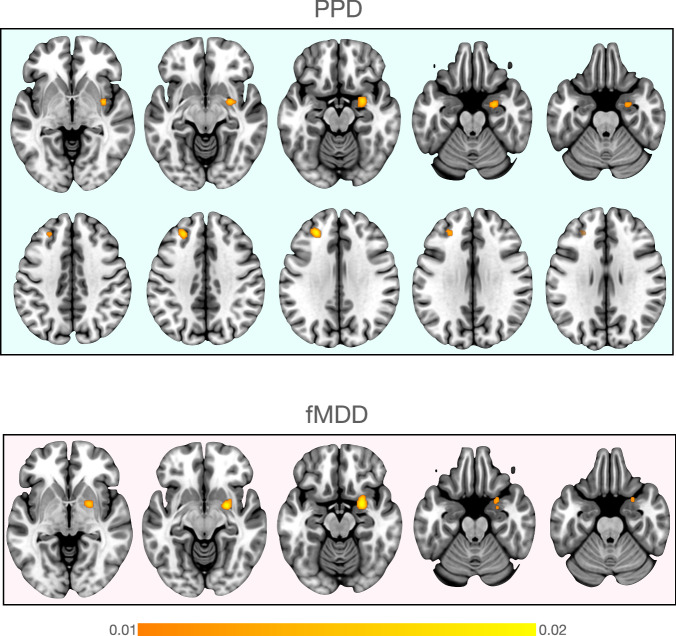
Results of the multimodal ALE meta-analyses showing clusters with significant ALE maxima for PPD and fMDD. While multimodal analysis for PPD revealed significant clusters in the right putamen, amygdala and left MFG, in fMDD there were only subcortical clusters in the right putamen and amygdala.

In the pooled meta-analysis of all included studies (25 experiments, 542 participants), women diagnosed with PPD exhibited structural and functional changes in right putamen, right amygdala and left MFG (Fig. 2 and Table S8). Additional exploratory direction of effect analyses results are provided in supplementary Table S9 and Fig. S6.

##### Network effects

**Fig. 3:**
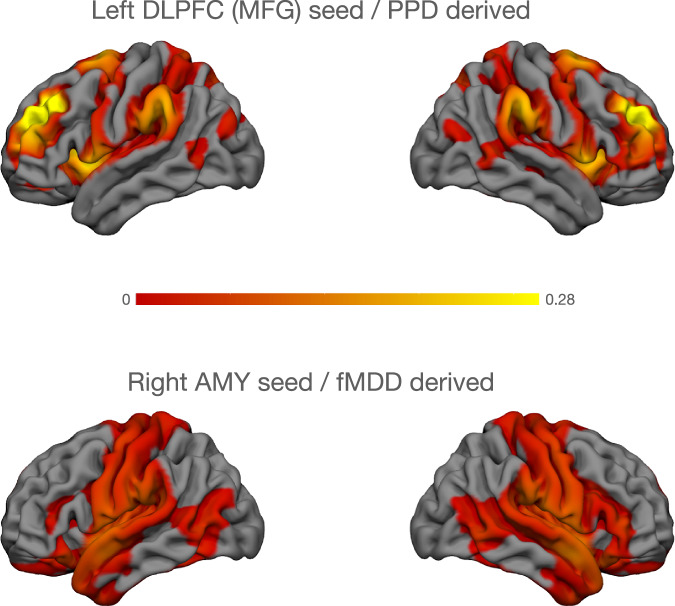
Results of seed-based connectivity analysis with the left DLPFC (MFG) as a seed in PPD and right amygdala as a seed in fMDD. While the PPD derived network was marked by involvement of bilateral DLPFC and angular gyrus (upper panel), fMDD derived network was marked by involvement of somatosensory, motor and anterior temporal cortices. Connectivity based on neurosynth.

In the seed-based connectivity analysis using the left DLPFC (MFG) as the seed region, significant positive connectivity was observed across several cortical areas (Fig. 3). Regions with the strongest connectivity (yellow) include areas of the bilateral DLPFC and angular gyrus. Additional activation is seen in adjacent prefrontal regions, as well as posterior parietal areas. Areas with lower but still significant connectivity (red) extend into occipital and temporal cortices.

#### Female non-peripartum major depression vs healthy controls

##### Structural correlates

In white matter, decreased fibre-density was observed in the left and right frontal projections of the corpus callosum, right anterior limb of the internal capsule, tapetum, and right inferior longitudinal fasciculus [82]. Additionally, reductions in FA were widespread in the genu of the corpus callosum, bilateral cerebral peduncles, forceps minor and major, bilateral inferior fronto-occipital fasciculus [82], left bilateral uncinate fasciculi [81, 82], inferior and superior longitudinal fasciculi [81].

For gray matter, reduced GMV was found in bilateral ventral ACC [91], right AMY [91, 93], bilateral caudate extending into the anterior nucleus of the thalamus [87], medial PFC [84], left lingual gyrus extending to the parahippocampal gyrus, cerebellum [92, 93], bilateral insula, bilateral putamen, and caudal middle-frontal region [93]. However, another study did not find GMV differences within the AMY, hippocampus, sgACC or putamen [87]. Reductions in AMY volume were observed across studies [85, 90].

Nine studies were included in the structural meta-analysis ([82, 84, 86–89, 91–93]; 251 participants). Women diagnosed with MDD manifested structural alterations in left putamen gray matter and bilateral sub-lobar extra-nuclear white matter (Table S10; Fig. 1).

##### Functional correlates

Decreased connectivity was observed between: the right AMY and the ventrolateral PFC, bilateral insula, and bilateral putamen [93]; the left hippocampus and temporo-occipital regions, including the bilateral lingual gyrus and fusiform [103]; and the left middle occipital gyrus and the left OFC [104]. Increased connectivity was found between the left middle occipital gyrus and the left medial prefrontal gyrus and the left hippocampus [104] and between the left MFG and bilateral putamen [96]. ALFF reductions were reported in the right putamen, right MTG [106], left middle occipital gyrus [104], right postcentral gyrus [96] and right superior occipital gyrus [105], while increases were observed in the left medial PFC [106], left MFG [102, 106], left precentral gyrus [102] and left temporal pole [55]. There were no observed differences in fractional ALFF in the ACC and insula [98]. ReHo was elevated in the left sgACC and left thalamus [55]. Conversely, BOLD signal variability was reduced in bilateral cerebellum [100] and the DLPFC [101]. Nine studies were included in the resting-state meta-analysis ([55, 84, 96, 100, 102–106], 192 participants). No significant clusters were found.

The ACC showed increased activation during the presentation of positive stimuli [107, 119], emotional approach and withdrawal conditions [109], incongruent conditions [123] and rejection [125], while reduced activity was observed in response to negative stimuli [116]. Connectivity of the ACC was reduced with the AMY during negative stimuli and with the DLPFC during high-attention stimuli [120]. Different patterns emerged in response to positive and fearful stimuli, with increased inverse connectivity between the left-sided sgACC and AMY to happy faces, and increased positive connectivity between the same regions to fearful faces [108].

The DLPFC also showed increased activation during expectation of negative stimuli [107], incongruent versus congruent contrasts [123] and painful stimuli [110]. In contrast, decreased activation was found during low-risk cheating choices [121] and decreased connectivity with the right AMY during high-attention stimuli [120]. Reduced activity was also found in: the dorsal putamen and anterior insula during low-risk cheating choices [121] and in frontoparietal network and salience networks, irrespective of stress [115]; in the right caudate during the recall of positive specific memories. On the other hand, increased activation was noted in the PCC, insula, and thalamus during the recall of negative specific memories [124]. Using fNIRS, a significant correlation between depression scores and changes in oxy-Hb in the right frontal brain region was observed [126], as well as reduced oxy-Hb activation in the DLPFC [127].

Fourteen studies were included in the task-based meta-analysis ([107, 110–112, 116–125]; 225 participants). Women diagnosed with MDD manifested alterations in the left ACC (Fig. 1; Table S10).

##### Metabolic correlates

In the medial PFC, there were no significant differences in Glu, GABA, or Glx levels [106, 130]. Similarly, Glu levels in the left DLPFC were not significantly different, although there was a reduction in GABA+ levels and in the GABA+ to Glu ratio in this region [131]. In the ACC, there was a reduction in GABA levels [115] and in NAA to phosphocreatine plus creatine (NAA/PCr+Cr) ratio [133]. In the ventral prefrontal white matter, reduced Cho/Cr ratios were observed bilaterally, while NAA/Cr levels showed no significant differences [132]. In the hippocampus, there were increased total Cho levels [103] and decreased taurine concentration [129]. The only PET imaging study revealed a global reduction in regionally normalized cerebral blood flow in the DLPFC [134].

##### Multimodal meta-analysis of fMDD neural correlates

In the pooled meta-analysis of all included studies (32 experiments, 652 participants, [107, 110–112, 116–125]), women diagnosed with MDD exhibited changes in right putamen and amygdala (Fig. 2). Direction of effect analyses are provided in supplementary Table S11 and Fig. S7. In order to investigate if there was a potential effect of age in the fMDD sample, we calculated an exploratory subgroup analysis for fMDD studies that was age-matched to PPD samples. The general pattern of ALE results was maintained in this age-matched exploratory analysis (supplementary Fig. S8).

##### Network effects

The PPD derived network (Fig. 3) was marked by involvement of bilateral DLPFC and angular gyrus.

The fMDD derived right amygdala seed region (Fig. 3), showed connectivity increase in the somatosensory and motor cortices and anterior temporal lobes. Further connectivity changes extend into the posterior temporal cortices and insulae.

#### Peripartum depression vs non-peripartum female major depression

##### Structural correlates

Both PPD and fMDD show widespread reductions in FA in several tracts, including the superior longitudinal fasciculus [41, 81]. PPD is associated with increased GMV in the right insula and right precentral gyrus [43, 44], while MDD exhibits reduced GMV in the same areas [89, 93]. Both conditions show reduced AMY volumes [46, 91, 93].

##### Functional correlates

PDD and fMDD present both shared and distinct patterns of altered brain function. Shared findings include increased ALFF in left medial PFC [54, 106], increased ReHo in thalamus [55] and decreased activity in the ACC and cingulate in response to negative stimuli [21, 116]. Additionally, there is reduced activity in the cingulate gyrus in response to positive stimuli [21, 116], and reduced AMY-insula connectivity [72, 93]. However, PPD is characterized by decreased activity in AMY and precuneus, along with increased activity in the insula, in response to negative stimuli [21, 71]. Conversely, fMDD has increased activity in the AMY and precuneus and decreased activity in the insula [107, 116].

One study directly compared PPD, fMDD and HC to identity shared and different resting-state neural circuits [55]. Both PPD and fMDD groups showed higher fALFF in the left temporal pole (vs HC). The fMDD group showed a specifically increased FC in the right cerebellum, whereas PPD had specifically decreased fALFF in the left supplementary motor area and the posterior MTG, and reduced posterior MTG-precuneus and left-right sgACC connectivity. Additionally, there were significant ReHo differences in the left thalamus and left sgACC (PPD > fMDD > HC).

##### Metabolic correlates

In the medial PFC, PPD shows increased Glu levels [77]. In fMDD, however, there were no significant differences in Glu, GABA, or Glx concentrations in this region [106, 130].

##### Conjunction and contrast analyses

**Fig. 4:**
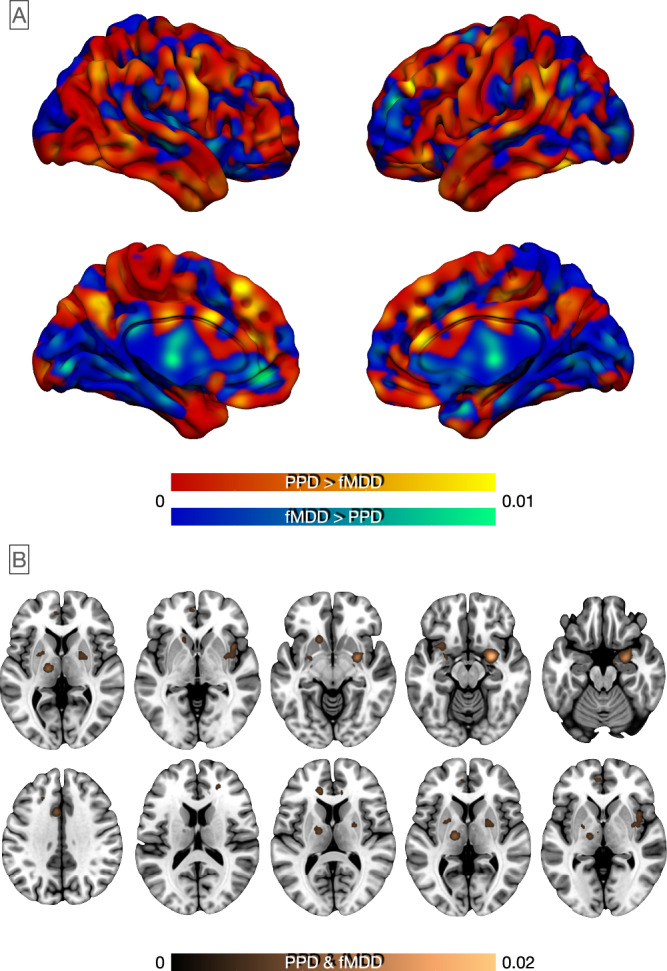
Results of conjunction ALE meta-analysis in PPD and fMDD patients. **A** In PPD there were more prominent alterations in big parts of the temporal lobes and somatosensory cortices as compared to fMDD (warm colors), while in fMDD there was stronger involvement of DLPFC and ACC (cool colors). **B** There was a significant overlap between PPD and fMDD in the right insula, left ventral lateral nucleus (thalamus), left caudate, right amygdala, left cingulate gyrus and bilateral putamina.

The conjunction analysis (57 experiments, 1194 participants) identified regions in the right insula, left ventral lateral nucleus (thalamus), left caudate, right amygdala, left cingulate gyrus and bilateral putamina demonstrating convergent brain changes in both PPD and fMDD (Table S12; Fig. 4). Contrast analyses revealed that PPD was associated with more prominent alterations in temporal lobes and somatosensory cortices, while fMDD showed stronger involvement of the DLPFC and ACC (Fig. 4).

##### Network effects

Dice coefficients for PPD and fMDD were low for both symptom networks: 0.0858 (PPD/anxiosomatic), 0.0924 (PPD/dysphoric), 0.0845 (fMDD/anxiosomatic) and 0.0995 (fMDD/dysphoric).

##### Specificity of results

The dummy dataset of randomly assigned coordinates did not result in any significant cluster accounting for strict thresholding. This suggests that our meta-analytical results on PPD are not merely the consequence of compiling heterogeneous study results for analysis.

## Discussion

This systematic review and meta-analysis aimed at exploring the neural correlates of PPD and compares them with non-peripartum fMDD. We discuss our findings within the theoretical framework of network biotypes proposed by Leanne Williams’ team [135–137], which include the DMN, salience (SN), frontoparietal attention (AN), negative affect (NA), positive affect (PA) and CCN. These biotypes have been shown to be psychometrically reliable and were recently validated in patients with depression and anxiety [138].

### Neural alterations in PPD

Our comprehensive analysis identified structural, functional, and metabolic alterations within the SN, NA, DMN, and CCN networks in women experiencing PPD, compared to HC. The meta-analysis pinpointed changes in the left MFG/DLPFC and right putamen and AMY, while the qualitative synthesis additionally highlighted alterations in the ACC, insula, medial PFC, OFC, putamen, thalamus, hippocampus, PCC, precuneus, MTG, superior frontal, fusiform, cingulate, precentral and angular gyri. These alterations in networks governing emotional and cognitive processes may contribute to the diverse manifestation of symptoms and behaviors in PPD.

The SN (encompassing the ACC, the anterior insula and the temporal pole) plays a crucial role in detecting salient internal sensations and external changes, guiding cognitive processing/control and coordinating behavioral responses essential for threat detection [139], particularly relevant in motherhood [11]. Disruptions in this circuit may contribute to anxious arousal and avoidance behaviors (i.e., difficulties in discerning relevant cues and avoidance of overwhelming situations; [135, 136]) and biased affective processing. In PPD, reduced ACC activity and increased insula activity in response to negative stimuli was found, as well as sgACC-insula hyperconnectivity and ACC-AMY hypoconnectivity. These disruptions can manifest as symptoms commonly observed in PPD, including anxiety, somatic complaints (e.g., fatigability, sleep disturbances; [140]) and psychomotor symptoms (agitation/restlessness; [17, 141]).

The CCN (involving the DLPFC, the dorsal ACC and the dorsal parietal cortex) is crucial for higher executive functions, namely working memory, selective attention, and cognitive flexibility [135, 136]. Due to its heightened connectivity with networks involved in emotion and reward, the CCN may have down-stream effects on affective circuits [142, 143]. Reduced activity in response to both negative and positive stimuli in DLPFC was found in PPD patients, alongside decreased Glx and NAA levels and increased GMV. Additionally, the left DLPFC had a strong connectivity with areas of the frontoparietal network and occipital and temporal cortices. Dysfunction in the CCN has been associated with heightened anxiety anticipation [15] and inattention/cognitive dyscontrol (poor concentration, difficulty paying attention, indecisiveness; [25]), as well as maladaptive emotion regulation (e.g., difficulties in suppressing negative emotions). In depressed postpartum women, impaired concentration/decision-making are prominent symptoms [17]. Also, studies have shown that emotion regulation difficulties are associated with depressive and anxiety symptoms during pregnancy and across the postpartum period [144, 145]. Finally, impairments in cognitive control, including repetitive negative thinking and worry, are particularly relevant for the postpartum period due to the executive function demands of parenting, which include planning, attention and working memory abilities [146].

Recent findings of cognitive biotypes of MDD (with impairments in executive function and response inhibition, insomnia, and poor psychosocial function; [147]) and cognitive mechanisms of postpartum depression [148] may indicate that cognitive control dysfunction underlies depression in general [146] and constitutes a transdiagnostic factor, as many psychiatric disorders are associated with deficits in cognitive control [149]. However, negative repetitive thinking in peripartum women with depressive symptoms tends to focus on peripartum-specific concerns within self, motherhood, and interpersonal domains, such as unmet high expectations, thoughts about harming the infant and parenting efficacy, which highlights the existence of factors unique to the peripartum period [17, 150]. Dysfunction in the SN and CCN can also contribute to negative interpersonal and attentional biases [15]. Women with PPD exhibit a negative bias perception of ambiguous and distressful infant stimuli, which has been associated with an increased vulnerability for PPD [151] and may impair mothers’ evaluations of their parenting and ability to detect and respond to their infants’ needs, thereby affecting maternal sensitivity [152].

The DMN (medial PFC, PCC, angular gyrus, TPJ) is a resting-state network involved in self-referential processing, emotion regulation, mentalizing and metacognitive processing of psychological states, which has been associated with self-criticism and rumination [153]. In PPD, a consistent pattern of attenuated resting-state connectivity within the DMN has been found, particularly between the PCC-AMY, TPJ-anterior medial PFC and the dorsomedial PFC, precuneus, PCC and the angular gyrus. Reduced medial PFC activity in response to negative stimuli and increased Glu levels has also been observed. This DMN dysfunction might contribute to negative self-perceptions, feeling overwhelmed, excessive worry about parenting abilities, and difficulty disengaging from negative thoughts, leading to increased feelings of guilt in PPD [17]. Additionally, altered DMN function may impact how attentive the mother is to her newborn, potentially contributing to the bonding deficits commonly observed in PPD [50].

### Comparative analysis with non-peripartum female MDD

Our comparison between women diagnosed with PPD and non-peripartum women diagnosed with MDD revealed shared alterations in regions of the DMN (e.g., increased ALFF in medial PFC), SN (e.g., decreased activity in ACC to negative stimuli, higher fALFF in left temporal pole) and NA (e.g., AMY-insula hypoconnectivity). These altered correlates suggest a shared dysfunction in emotion processing, threat sensitivity and mood regulation in female depression subtypes.

However, distinct structural and functional patterns emerged across networks (SN, NA and AN; qualitative comparison). In PPD, there was increased volume and activity in the right insula in response to negative stimuli, as well as decreased activity in AMY and precuneus. In contrast, fMDD was associated with reduced GMV and decreased activity in the insula, alongside increased activity in AMY and precuneus in response to negative stimuli. Additionally, a study directly comparing PPD and fMDD [55] discovered that fMDD had increased DC in the right cerebellum, whereas PPD showed decreased fALFF in the left supplementary motor area and the posterior MTG, and reduced MTG-precuneus and left-right sgACC connectivity. These differences extend to the metabolic level, where, in the medial PFC, women with PPD show increased Glu levels, while no significant differences were present in fMDD. Finally, exploratory analyses suggested low correspondence between PPD and fMDD ALE maps and common symptom networks [39]. This could be due to distinctive underlying mechanisms, symptom interactions, or network connectivity for PPD and fMDD, in the anxiosomatic and the dysphoric domains. Further systematic research is needed to unravel the neurosymptomatic interactions in PPD and fMDD.

We further observed reduced AMY activation in response to negative stimuli in PPD and an increased response to positive infant stimuli. This is in contrast with findings in non-peripartum MDD literature, where patients commonly exhibit amygdala hypoactivity in response to positive stimuli and heightened amygdala activation when exposed to negative stimuli. Notably, O’Brien and colleagues [154] compared women with a history of postpartum depression with women with a history of non-postpartum MDD during the late luteal phase of the menstrual cycle. Their findings revealed hypoactivity in response to positive emotional faces in the right amygdala in women with previous PPD. These inconsistent neural profiles of hyper and hypo-amygdalar activity support the hypothesis of different biotypes of neural circuit dysfunction [136] and that women with and at risk for PPD may constitute a unique subgroup with divergent sensitivity to hormonal influences [155].

Furthermore, research has shown that PPD is also a heterogeneous disorder comprised of different clinical subtypes (e.g., [155, 156]), based on timing of onset, duration and severity, which can be distinguished considering biological, psychological and social factors [17]. For example, Fox and colleagues [156] found six different symptom clusters of postpartum depression, namely worry (e.g., anxiety and guilt), anger, emotional/circadian/energetic dysregulation (e.g., agitation, fatigue, sadness), appetite, somatic/cognitive (e.g., inability to focus) and distress display (e.g., crying, sad affect display), which may reflect the different neural networks involved (e.g., SN, NA, and CCN).

In summary, while there are shared neural mechanisms underlying depressive disorders, the peripartum period may introduce distinct neurobiological changes that contribute to a specific manifestation of depression. The influence of hormonal fluctuations, reproductive-related neuroadaptations, and the socio-environmental context during the peripartum period may contribute to the observed differences [20]. However, several confounding factors may influence the neural correlates observed in PPD and its comparison with fMDD. Firstly, PPD generally affects women within a narrow reproductive age range (18–49 years), whereas fMDD spans a broader age range (from young adulthood to later life) and contexts (not exclusively related to the peripartum period). These variations in age can introduce significant differences in brain structure, hormones, and life circumstances, which may hinder the interpretation of neural and clinical findings. Research comparing PPD and fMDD in women aged 21–42 identified both overlapping and distinct resting-state neural circuits [55]. Our ALE results were also maintained when selecting a subgroup of age-matched fMDD studies. Secondly, previous history of depressive episodes (a strong psychological risk factor for PPD; 5, [17]) may lead to long-term changes in neural circuits. In our review, only 51% of studies focused on first-episode PPD, leaving potential prior episodes unaccounted for.

### Clinical relevance

Recognizing differences in symptom presentation and neural correlates between PPD and non-peripartum MDD can help improve the identification of PPD cases [109]. Considering the unique psychosocial and physiological changes, alongside altered brain function across the peripartum period, our findings are in line with the need for the extension of the onset specifier to one year postpartum [17].

Treatment for PPD usually follows standard MDD guidelines and is based on pharmacotherapy or psychotherapy (e.g., [157]) to reduce symptoms, improve quality of life and general functioning [20]. Although serotonin reuptake inhibitors (SSRIs) are among the first-line treatments for PPD [158], pregnant or breastfeeding women often present concerns regarding side effects and potential effects on fetal and infant development [159]. As a result, treatment may be refused, or doses may be reduced below what is clinically advised [160]. To achieve widespread access to high-quality peripartum mental health care, new solutions are therefore required.

Brain circuit organization and function have emerged as both an explanatory model and a foundation for designing interventions, aiming to address major circuits and neurotransmitter pathways disrupted in psychiatric disorders [161]. The evolving approach of targeting specific brain circuits associated with distinct symptom clusters offers a promising avenue for more personalized treatment strategies [26, 161]. Tailoring therapeutic approaches based on neural signatures may enhance the effectiveness of treatments for both MDD and PPD and, with increasing evidence, may guide intervention choice. For example, non-invasive brain stimulation techniques (NIBS) have been proposed as alternatives to traditional therapies for PPD, with meta-analyses hinting towards the effectiveness, safety and acceptability of repetitive transcranial magnetic stimulation (rTMS) treatment for PPD [162–164]. Understanding the disrupted neural networks involved in PPD will allow for personalized interventions (e.g., increased precision in defining the target areas for stimulation).

Additionally, while SSRIs and other treatment approaches may impact biological transdiagnostic factors, the unique physiological changes of the peripartum period calls for more tailored treatments, such as addressing GABAergic dysfunction [20]. Evidence also suggests that distinct activation patterns in AMY-PFC can predict treatment responses, especially to antidepressants [137]. Thus, neuroimaging measures hold promise for guiding the selection of the most effective treatment for different psychiatric biotypes.

### Future directions and limitations

In conclusion, this systematic review and meta-analysis provides a comprehensive overview of the neural correlates of PPD, offering insights into both shared and distinct features compared to non-peripartum fMDD. The identified alterations in brain regions associated with emotion processing and cognitive functions emphasize the need for targeted interventions in the management of PPD. Future research, guided by larger, well-controlled longitudinal studies, is crucial for advancing our understanding of the neurobiological underpinnings of PPD and informing innovative treatment approaches.

Despite the contributions of this study, some limitations must be acknowledged. The majority of studies focused exclusively on the postpartum period, leaving a gap in our understanding of the antenatal neural changes associated with PPD. Emerging evidence highlights differences between pre- and postpartum symptom networks, suggesting that antenatal and postpartum maternal mood and anxiety may have different presentations. Additionally, this study does not quantitatively compare PPD and fMDD, but instead performs indirect comparisons through their respective differences with HC or correlation with depression severity. In relation to MDD, although we attempted to minimize the effects of sex by considering female only participants, studies did not report on previous history or current pregnancy, which may impact the comparison results. Moreover, bias in studies, including insufficient participant descriptions and confounding factor control, poses challenges to the generalizability of findings. Analyses split for different modalities suggest that cortical effects in ALE are largely driven by resting-state results, which might be explained by the higher proportion of these studies. For fMDD some results were stronger driven by task-based fMRI or structural imaging. Although this might be a limitation to interpretability, we would like to stress that some clusters in the multimodal analysis only emerged via merging cross-modal data. The exclusion of other non-English language studies (e.g., Chinese) may impact the comprehensiveness of the review. Although we conducted an additional exploratory search for studies in Portuguese, Spanish, German, French, Dutch, and Greek in the PubMed database, this search did not yield any additional articles that met our inclusion criteria.

We highlight the need for future studies in PPD to adequately characterize participants (e.g., postpartum timepoint, parity, previous history of depression) and data acquisition parameters. It would be further interesting to compare different pre-processing protocols (quality control, pre-processing steps, correction for multiple comparisons) and assess the validity and replicability of implemented paradigms. Additionally, future synthesis may consider further specificity testing (i.e., how the neural correlates of PPD differ from those of other brain disorders associated with pregnancy and childbirth) and individual profiles of participants, through individual participant data meta-analysis (IPD; [165]). Finally, exploring antenatal neural changes, investigating the influence of hormonal fluctuations, and considering socio-environmental factors will further enrich our understanding of PPD, as well as studies directly comparing PPD and fMDD while considering confounding variables such as reproductive age, psychosocial stress factors and peripartum timepoints.

## Supplementary information

PRISMA 2020 Checklist

Supplementary Material
