# Supplementary material for: Neural correlates of peripartum depression: a systematic review, meta-analysis and comparison to major depressive disorder
Source: Mol Psychiatry. 2025 Sep 8;30(12):5979–6006. doi: 10.1038/s41380-025-03227-2 (PMC12602369; doi:10.1038/s41380-025-03227-2)
Supplement: Supplementary file 2 — Supplementary Material [file 41380_2025_3227_MOESM2_ESM.docx]

**SUPPLEMENTARY INFORMATION**

Neural correlates of peripartum depression: a systematic review, meta-analysis and comparison to major depressive disorder

Table S1. PRISMA Checklist

Table S2. Complete search strategy

Figure S1. Flow diagram of selection process.

Table S3. Comprehensive MDD meta-analysis

Figure S2. Results of ALE meta-analysis showing clusters with significant ALE maxima in MDD patients, superimposed on an MNI-normalised template.

Table S4. Comparison of PPD and fMDD studies

Figure S3. Imaging modalities in PPD and fMDD

Figure S4. Sociodemographic and clinical characteristics in PPD studies

Figure S5. Sociodemographic and clinical characteristics in fMDD studies

Table S5. Summary of risk of bias assessment for cross-sectional PPD studies

Table S6. Summary of risk of bias assessment for cohort PPD studies

Table S7. Summary of risk of bias assessment for cross-sectional fMDD studies

Table S8. Results of the ALE meta-analyses on PPD participants, in comparison to HC

Table S9 and Figure S6. Direction of effect analyses in PPD

Table S10. Results of the ALE meta-analyses on fMDD participants, in comparison to HC

Table S11 and Figure S7. Direction of effect analyses in fMDD

Table S12. Results of the conjunction ALE meta-analysis between PPD and fMDD

Figure S8. Results of the ALE meta-analysis with an age-matched subgroup of fMDD studies

**Table S1. PRISMA Checklist**

| **Section and Topic** | **Item #** | **Checklist item** | **Location where item is reported** |
| --- | --- | --- | --- |
| **TITLE** | | |  |
| Title | 1 | Identify the report as a systematic review. | Title page |
| **ABSTRACT** | | |  |
| Abstract | 2 | See the PRISMA 2020 for Abstracts checklist. | pp. 2, lines 28-44 |
| **INTRODUCTION** | | |  |
| Rationale | 3 | Describe the rationale for the review in the context of existing knowledge. | pp. 3-5, lines 53-114 |
| Objectives | 4 | Provide an explicit statement of the objective(s) or question(s) the review addresses. | pp. 5, lines 106-114 |
| **METHODS** | | |  |
| Eligibility criteria | 5 | Specify the inclusion and exclusion criteria for the review and how studies were grouped for the syntheses. | pp. 5-6, lines 121-139 |
| Information sources | 6 | Specify all databases, registers, websites, organisations, reference lists and other sources searched or consulted to identify studies. Specify the date when each source was last searched or consulted. | pp. 6, lines 141-143 |
| Search strategy | 7 | Present the full search strategies for all databases, registers and websites, including any filters and limits used. | pp. 6, lines 143-1149 and suppl. table S2 |
| Selection process | 8 | Specify the methods used to decide whether a study met the inclusion criteria of the review, including how many reviewers screened each record and each report retrieved, whether they worked independently, and if applicable, details of automation tools used in the process. | pp. 6-7, lines 153-160 |
| Data collection process | 9 | Specify the methods used to collect data from reports, including how many reviewers collected data from each report, whether they worked independently, any processes for obtaining or confirming data from study investigators, and if applicable, details of automation tools used in the process. | pp. 6-7, lines 153-160 |
| Data items | 10a | List and define all outcomes for which data were sought. Specify whether all results that were compatible with each outcome domain in each study were sought (e.g. for all measures, time points, analyses), and if not, the methods used to decide which results to collect. | pp. 7 lines 161-166 |
|  | 10b | List and define all other variables for which data were sought (e.g. participant and intervention characteristics, funding sources). Describe any assumptions made about any missing or unclear information. | pp. 7, lines 161-166 |
| Study risk of bias assessment | 11 | Specify the methods used to assess risk of bias in the included studies, including details of the tool(s) used, how many reviewers assessed each study and whether they worked independently, and if applicable, details of automation tools used in the process. | pp. 7, lines 167-177 |
| Effect measures | 12 | Specify for each outcome the effect measure(s) (e.g. risk ratio, mean difference) used in the synthesis or presentation of results. | NA |
| Synthesis methods | 13a | Describe the processes used to decide which studies were eligible for each synthesis (e.g. tabulating the study intervention characteristics and comparing against the planned groups for each synthesis (item #5)). | pp. 8-9, lines 178-216 |
|  | 13b | Describe any methods required to prepare the data for presentation or synthesis, such as handling of missing summary statistics, or data conversions. |  |
|  | 13c | Describe any methods used to tabulate or visually display results of individual studies and syntheses. |  |
|  | 13d | Describe any methods used to synthesize results and provide a rationale for the choice(s). If meta-analysis was performed, describe the model(s), method(s) to identify the presence and extent of statistical heterogeneity, and software package(s) used. |  |
|  | 13e | Describe any methods used to explore possible causes of heterogeneity among study results (e.g. subgroup analysis, meta-regression). | Page 9, lines 210-211 |
|  | 13f | Describe any sensitivity analyses conducted to assess robustness of the synthesized results. | NA |
| Reporting bias assessment | 14 | Describe any methods used to assess risk of bias due to missing results in a synthesis (arising from reporting biases). | NA |
| Certainty assessment | 15 | Describe any methods used to assess certainty (or confidence) in the body of evidence for an outcome. | NA |
| **RESULTS** | | |  |
| Study selection | 16a | Describe the results of the search and selection process, from the number of records identified in the search to the number of studies included in the review, ideally using a flow diagram. | pp. 9, lines 219-225, supplementary Figure S1 |
|  | 16b | Cite studies that might appear to meet the inclusion criteria, but which were excluded, and explain why they were excluded. | NA |
| Study characteristics | 17 | Cite each included study and present its characteristics. | Tables 1 and 2 |
| Risk of bias in studies | 18 | Present assessments of risk of bias for each included study. | Tables S5-S7 |
| Results of individual studies | 19 | For all outcomes, present, for each study: (a) summary statistics for each group (where appropriate) and (b) an effect estimate and its precision (e.g. confidence/credible interval), ideally using structured tables or plots. | Tables 1 and 2 |
| Results of syntheses | 20a | For each synthesis, briefly summarise the characteristics and risk of bias among contributing studies. | pp. 9-11, lines 226-273 |
|  | 20b | Present results of all statistical syntheses conducted. If meta-analysis was done, present for each the summary estimate and its precision (e.g. confidence/credible interval) and measures of statistical heterogeneity. If comparing groups, describe the direction of the effect. | pp. 11-20, lines 274-475 |
|  | 20c | Present results of all investigations of possible causes of heterogeneity among study results. | NA |
|  | 20d | Present results of all sensitivity analyses conducted to assess the robustness of the synthesized results. | NA |
| Reporting biases | 21 | Present assessments of risk of bias due to missing results (arising from reporting biases) for each synthesis assessed. | NA |
| Certainty of evidence | 22 | Present assessments of certainty (or confidence) in the body of evidence for each outcome assessed. | NA |
| **DISCUSSION** | | |  |
| Discussion | 23a | Provide a general interpretation of the results in the context of other evidence. | pp. 20-26, lines 476-641 |
|  | 23b | Discuss any limitations of the evidence included in the review. | pp. 27, lines 650-669 |
|  | 23c | Discuss any limitations of the review processes used. | pp. 27, lines 650-669 |
|  | 23d | Discuss implications of the results for practice, policy, and future research. | pp. 25-26, lines 606-641  pp. 28, lines 670-683 |
| **OTHER INFORMATION** | | |  |
| Registration and protocol | 24a | Provide registration information for the review, including register name and registration number, or state that the review was not registered. | pp. 5, lines 116-120 |
|  | 24b | Indicate where the review protocol can be accessed, or state that a protocol was not prepared. | pp. 5, lines 116-120 |
|  | 24c | Describe and explain any amendments to information provided at registration or in the protocol. | NA |
| Support | 25 | Describe sources of financial or non-financial support for the review, and the role of the funders or sponsors in the review. | pp. 28, lines 684-690 |
| Competing interests | 26 | Declare any competing interests of review authors. | pp. 29, lines 702-703 |
| Availability of data, code and other materials | 27 | Report which of the following are publicly available and where they can be found: template data collection forms; data extracted from included studies; data used for all analyses; analytic code; any other materials used in the review. | NA |

**Table S2. Complete search strategy**

| **First Search**  **24/09/2021** | | |
| --- | --- | --- |
| **Search 1** | **PPD** | |
| **Database** | **Search Strategy** | **Results** |
| PubMed | (("magnetic resonance imaging"[Title/Abstract] OR "functional mri"[Title/Abstract] OR "fmri"[Title/Abstract] OR "magnetic resonance spectroscopy"[Title/Abstract] OR "diffusion tensor imaging"[Title/Abstract] OR "white matter"[Title/Abstract] OR "voxel-based morphometry"[Title/Abstract] OR "gray matter"[Title/Abstract] OR "connectivity"[Title/Abstract] OR "rest"[Title/Abstract] OR "resting state"[Title/Abstract] OR "x-ray"[Title/Abstract] OR "computed tomography"[Title/Abstract] OR "positron-emission tomography"[Title/Abstract] OR "positron-emission tomography"[Title/Abstract] OR "spectroscopy"[Title/Abstract] OR "near infrared spectrometry"[Title/Abstract] OR "near-infrared"[Title/Abstract]) AND ("pregnant"[Title/Abstract] OR "gravidity"[Title/Abstract] OR "pregnancy"[Title/Abstract] OR "natal"[Title/Abstract] OR "peri-natal"[Title/Abstract] OR "perinatal"[Title/Abstract] OR "peripartum"[Title/Abstract] OR "peripartum period"[Title/Abstract] OR "postpartum"[Title/Abstract] OR "postpartum period"[Title/Abstract] OR "antenatal"[Title/Abstract] OR "maternity"[Title/Abstract] OR "gestation"[Title/Abstract] OR "pregnancy"[Title/Abstract] OR "motherhood"[Title/Abstract] OR "mother"[Title/Abstract] OR "mothers"[Title/Abstract] OR "prepartum"[Title/Abstract] OR "prenatal"[Title/Abstract] OR "birth"[Title/Abstract] OR "parturition"[Title/Abstract] OR "delivery"[Title/Abstract] OR "obsteric"[Title/Abstract] OR "paternal"[Title/Abstract] OR "father"[Title/Abstract] OR "fathers"[Title/Abstract] OR "fatherhood"[Title/Abstract]) AND ("depression"[Title/Abstract] OR "depressive disorder"[Title/Abstract])) AND ((booksdocs[Filter] OR clinicaltrial[Filter] OR randomizedcontrolledtrial[Filter]) AND (humans[Filter])) | 41 |
| PsycINFO (through Ovid) | 1     (("magnetic resonance imaging" or "functional mri" or "fmri" or "magnetic resonance spectroscopy" or "diffusion tensor imaging" or "white matter" or "voxel-based morphometry" or "gray matter" or "connectivity" or "rest" or "resting state" or "x-ray" or "computed tomography" or "positron-emission tomography" or "positron emission tomography" or "spectroscopy" or "near infrared spectrometry" or "near-infrared") and ("pregnant" or "gravidity" or "pregnancy" or "natal" or "peri-natal" or "perinatal" or "peripartum" or "peripartum period" or "postpartum" or "postpartum period" or "antenatal" or "maternity" or "gestation" or "pregnancy" or "motherhood" or "mother" or "mothers" or "prepartum" or "prenatal" or "birth" or "parturition" or "delivery" or "obsteric" or "paternal" or "father" or "fathers" or "fatherhood") and ("depression" or "depressive disorder")).ab. or (("magnetic resonance imaging" or "functional mri" or "fmri" or "magnetic resonance spectroscopy" or "diffusion tensor imaging" or "white matter" or "voxel-based morphometry" or "gray matter" or "connectivity" or "rest" or "resting state" or "x-ray" or "computed tomography" or "positron-emission tomography" or "positron emission tomography" or "spectroscopy" or "near infrared spectrometry" or "near-infrared") and ("pregnant" or "gravidity" or "pregnancy" or "natal" or "peri-natal" or "perinatal" or "peripartum" or "peripartum period" or "postpartum" or "postpartum period" or "antenatal" or "maternity" or "gestation" or "pregnancy" or "motherhood" or "mother" or "mothers" or "prepartum" or "prenatal" or "birth" or "parturition" or "delivery" or "obsteric" or "paternal" or "father" or "fathers" or "fatherhood") and ("depression" or "depressive disorder")).ti. (263)  2     limit 1 to human (238)  3     2 and "Peer Reviewed Journal".sa_pubt. (213)  4     3 and "Journal".sa_pubt. (213) | 213 |
| Embase | ('magnetic resonance imaging':ti,ab OR 'functional mri':ti,ab OR fmri:ti,ab OR 'magnetic resonance spectroscopy':ti,ab OR 'diffusion tensor imaging':ti,ab OR 'white matter':ti,ab OR 'voxel-based morphometry':ti,ab OR 'gray matter':ti,ab OR connectivity:ti,ab OR rest:ti,ab OR 'resting state':ti,ab OR 'x ray':ti,ab OR 'computed tomography':ti,ab OR 'positron-emission tomography':ti,ab OR 'positron emission tomography':ti,ab OR spectroscopy:ti,ab OR 'near infrared spectrometry':ti,ab OR 'near infrared':ti,ab) AND (pregnant:ti,ab OR gravidity:ti,ab OR natal:ti,ab OR 'peri natal':ti,ab OR perinatal:ti,ab OR peripartum:ti,ab OR 'peripartum period':ti,ab OR postpartum:ti,ab OR 'postpartum period':ti,ab OR antenatal:ti,ab OR maternity:ti,ab OR gestation:ti,ab OR pregnancy:ti,ab OR motherhood:ti,ab OR mother:ti,ab OR mothers:ti,ab OR prepartum:ti,ab OR prenatal:ti,ab OR birth:ti,ab OR parturition:ti,ab OR delivery:ti,ab OR obsteric:ti,ab OR paternal:ti,ab OR father:ti,ab OR fathers:ti,ab OR fatherhood:ti,ab) AND (depression:ti,ab OR 'depressive disorder':ti,ab) AND ([article]/lim OR [article in press]/lim OR [conference paper]/lim OR [data papers]/lim OR [editorial]/lim OR [letter]/lim OR [note]/lim OR [short survey]/lim) AND [humans]/lim | 436 |
| **Search 2** | **MDD** | |
| PubMed | (("magnetic resonance imaging"[Title/Abstract] OR "functional mri"[Title/Abstract] OR "fmri"[Title/Abstract] OR "magnetic resonance spectroscopy"[Title/Abstract] OR "diffusion tensor imaging"[Title/Abstract] OR "white matter"[Title/Abstract] OR "voxel-based morphometry"[Title/Abstract] OR "gray matter"[Title/Abstract] OR "connectivity"[Title/Abstract] OR "rest"[Title/Abstract] OR "resting state"[Title/Abstract] OR "x-ray"[Title/Abstract] OR "computed tomography"[Title/Abstract] OR "positron-emission tomography"[Title/Abstract] OR "positron-emission tomography"[Title/Abstract] OR "spectroscopy"[Title/Abstract] OR "near infrared spectrometry"[Title/Abstract] OR "near-infrared"[Title/Abstract]) AND ("depression"[Title/Abstract] OR "depressive disorder"[Title/Abstract])) AND ((booksdocs[Filter] OR clinicaltrial[Filter] OR randomizedcontrolledtrial[Filter]) AND (humans[Filter])) | 3505 |
| PsycINFO (through Ovid) | 1 (("magnetic resonance imaging" or "functional mri" or "fmri" or "magnetic resonance spectroscopy" or "diffusion tensor imaging" or "white matter" or "voxel-based morphometry" or "gray matter" or "connectivity" or "rest" or "resting state" or "x-ray" or "computed tomography" or "positron-emission tomography" or "positron emission tomography" or "spectroscopy" or "near infrared spectrometry" or "near-infrared") and ("depression" or "depressive disorder")).ab. or (("magnetic resonance imaging" or "functional mri" or "fmri" or "magnetic resonance spectroscopy" or "diffusion tensor imaging" or "white matter" or "voxel-based morphometry" or "gray matter" or "connectivity" or "rest" or "resting state" or "x-ray" or "computed tomography" or "positron-emission tomography" or "positron emission tomography" or "spectroscopy" or "near infrared spectrometry" or "near-infrared") and ("depression" or "depressive disorder")).ti. (8540)  2 limit 1 to human (7785)  3 2 and "Journal".sa_pubt. (7241)  4 3 and "Peer Reviewed Journal".sa_pubt. (7222)  5 4 and "Major Depression".sa_suba. (4450) | 4450 |
| Embase | ('magnetic resonance imaging':ti,ab OR 'functional mri':ti,ab OR fmri:ti,ab OR 'magnetic resonance spectroscopy':ti,ab OR 'diffusion tensor imaging':ti,ab OR 'white matter':ti,ab OR 'voxel-based morphometry':ti,ab OR 'gray matter':ti,ab OR connectivity:ti,ab OR rest:ti,ab OR 'resting state':ti,ab OR 'x ray':ti,ab OR 'computed tomography':ti,ab OR 'positron-emission tomography':ti,ab OR 'positron emission tomography':ti,ab OR spectroscopy:ti,ab OR 'near infrared spectrometry':ti,ab OR 'near infrared':ti,ab) AND (depression:ti,ab OR 'depressive disorder':ti,ab) AND ([article]/lim OR [article in press]/lim OR [conference paper]/lim OR [data papers]/lim OR [editorial]/lim OR [letter]/lim OR [note]/lim OR [short survey]/lim) AND [humans]/lim AND 'major depression'/dm | 1426 |
| **Updated Search 1 - PPD**  **27/02/2023** | | |
| PubMed | (("magnetic resonance imaging"[Title/Abstract] OR "functional mri"[Title/Abstract] OR "fmri"[Title/Abstract] OR "magnetic resonance spectroscopy"[Title/Abstract] OR "diffusion tensor imaging"[Title/Abstract] OR "white matter"[Title/Abstract] OR "voxel-based morphometry"[Title/Abstract] OR "gray matter"[Title/Abstract] OR "connectivity"[Title/Abstract] OR "rest"[Title/Abstract] OR "resting state"[Title/Abstract] OR "x-ray"[Title/Abstract] OR "computed tomography"[Title/Abstract] OR "positron-emission tomography"[Title/Abstract] OR "spectroscopy"[Title/Abstract] OR "near infrared spectrometry"[Title/Abstract] OR "near-infrared"[Title/Abstract]) AND ("pregnant"[Title/Abstract] OR "gravidity"[Title/Abstract] OR "pregnancy"[Title/Abstract] OR "natal"[Title/Abstract] OR "peri-natal"[Title/Abstract] OR "perinatal"[Title/Abstract] OR "peripartum"[Title/Abstract] OR "peripartum period"[Title/Abstract] OR "postpartum"[Title/Abstract] OR "postpartum period"[Title/Abstract] OR "antenatal"[Title/Abstract] OR "maternity"[Title/Abstract] OR "gestation"[Title/Abstract] OR "motherhood"[Title/Abstract] OR "mother"[Title/Abstract] OR "mothers"[Title/Abstract] OR "prepartum"[Title/Abstract] OR "prenatal"[Title/Abstract] OR "birth"[Title/Abstract] OR "parturition"[Title/Abstract] OR "delivery"[Title/Abstract] OR "obstetric"[Title/Abstract] OR "paternal"[Title/Abstract] OR "father"[Title/Abstract] OR "fathers"[Title/Abstract] OR "fatherhood"[Title/Abstract]) AND ("depression"[Title/Abstract] OR "depressive disorder"[Title/Abstract])) AND ((booksdocs[Filter] OR clinicaltrial[Filter] OR randomizedcontrolledtrial[Filter]) AND (humans[Filter]) AND (2021/9/24:2023/2/24[pdat])) | 3 |
| PsycINFO (through Ovid) | 1 (("magnetic resonance imaging" or "functional mri" or "fmri" or "magnetic resonance spectroscopy" or "diffusion tensor imaging" or "white matter" or "voxel-based morphometry" or "gray matter" or "connectivity" or "rest" or "resting state" or "x-ray" or "computed tomography" or "positron-emission tomography" or "positron emission tomography" or "spectroscopy" or "near infrared spectrometry" or "near-infrared") and ("pregnant" or "gravidity" or "pregnancy" or "natal" or "peri-natal" or "perinatal" or "peripartum" or "peripartum period" or "postpartum" or "postpartum period" or "antenatal" or "maternity" or "gestation" or "pregnancy" or "motherhood" or "mother" or "mothers" or "prepartum" or "prenatal" or "birth" or "parturition" or "delivery" or "obsteric" or "paternal" or "father" or "fathers" or "fatherhood") and ("depression" or "depressive disorder")).ab. or (("magnetic resonance imaging" or "functional mri" or "fmri" or "magnetic resonance spectroscopy" or "diffusion tensor imaging" or "white matter" or "voxel-based morphometry" or "gray matter" or "connectivity" or "rest" or "resting state" or "x-ray" or "computed tomography" or "positron-emission tomography" or "positron emission tomography" or "spectroscopy" or "near infrared spectrometry" or "near-infrared") and ("pregnant" or "gravidity" or "pregnancy" or "natal" or "peri-natal" or "perinatal" or "peripartum" or "peripartum period" or "postpartum" or "postpartum period" or "antenatal" or "maternity" or "gestation" or "pregnancy" or "motherhood" or "mother" or "mothers" or "prepartum" or "prenatal" or "birth" or "parturition" or "delivery" or "obsteric" or "paternal" or "father" or "fathers" or "fatherhood") and ("depression" or "depressive disorder")).ti. 321  2 limit 1 to human 286  3 limit 2 to peer reviewed journal 257  4 limit 3 to yr="2021 -Current" 37  5 limit 4 to "Journal".sa_pubt. 37 | 37 |
| Embase | ('magnetic resonance imaging':ti,ab OR 'functional mri':ti,ab OR fmri:ti,ab OR 'magnetic resonance spectroscopy':ti,ab OR 'diffusion tensor imaging':ti,ab OR 'white matter':ti,ab OR 'voxel-based morphometry':ti,ab OR 'gray matter':ti,ab OR connectivity:ti,ab OR rest:ti,ab OR 'resting state':ti,ab OR 'x ray':ti,ab OR 'computed tomography':ti,ab OR 'positron-emission tomography':ti,ab OR 'positron emission tomography':ti,ab OR spectroscopy:ti,ab OR 'near infrared spectrometry':ti,ab OR 'near infrared':ti,ab) AND (pregnant:ti,ab OR gravidity:ti,ab OR natal:ti,ab OR 'peri natal':ti,ab OR perinatal:ti,ab OR peripartum:ti,ab OR 'peripartum period':ti,ab OR postpartum:ti,ab OR 'postpartum period':ti,ab OR antenatal:ti,ab OR maternity:ti,ab OR gestation:ti,ab OR pregnancy:ti,ab OR motherhood:ti,ab OR mother:ti,ab OR mothers:ti,ab OR prepartum:ti,ab OR prenatal:ti,ab OR birth:ti,ab OR parturition:ti,ab OR delivery:ti,ab OR obsteric:ti,ab OR paternal:ti,ab OR father:ti,ab OR fathers:ti,ab OR fatherhood:ti,ab) AND (depression:ti,ab OR 'depressive disorder':ti,ab) AND ([article]/lim OR [article in press]/lim OR [conference paper]/lim OR [data papers]/lim OR [editorial]/lim OR [letter]/lim OR [note]/lim OR [short survey]/lim) AND [humans]/lim | 107 |
| **Final Updated Search 1 - PPD**  **25/07/2024** | | |
| PubMed | (("magnetic resonance imaging"[Title/Abstract] OR "functional mri"[Title/Abstract] OR "fmri"[Title/Abstract] OR "magnetic resonance spectroscopy"[Title/Abstract] OR "diffusion tensor imaging"[Title/Abstract] OR "white matter"[Title/Abstract] OR "voxel-based morphometry"[Title/Abstract] OR "gray matter"[Title/Abstract] OR "connectivity"[Title/Abstract] OR "rest"[Title/Abstract] OR "resting state"[Title/Abstract] OR "x-ray"[Title/Abstract] OR "computed tomography"[Title/Abstract] OR "positron-emission tomography"[Title/Abstract] OR "spectroscopy"[Title/Abstract] OR "near infrared spectrometry"[Title/Abstract] OR "near-infrared"[Title/Abstract]) AND ("pregnant"[Title/Abstract] OR "gravidity"[Title/Abstract] OR "pregnancy"[Title/Abstract] OR "natal"[Title/Abstract] OR "peri-natal"[Title/Abstract] OR "perinatal"[Title/Abstract] OR "peripartum"[Title/Abstract] OR "peripartum period"[Title/Abstract] OR "postpartum"[Title/Abstract] OR "postpartum period"[Title/Abstract] OR "antenatal"[Title/Abstract] OR "maternity"[Title/Abstract] OR "gestation"[Title/Abstract] OR "motherhood"[Title/Abstract] OR "mother"[Title/Abstract] OR "mothers"[Title/Abstract] OR "prepartum"[Title/Abstract] OR "prenatal"[Title/Abstract] OR "birth"[Title/Abstract] OR "parturition"[Title/Abstract] OR "delivery"[Title/Abstract] OR "obstetric"[Title/Abstract] OR "paternal"[Title/Abstract] OR "father"[Title/Abstract] OR "fathers"[Title/Abstract] OR "fatherhood"[Title/Abstract]) AND ("depression"[Title/Abstract] OR "depressive disorder"[Title/Abstract]) AND "humans"[MeSH Terms]) AND ((humans[Filter]) AND (2023:2024[pdat])) | 80 |
| PsycINFO (through ProQuest) | abstract((("magnetic resonance imaging" OR "functional mri" OR "fmri" OR "magnetic resonance spectroscopy" OR "diffusion tensor imaging" OR "white matter" OR "voxel-based morphometry" OR "gray matter" OR "connectivity" OR "rest" OR "resting state" OR "x-ray" OR "computed tomography" OR "positron-emission tomography" OR "positron emission tomography" OR "spectroscopy" OR "near infrared spectrometry" OR "near-infrared") AND ("pregnant" OR "gravidity" OR "pregnancy" OR "natal" OR "peri-natal" OR "perinatal" OR "peripartum" OR "peripartum period" OR "postpartum" OR "postpartum period" OR "antenatal" OR "maternity" OR "gestation" OR "pregnancy" OR "motherhood" OR "mother" OR "mothers" OR "prepartum" OR "prenatal" OR "birth" OR "parturition" OR "delivery" OR "obsteric" OR "paternal" OR "father" OR "fathers" OR "fatherhood") AND ("depression" OR "depressive disorder")) .ab. OR (("magnetic resonance imaging" OR "functional mri" OR "fmri" OR "magnetic resonance spectroscopy" OR "diffusion tensor imaging" OR "white matter" OR "voxel-based morphometry" OR "gray matter" OR "connectivity" OR "rest" OR "resting state" OR "x-ray" OR "computed tomography" OR "positron-emission tomography" OR "positron emission tomography" OR "spectroscopy" OR "near infrared spectrometry" OR "near-infrared") AND ("pregnant" OR "gravidity" OR "pregnancy" OR "natal" OR "peri-natal" OR "perinatal" OR "peripartum" OR "peripartum period" OR "postpartum" OR "postpartum period" OR "antenatal" OR "maternity" OR "gestation" OR "pregnancy" OR "motherhood" OR "mother" OR "mothers" OR "prepartum" OR "prenatal" OR "birth" OR "parturition" OR "delivery" OR "obsteric" OR "paternal" OR "father" OR "fathers" OR "fatherhood") AND ("depression" OR "depressive disorder")))  Additional limits - Date: From 2023 to 2024 | 40 |
| Embase | ('magnetic resonance imaging':ti,ab OR 'functional mri':ti,ab OR fmri:ti,ab OR 'magnetic resonance spectroscopy':ti,ab OR 'diffusion tensor imaging':ti,ab OR 'white matter':ti,ab OR 'voxel-based morphometry':ti,ab OR 'gray matter':ti,ab OR connectivity:ti,ab OR rest:ti,ab OR 'resting state':ti,ab OR 'x ray':ti,ab OR 'computed tomography':ti,ab OR 'positron-emission tomography':ti,ab OR 'positron emission tomography':ti,ab OR spectroscopy:ti,ab OR 'near infrared spectrometry':ti,ab OR 'near infrared':ti,ab) AND (pregnant:ti,ab OR gravidity:ti,ab OR natal:ti,ab OR 'peri natal':ti,ab OR perinatal:ti,ab OR peripartum:ti,ab OR 'peripartum period':ti,ab OR postpartum:ti,ab OR 'postpartum period':ti,ab OR antenatal:ti,ab OR maternity:ti,ab OR gestation:ti,ab OR pregnancy:ti,ab OR motherhood:ti,ab OR mother:ti,ab OR mothers:ti,ab OR prepartum:ti,ab OR prenatal:ti,ab OR birth:ti,ab OR parturition:ti,ab OR delivery:ti,ab OR obsteric:ti,ab OR paternal:ti,ab OR father:ti,ab OR fathers:ti,ab OR fatherhood:ti,ab) AND (depression:ti,ab OR 'depressive disorder':ti,ab) AND ([article]/lim OR [article in press]/lim OR [conference paper]/lim OR [data papers]/lim OR [editorial]/lim OR [letter]/lim OR [note]/lim OR [short survey]/lim) AND [humans]/lim AND [2023-2024]/py | 91 |
| **Final Updated Search 2 - MDD**  **25/07/2024** | | |
| PubMed | a | 1297 |
| PsycINFO (through ProQuest) | abstract((("magnetic resonance imaging" OR "functional mri" OR "fmri" OR "magnetic resonance spectroscopy" OR "diffusion tensor imaging" OR "white matter" OR "voxel-based morphometry" OR "gray matter" OR "connectivity" OR "rest" OR "resting state" OR "x-ray" OR "computed tomography" OR "positron-emission tomography" OR "positron emission tomography" OR "spectroscopy" OR "near infrared spectrometry" OR "near-infrared") AND ("depression" OR "depressive disorder")) .ab. OR (("magnetic resonance imaging" OR "functional mri" OR "fmri" OR "magnetic resonance spectroscopy" OR "diffusion tensor imaging" OR "white matter" OR "voxel-based morphometry" OR "gray matter" OR "connectivity" OR "rest" OR "resting state" OR "x-ray" OR "computed tomography" OR "positron-emission tomography" OR "positron emission tomography" OR "spectroscopy" OR "near infrared spectrometry" OR "near-infrared") AND ("depression" OR "depressive disorder")))  Additional limits - Date: From 2021 to 2024; Population: Female, Human | 1293 |
| Embase | ('magnetic resonance imaging':ti,ab OR 'functional mri':ti,ab OR fmri:ti,ab OR 'magnetic resonance spectroscopy':ti,ab OR 'diffusion tensor imaging':ti,ab OR 'white matter':ti,ab OR 'voxel-based morphometry':ti,ab OR 'gray matter':ti,ab OR connectivity:ti,ab OR rest:ti,ab OR 'resting state':ti,ab OR 'x ray':ti,ab OR 'computed tomography':ti,ab OR 'positron-emission tomography':ti,ab OR 'positron emission tomography':ti,ab OR spectroscopy:ti,ab OR 'near infrared spectrometry':ti,ab OR 'near infrared':ti,ab) AND (depression:ti,ab OR 'depressive disorder':ti,ab) AND ([article]/lim OR [article in press]/lim OR [conference paper]/lim OR [data papers]/lim OR [editorial]/lim OR [letter]/lim OR [note]/lim OR [short survey]/lim) AND [humans]/lim AND 'major depression'/dm AND [2021-2024]/py AND [female]/lim | 1367 |

**Figure S1. Flow diagram of selection process.**


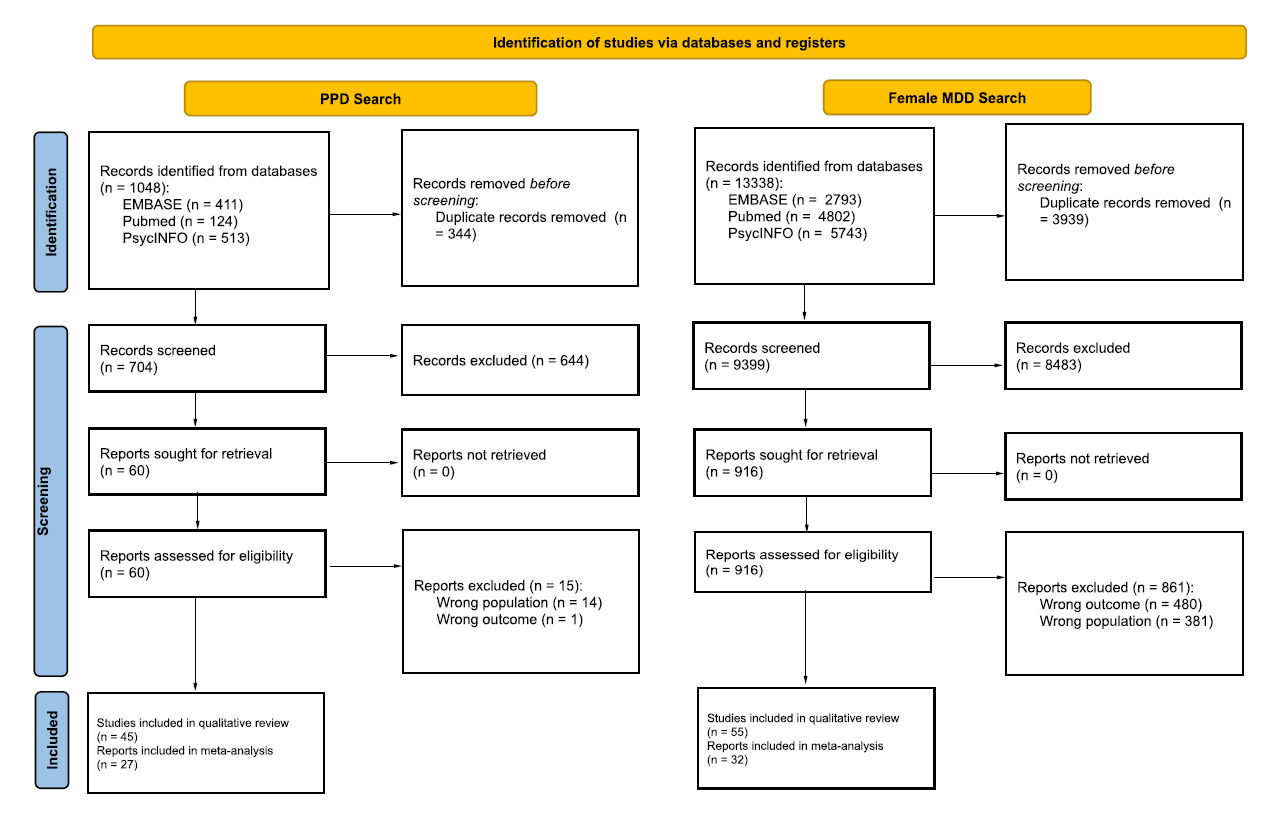


(n = 25)

**Table S3. Comprehensive MDD meta-analysis**

The pooled meta-analysis (367 experiments, 11378 participants, 2850 foci) of individuals diagnosed with MDD, compared with HC (or depression severity correlation), demonstrated neural changes in clusters located in several brain regions related to emotional and cognitive processing, specifically the right ventromedial PFC (VMPFC), the bilateral amygdala, the left putamen, and the right insula.

| **Cluster** | **Volume (mm3)** | **Hem** | **Region** | **ALE** | **P** | **Z** | **MNI Coordinates** | | |
| --- | --- | --- | --- | --- | --- | --- | --- | --- | --- |
|  |  |  |  |  |  |  | **x** | **y** | **z** |
| 1 | 3808 | L | amygdala | 0.0757 | <0.001 | 6.82 | -22 | -4 | -16 |
| 2 | 3072 | R | amygdala | 0.0881 | <0.001 | 7.72 | 22 | -4 | -16 |
| 3 | 2744 | R | VMPFC | 0.047 | <0.001 | 4.47 | 4 | 40 | -10 |
| 4 | 2120 | L | putamen | 0.053 | <0.001 | 4.99 | -24 | 4 | 4 |
| 5 | 1896 | R | insula | 0.0489 | <0.001 | 4.64 | 40 | 10 | -2 |

For each cluster volume, hemisphere, region, ALE value, peak p, z and MNI coordinates are provided.

**Figure S2. Results of ALE meta-analysis showing clusters with significant ALE maxima in MDD patients, superimposed on an MNI-normalized template.**


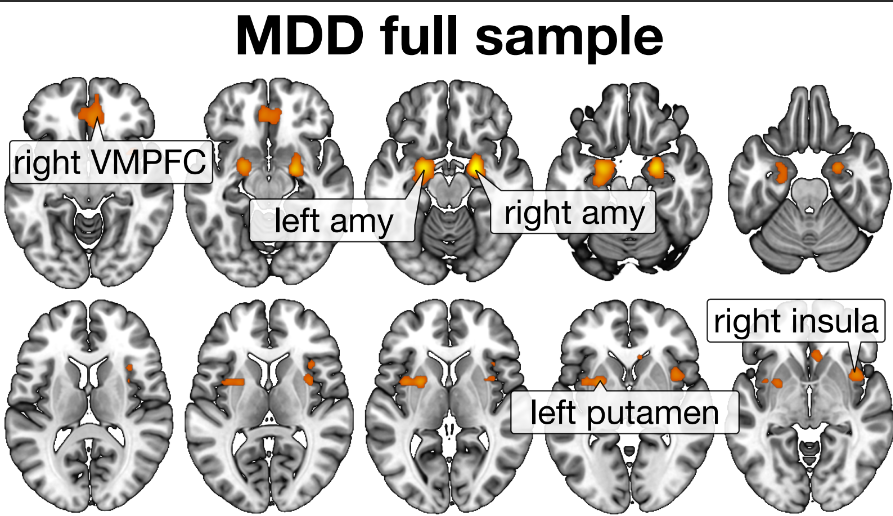


**Table S4. Comparison of PPD and fMDD studies**

|  | **PPD** | **fMDD** |
| --- | --- | --- |
| *Diagnosis according to standard criteria (e.g., DSM)* | 91% | 98% |
| *Peripartum timepoint* | 96% postpartum | - |
| *Previous or current pregnancy* | - | 43.6% excluded current pregnancy or breastfeeding  32.7% with participants in reproductive stage |
| *Clinical comorbidity and characteristics* | 26.7% anxiety disorders/symptoms | 7.3% with anxiety disorders/symptoms |
| *Treatment status* | 91% treatment-free or naive | 52.7% treatment-free or naive |

**Figure S3. Imaging modalities in PPD and fMDD**

**
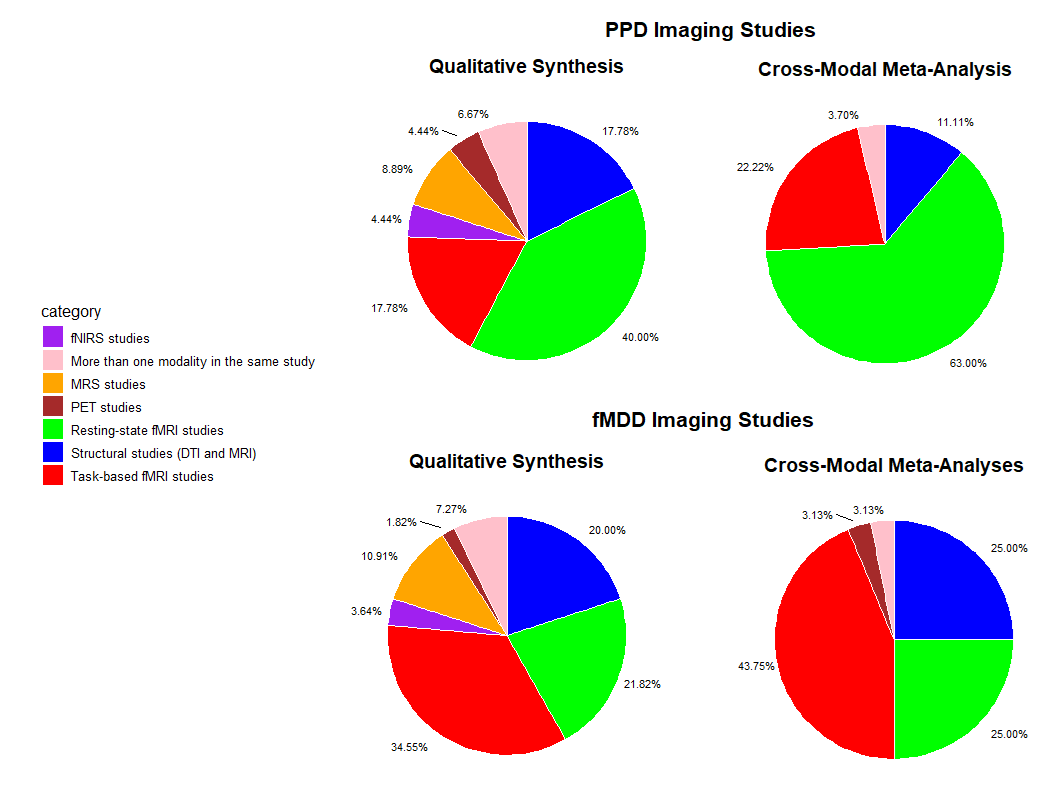
**

**Figure S4. Sociodemographic and clinical characteristics in PPD studies**

**
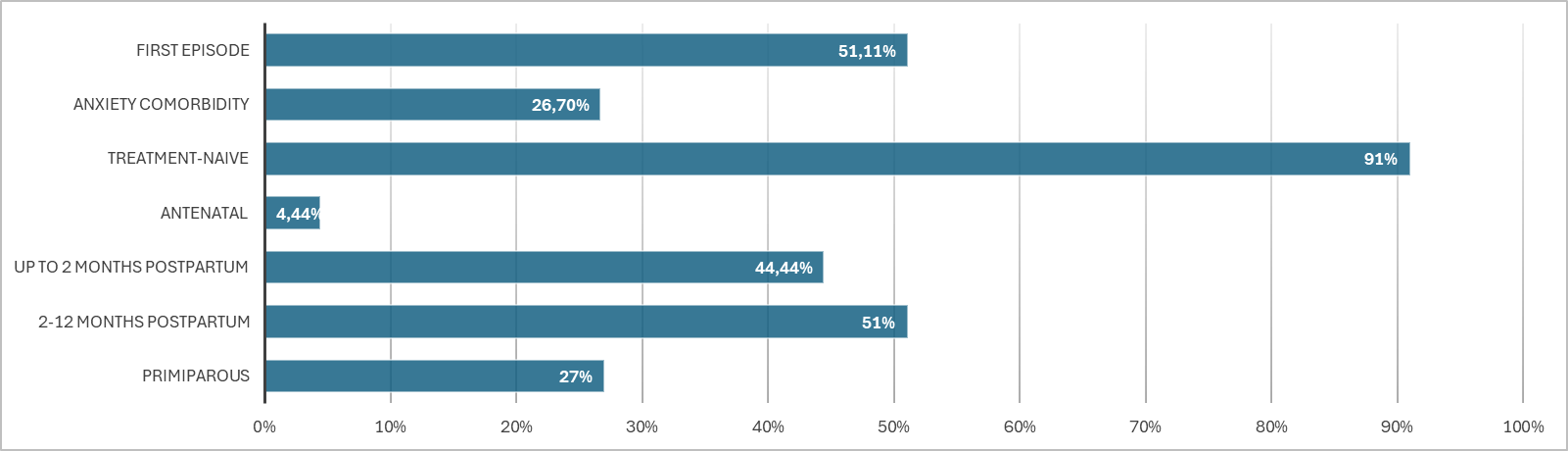
**

**Figure S5. Sociodemographic and clinical characteristics in fMDD studies**

**
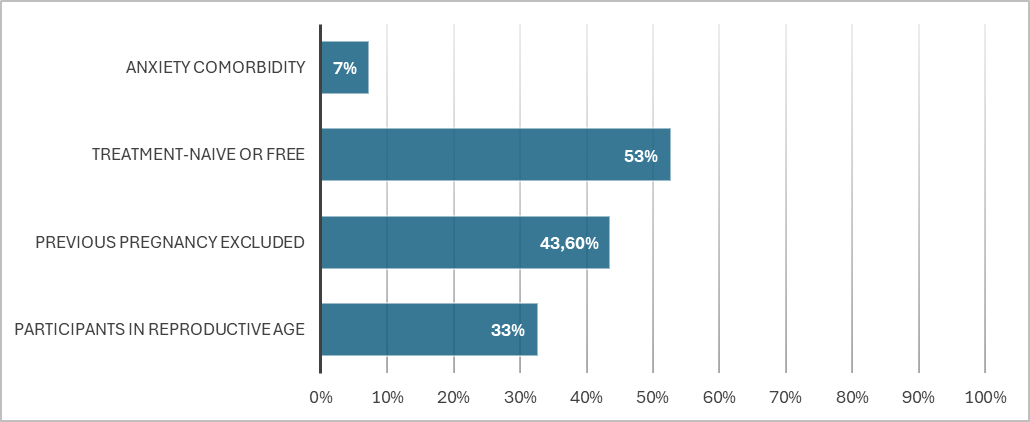
**

**Table S5. Summary of risk of bias assessment for cross-sectional PPD studies.**

| Reference | Item1 | Item2 | Item3 | Item4 | Item5 | Item6 | Item7 | Item8 | Overall Appraisal |
| --- | --- | --- | --- | --- | --- | --- | --- | --- | --- |
| [35] Cheng et al. (2021) | Y | Y | Y | Y | Y | Y | Y | Y | Include (high quality) |
| [36] Cheng et al. (2022) | Y | Y | Y | Y | Y | Y | Y | Y | Include (high quality) |
| [53] Cheng et al. (2021) | Y | Y | Y | Y | Y | Y | Y | Y | Include (high quality) |
| [74] Song et al. (2023) | N | N | Y | Y | N | N | Y | Y | Seek further info (moderate quality) |
| [60] Mao et al. (2020) | Y | N | Y | Y | Y | Y | Y | Y | Include (high quality) |
| [52] Cheng et al. (2022) | Y | Y | Y | Y | N | N | Y | Y | Include (high quality) |
| [66] Dudin et al. (2019) | Y | Y | Y | Y | Y | Y | Y | Y | Include (high quality) |
| [56] Dong et al. (2022) | Y | N | Y | Y | Y | Y | Y | Y | Include (high quality) |
| [75] De Rezende et al. (2018) | Y | Y | Y | Y | N | N | Y | Y | Include (high quality) |
| [78] Rosa et al. (2017) | Y | Y | Y | Y | Y | Y | Y | Y | Include (high quality) |
| [63] Zhang et al. (2022) | Y | Y | Y | Y | Y | Y | Y | Y | Include (high quality) |
| [64] Zhang et al. (2022) | Y | Y | Y | Y | Y | Y | Y | Y | Include (high quality) |
| [44] Cheng et al. (2022) | Y | Y | Y | Y | Y | Y | Y | Y | Include (high quality) |
| [54] Chase et al. (2014) | Y | Y | Y | Y | Y | Y | Y | Y | Include (high quality) |
| [79] Sacher et al. (2015) | Y | N | Y | Y | N | N | Y | Y | Include (high quality) |
| [77] McEwen et al. (2012) | Y | N | Y | Y | Y | Y | Y | Y | Include (high quality) |
| [80] Moses-Kolko et al. (2012) | Y | Y | Y | Y | Y | Y | Y | Y | Include (high quality) |
| [49] Yang et al. (2023) | Y | Y | Y | Y | Y | Y | Y | Y | Include (high quality) |
| [69] Moses-Kolko et al. (2011) | Y | Y | Y | Y | Y | Y | Y | Y | Include (high quality) |
| [71] Silverman et al. (2011) | Y | N | Y | Y | N | N | Y | Y | Include (high quality) |
| [47] Li et al. (2021) | Y | N | Y | Y | Y | Y | Y | Y | Include (high quality) |
| [21] Silverman et al. (2007) | Y | Y | Y | Y | N | N | Y | Y | Include (high quality) |
| [76] Epperson et al. (2006) | Y | N | Y | Y | N | N | Y | Y | Include (high quality) |
| [48] Li et al. (2021) | Y | N | Y | Y | N | N | Y | Y | Include (high quality) |
| [51] Che et al. (2020) | Y | N | Y | Y | Y | Y | Y | Y | Include (high quality) |
| [65] Zhang et al. (2020) | Y | Y | Y | Y | Y | Y | Y | Y | Include (high quality) |
| [54] Cheng et al. (2020) | Y | Y | Y | Y | Y | Y | Y | Y | Include (high quality) |
| [73] Morgan et al. (2021) | Y | Y | Y | Y | Y | Y | Y | Y | Include (high quality) |
| [62] Xu et al. (2023) | Y | Y | Y | Y | Y | Y | Y | Y | Include (high quality) |
| [68] Lenzi et al. (2016) | Y | N | Y | N | N | N | Y | Y | Seek further info (moderate quality) |
| [72] Wonch et al. (2016) | Y | Y | Y | Y | Y | Y | Y | Y | Include (high quality) |
| [40] Long et al. (2023) | Y | Y | Y | Y | N | N | Y | Y | Include (high quality) |
| [59] Li et al. (2023) | Y | Y | Y | Y | Y | Y | Y | Y | Include (high quality) |
| [55] Cheng et al. (2022) | Y | N | Y | Y | Y | Y | Y | Y | Include (high quality) |
| [61] Xiao-juan et al. (2011) | N | N | Y | Y | N | N | Y | Y | Seek further info (moderate quality) |
| [70] Moses-Kolko et al. (2010) | Y | Y | Y | Y | Y | Y | Y | Y | Include (high quality) |
| [43] Chen et al. (2023) | Y | Y | Y | Y | Y | Y | Y | Y | Include (high quality) |
| [37] Chen et al. (2024) | Y | Y | Y | Y | Y | Y | Y | Y | Include (high quality) |
| [46] Huang et al. (2023) | Y | N | Y | Y | Y | Y | Y | Y | Include (high quality) |

Item1: Were the criteria for inclusion in the sample clearly defined?; Item2: Were the study subjects and the setting described in detail?; Item3:Was the exposure (neuroimaging) measured in a valid and reliable way?; Item4: Were objective, standard criteria used for measurement of the condition (PPD)?; Item5: Were confounding factors identified?; Item6: Were strategies to deal with confounding factors stated?; Item7: Were the outcomes measured in a valid and reliable way?; Item8: Was appropriate statistical analysis used?

Y, yes; N, no; U, unclear.

**Table S6. Summary of risk of bias assessment for cohort PPD studies.**

| Reference | Item1 | Item2 | Item3 | Item4 | Item5 | Item6 | Item7 | Item8 | Overall Appraisal |
| --- | --- | --- | --- | --- | --- | --- | --- | --- | --- |
| [41] Sasaki et al. (2020) | ★ | ★ | ★ | - | No | ★ | ★ | ★ | High quality |
| [68] Finnegan et al. (2021) | No | ★ | ★ | - | ★ | ★ | ★ | ★ | High quality |
| [42] Silver et al. (2018) | ★ | ★ | ★ | - | ★ | ★ | ★ | No | High quality |
| [57] Deligiannidis et al. (2013) | ★ | ★ | No | - | No | ★ | ★ | ★ | Moderate quality |
| [58] Deligiannidis et al. (2019) | ★ | ★ | ★ | - | ★ | ★ | No | No | Moderate quality |
| [34] Hare et al. (2024) | ★ | ★ | ★ | - | ★ | ★ | ★ | No | High quality |

Item1: Representativeness of the exposed cohort; Item2: Selection of the non-exposed cohort; Item3: Ascertainment of exposure; Item4: Demonstration that outcome of interest was not present at start of study [not applicable]; Item5: Comparability of cohorts on the basis of the design or analysis; Item6: Assessment of outcome; Item7: Was follow-up long enough for outcomes to occur; Item8: Adequacy of follow up of cohorts

**Table S7. Summary of risk of bias assessment for cross-sectional fMDD studies.**

| Reference | Item1 | Item2 | Item3 | Item4 | Item5 | Item6 | Item7 | Item8 | Overall Appraisal |
| --- | --- | --- | --- | --- | --- | --- | --- | --- | --- |
| [134] Nugent et al. (2011) | Y | Y | Y | Y | Y | Y | Y | Y | Include (high quality) |
| [91] Tang et al. (2007) | Y | N | Y | Y | N | N | Y | Y | Include (high quality) |
| [87] Kim et al. (2008) | Y | Y | Y | Y | Y | Y | Y | Y | Include (high quality) |
| [89] Mak et al. (2009) | Y | N | Y | Y | Y | Y | Y | Y | Include (high quality) |
| [84] Depping et al. (2015) | Y | Y | Y | Y | N | N | Y | Y | Include (high quality) |
| [93] Yang et al. (2017) | Y | Y | Y | Y | Y | Y | Y | Y | Include (high quality) |
| [106] Zhang et al. (2016) | N | N | Y | Y | Y | Y | Y | Y | Include (high quality) |
| [103] Tang et al. (2018) | N | N | Y | Y | Y | Y | Y | Y | Include (high quality) |
| [116] Kumari et al. (2003) | N | N | Y | Y | N | N | Y | Y | Seek further info (moderate quality) |
| [119] Mitterschiffthaler et al. (2003) | N | N | Y | Y | N | N | Y | Y | Seek further info (moderate quality) |
| [123] Wagner et al. (2006) | Y | N | Y | Y | Y | Y | Y | Y | Include (high quality) |
| [107] Abler et al. (2007) | Y | Y | Y | Y | N | N | Y | Y | Include (high quality) |
| [110] Bär et al. (2007) | N | Y | Y | Y | Y | Y | Y | Y | Include (high quality) |
| [111] Briceño et al. (2013) | Y | N | Y | Y | Y | Y | Y | Y | Include (high quality) |
| [121] Shao et al. (2015) | Y | Y | Y | Y | N | N | Y | Y | Include (high quality) |
| [125] Yttredahl et al. (2018) | Y | Y | Y | Y | Y | Y | Y | Y | Include (high quality) |
| [117] Malejko et al. (2021) | Y | Y | Y | Y | Y | Y | Y | Y | Include (high quality) |
| [120] Robert et al. (2021) | Y | Y | Y | Y | N | N | Y | Y | Include (high quality) |
| [122] Tak et al. (2021) | Y | Y | Y | Y | N | N | Y | Y | Include (high quality) |
| [104] Teng et al. (2018) | Y | N | Y | Y | Y | Y | Y | Y | Include (high quality) |
| [82] Lyon et al. (2019) | Y | N | Y | Y | Y | Y | Y | Y | Include (high quality) |
| [92] Yang et al. (2017) | Y | Y | Y | Y | Y | Y | Y | Y | Include (high quality) |
| [88] Kong et al. (2013) | Y | Y | Y | Y | N | N | Y | Y | Include (high quality) |
| [86] Hu et al. (2022) | Y | Y | Y | Y | Y | Y | Y | Y | Include (high quality) |
| [83] Carceller-Sindreu et al. (2015) | N | N | Y | Y | Y | Y | Y | Y | Include (high quality) |
| [90] Siragusa et al. (2021) | Y | N | Y | Y | Y | Y | Y | Y | Include (high quality) |
| [81] Domain et al. (2022) | Y | Y | Y | Y | Y | Y | Y | Y | Include (high quality) |
| [85] Hastings et al. (2004) | Y | N | Y | Y | Y | Y | Y | Y | Include (high quality) |
| [132] Zhang et al. (2015) | Y | N | Y | Y | N | N | Y | Y | Include (high quality) |
| [133] Zhong et al. (2024) | Y | Y | Y | Y | Y | Y | Y | Y | Include (high quality) |
| [115] Íronside et al. (2021) | Y | Y | Y | Y | Y | Y | Y | Y | Include (high quality) |
| [128] Kantrowitz et al. (2021) | Y | Y | Y | Y | Y | Y | Y | Y | Include (high quality) |
| [131] Tran et al. (2023) | Y | N | Y | Y | N | N | Y | Y | Include (high quality) |
| [129] Song et al. (2024) | Y | Y | Y | Y | Y | Y | Y | Y | Include (high quality) |
| [130] Tran et al. (2024) | Y | N | Y | Y | N | N | Y | Y | Include (high quality) |
| [126] Lyu et al. (2023) | Y | N | Y | Y | N | N | Y | Y | Include (high quality) |
| [127] Ma et al. (2017) | Y | Y | Y | Y | Y | Y | Y | Y | Include (high quality) |
| [108] Almeida et al. (2011) | Y | N | Y | Y | Y | Y | Y | Y | Include (high quality) |
| [109] Baeken et al. (2010) | Y | Y | Y | Y | Y | Y | Y | Y | Include (high quality) |
| [114] Dong et al. (2022) | Y | Y | Y | Y | Y | Y | Y | Y | Include (high quality) |
| [113] Cane et al. (2023) | Y | N | Y | Y | Y | Y | Y | Y | Include (high quality) |
| [124] Young et al. (2017) | Y | N | Y | Y | N | N | Y | Y | Include (high quality) |
| [99] Mei et al. (2022) | Y | Y | Y | Y | Y | Y | Y | Y | Include (high quality) |
| [98] Li et al. (2022) | Y | Y | Y | Y | Y | Y | Y | Y | Include (high quality) |
| [97] Dong et al. (2024) | Y | Y | Y | Y | Y | Y | Y | Y | Include (high quality) |
| [95] Belleau et al. (2022) | Y | Y | Y | Y | Y | Y | Y | Y | Include (high quality) |
| [94] Amiri et al. (2021) | N | N | Y | Y | N | N | Y | Y | Seek further info (moderate quality) |
| [112] Briceño et al. (2014) | N | N | Y | Y | N | N | Y | Y | Seek further info (moderate quality) |
| [118] Malejko et al. (2021) | Y | N | Y | Y | Y | Y | Y | Y | Include (high quality) |
| [102] Sun et al. (2022) | Y | Y | Y | Y | Y | Y | Y | Y | Include (high quality) |
| [105] Tu et al. (2022) | Y | Y | Y | Y | Y | Y | Y | Y | Include (high quality) |
| [96] Chen et al. (2022) | Y | Y | Y | Y | Y | Y | Y | Y | Include (high quality) |
| [100] Pessin et al. (2022) | Y | Y | Y | Y | Y | Y | Y | Y | Include (high quality) |
| [101] Philippi et al. (2022) | Y | Y | Y | Y | Y | Y | Y | Y | Include (high quality) |

Item1: Were the criteria for inclusion in the sample clearly defined?; Item2: Were the study subjects and the setting described in detail?; Item3: Was the exposure (neuroimaging) measured in a valid and reliable way?; Item4: Were objective, standard criteria used for measurement of the condition (MDD)?; Item5: Were confounding factors identified?; Item6: Were strategies to deal with confounding factors stated?; Item7: Were the outcomes measured in a valid and reliable way?; Item8: Was appropriate statistical analysis used?

Y, yes; N, no; U, unclear.

**Table S8. Results of the ALE meta-analyses on PPD participants, in comparison to HC.**

| **Cluster** | **Volume (mm3)** | **Hem** | **Region** | **ALE** | **P** | **Z** | **MNI Coordinates** | | |
| --- | --- | --- | --- | --- | --- | --- | --- | --- | --- |
|  |  |  |  |  |  |  | **x** | **y** | **z** |
| **Resting-state fMRI** | | | | | | | | | |
| 1 | 1008 | L | middle frontal gyrus | 0.0234 | <0.001 | 5.47 | -26 | 36 | 34 |
| **Multimodal** | | | | | | | | | |
| 1 | 1800 | R | putamen | 0.0197 | <0.001 | 4.75 | 28 | -2 | -12 |
|  |  |  | amygdala | 0.0017 | <0.001 | 4.35 | 24 | -6 | -20 |
|  |  |  | extra-nuclear | 0.0165 | <0.001 | 4.23 | 36 | -2 | -6 |
| 2 | 1064 | L | middle frontal gyrus | 0.0243 | <0.001 | 5.46 | -26 | 36 | 34 |
| **Increased effect** | | | | | | | | | |
| 1 | 10704 | R | amygdala | 0.0122 | <0.001 | 4.12 | 26 | -6 | -22 |
|  |  |  | sub-lobar lateral ventricle | 0.0094 | <0.001 | 3.63 | 32 | -12 | -18 |
|  |  |  | extra-nuclear | 0.0085 | <0.001 | 3.37 | 28 | -20 | -6 |
|  |  |  | extra-nuclear | 0.0082 | <0.001 | 3.29 | 26 | 0 | -14 |
| 2 | 9048 | L | extra-nuclear | 0.0121 | <0.001 | 4.10 | -14 | 16 | -8 |
|  |  |  | frontal lobe sub-gyral | 0.0088 | <0.001 | 3.51 | -24 | 26 | -18 |
|  |  |  | subcallosal gyrus | 0.0077 | <0.001 | 3.12 | -12 | 24 | -20 |
| 3 | 8504 | L | middle frontal gyrus | 0.0122 | <0.001 | 4.11 | -26 | 36 | 34 |
|  |  |  | middle frontal gyrus | 0.0108 | <0.001 | 3.89 | -46 | 24 | 40 |
|  |  |  | middle frontal gyrus | 0.0079 | <0.001 | 3.18 | -28 | 22 | 38 |
| 4 | 6768 | L | precuneus | 0.0126 | <0.001 | 4.18 | -2 | -62 | 42 |
|  |  |  | cingulate gyrus | 0.0106 | <0.001 | 3.86 | -6 | -52 | 30 |
| 5 | 5592 | R | precuneus | 0.0091 | <0.001 | 3.57 | 42 | -74 | 44 |
|  |  |  | angular gyrus | 0.0081 | <0.001 | 3.2 | 36 | -60 | 42 |
|  |  |  | angular gyrus | 0.0064 | <0.05 | 2.72 | 50 | -62 | 40 |
| 6 | 3648 | L | medial frontal gyrus | 0.0081 | <0.001 | 3.26 | 2 | 64 | -14 |
|  |  |  | medial frontal gyrus | 0.0079 | <0.001 | 3.16 | -10 | 64 | -10 |
| 7 | 3616 | R | frontal lobe sub-gyral | 0.0065 | <0.05 | 2.74 | 30 | -20 | 34 |
|  |  |  | frontal lobe sub-gyral | 0.0064 | <0.05 | 2.72 | 30 | -30 | 28 |
| 8 | 3608 | R | cingulate gyrus | 0.0096 | <0.001 | 3.68 | 12 | -16 | 44 |
| 9 | 3552 | L | middle frontal gyrus | 0.0106 | <0.001 | 3.85 | -26 | -8 | 62 |
|  |  |  | frontal lobe sub-gyral | 0.0079 | <0.001 | 3.18 | -18 | -12 | 58 |
| 10 | 3520 | R | superior frontal gyrus | 0.0088 | <0.001 | 3.45 | 12 | 30 | 56 |
|  |  | L | superior frontal gyrus | 0.0081 | <0.001 | 3.2 | -2 | 28 | 52 |
| 11 | 3504 | R | middle frontal gyrus | 0.0088 | <0.001 | 3.42 | 42 | 3 | 54 |
|  |  |  | precentral gyrus | 0.0085 | <0.001 | 3.31 | 51 | 6 | 45 |
| 12 | 2368 | L | lingual gyrus | 0.0085 | <0.001 | 3.36 | -15 | -96 | 0 |
|  |  |  | inferior occipital gyrus | 0.0082 | <0.001 | 3.26 | -15 | -96 | -4 |
| 13 | 2008 | L | middle temporal gyrus | 0.0079 | <0.001 | 3.16 | -48 | -60 | 12 |
| 14 | 2008 | R | anterior cingulate | 0.0091 | <0.001 | 3.55 | 6 | 30 | 18 |
| 15 | 2008 | L | medial frontal gyrus | 0.0087 | <0.001 | 3.4 | 2 | 58 | 32 |
| 16 | 2008 | L | cuneus | 0.0076 | <0.001 | 3.02 | -12 | -82 | 34 |
| 17 | 1912 | R | anterior cingulate | 0.0064 | <0.001 | 2.72 | 24 | 38 | 14 |
| 18 | 1912 | L | anterior cingulate | 0.0082 | <0.001 | 3.23 | -14 | 40 | 18 |
| 19 | 1856 | L | cingulate gyrus | 0.0081 | <0.001 | 3.23 | -2 | 14 | 30 |
| 20 | 1848 | L | insula | 0.008 | <0.001 | 3.18 | -40 | -18 | 20 |
| 21 | 1824 | R | middle temporal gyrus | 0.0077 | <0.001 | 3.12 | 60 | -11 | -24 |
| 22 | 1824 | L | posterior lobe declive | 0.0082 | <0.001 | 3.26 | -45 | -60 | -21 |
| 23 | 1824 | L | inferior occipital gyrus | 0.0085 | <0.001 | 3.31 | -42 | -81 | -3 |
| 24 | 1824 | R | inferior parietal lobule | 0.0079 | <0.001 | 3.16 | 58 | -20 | 28 |
| 25 | 1824 | R | precentral gyrus | 0.0085 | <0.001 | 3.31 | 39 | 21 | 36 |
| 26 | 1824 | L | middle frontal gyrus | 0.0074 | <0.001 | 2.99 | -24 | 15 | 63 |
| 27 | 1816 | L | superior parietal lobule | 0.0079 | <0.001 | 3.18 | -12 | -54 | 66 |
| 28 | 1728 | L | extra-nuclear | 0.0094 | <0.001 | 3.63 | -36 | 6 | -15 |
| 29 | 1688 | R | insula | 0.0103 | <0.001 | 3.78 | 36 | 26 | 8 |
| 30 | 1664 | L | inferior frontal gyrus | 0.0072 | <0.05 | 2.92 | -44 | 32 | -16 |
| 31 | 1664 | L | ventral posterior lateral nucleus (thalamus) | 0.0081 | <0.001 | 3.2 | -15 | -15 | 3 |
| 32 | 1664 | L | frontal lobe sub-gyral | 0.0072 | <0.05 | 2.92 | -32 | 44 | 10 |
| 33 | 1664 | L | temporal lobe sub-gyral | 0.0072 | <0.05 | 2.92 | -32 | -76 | 38 |
| **Decreased effect** | | | | | | | | | |
| 1 | 7696 | R | extra-nuclear | 0.0162 | <0.001 | 4.48 | 36 | -2 | -6 |
|  |  |  | extra-nuclear | 0.0123 | <0.001 | 3.72 | 26 | -2 | -14 |
|  |  |  | insula | 0.0116 | <0.001 | 3.59 | 42 | 8 | 0 |
|  |  |  | extra-nuclear | 0.0089 | <0.001 | 3.02 | 42 | -2 | -14 |
|  |  |  | sub.gyral | 0.0081 | <0.05 | 2.82 | 32 | 4 | -20 |
|  |  |  | insula | 0.0065 | <0.05 | 2.44 | 48 | 6 | 8 |
| 2 | 7416 | L | precentral gyrus | 0.0124 | <0.001 | 3.75 | -22 | -16 | 66 |
|  |  |  | precentral gyrus | 0.0099 | <0.001 | 3.24 | -34 | -24 | 60 |
|  |  |  | precentral gyrus | 0.0079 | <0.05 | 2.76 | -52 | 2 | 44 |
|  |  |  | precentral gyrus | 0.0077 | <0.05 | 2.70 | -42 | -10 | 60 |
|  |  |  | precentral gyrus | 0.0063 | <0.05 | 2.4 | -46 | -6 | 42 |
|  |  |  | precentral gyrus | 0.0063 | <0.05 | 2.39 | -52 | 8 | 44 |
|  |  |  | precentral gyrus | 0.0061 | <0.05 | 2.35 | -48 | 0 | 54 |
|  |  |  | precentral gyrus | 0.0061 | <0.05 | 2.33 | -38 | -20 | 52 |
|  |  |  | precentral gyrus | 0.0057 | <0.05 | 2.25 | -16 | -30 | 64 |
| 3 | 6280 | L | supramarginal gyrus | 0.0013 | <0.001 | 3.92 | -54 | -36 | 38 |
|  |  |  | supramarginal gyrus | 0.0113 | <0.001 | 3.5 | -52 | -48 | 42 |
|  |  |  | inferior parietal lobule | 0.0104 | <0.001 | 3.32 | -42 | -50 | 46 |
|  |  |  | superior temporal gyrus | 0.01 | <0.001 | 3.26 | -62 | -44 | 24 |
|  |  |  | inferior parietal lobule | 0.005 | <0.05 | 2.03 | -62 | -36 | 34 |
| 4 | 6232 | L | superior frontal gyrus | 0.0149 | <0.001 | 4.23 | -4 | 32 | 50 |
|  |  | R | medial frontal gyrus | 0.0128 | <0.001 | 3.83 | 6 | 36 | 34 |
|  |  | L | medial frontal gyrus | 0.0108 | <0.001 | 3.43 | -6 | 42 | 38 |
|  |  | L | superior frontal gyrus | 0.0097 | <0.001 | 3.2 | 2 | 32 | 50 |
|  |  | L | superior frontal gyrus | 0.0091 | <0.001 | 3.07 | -14 | 40 | 44 |
| 5 | 5104 | R | precentral gyrus | 0.0121 | <0.001 | 3.69 | 60 | 0 | 36 |
|  |  |  | precentral gyrus | 0.0079 | <0.05 | 2.76 | 60 | 2 | 18 |
|  |  |  | inferior frontal gyrus | 0.0073 | <0.05 | 2.61 | 50 | 0 | 24 |
|  |  |  | precentral gyrus | 0.0069 | <0.05 | 2.52 | 44 | 2 | 34 |
| 6 | 4392 | R | temporal lobe sub-gyral | 0.0172 | <0.001 | 4.65 | 38 | -54 | -4 |
|  |  |  | posterior lobe declive | 0.0068 | <0.05 | 2.50 | 38 | -62 | -18 |
|  |  |  | fusiform gyrus | 0.0062 | <0.05 | 2.38 | 36 | -72 | -10 |
|  |  |  | posterior lobe declive | 0.0062 | <0.05 | 2.37 | 34 | -62 | -12 |
| 7 | 3832 | L | anterior cingulate | 0.0113 | <0.001 | 3.52 | -10 | 34 | 14 |
|  |  |  | anterior cingulate | 0.0109 | <0.001 | 3.43 | -6 | 52 | 2 |
|  |  |  | medial frontal gyrus | 0.0058 | <0.05 | 2.26 | -10 | 48 | 14 |
| 8 | 3544 | R | middle frontal gyrus | 0.0097 | <0.001 | 3.19 | 30 | 46 | 30 |
|  |  |  | superior frontal gyrus | 0.0083 | <0.05 | 2.85 | 28 | 36 | 50 |
|  |  |  | middle frontal gyrus | 0.0090 | <0.05 | 2.37 | 24 | 42 | 38 |
| 9 | 3248 | L | middle frontal gyrus | 0.0123 | <0.001 | 3.73 | -26 | 36 | 34 |
|  |  |  | frontal lobe sub-gyral | 0.0060 | <0.05 | 2.32 | -30 | 18 | 36 |
|  |  |  | frontal lobe sub-gyral | 0.0058 | <0.05 | 2.28 | -28 | 24 | 34 |
|  |  |  | frontal lobe sub-gyral | 0.0056 | <0.05 | 2.2 | -32 | 22 | 34 |
| 10 | 2880 | R | middle temporal gyrus | 0.0102 | <0.001 | 3.31 | 62 | -42 | 0 |
|  |  |  | middle temporal gyrus | 0.0067 | <0.05 | 2.49 | 60 | -48 | 8 |
|  |  |  | superior temporal gyrus | 0.0062 | <0.05 | 2.37 | 54 | -40 | 6 |
|  |  |  | middle temporal gyrus | 0.0061 | <0.05 | 2.35 | 58 | -36 | -6 |
| 11 | 2504 | L | putamen | 0.0109 | <0.001 | 3.44 | -24 | -2 | 6 |
|  |  |  | caudate head | 0.0062 | <0.05 | 2.38 | -6 | 6 | 0 |
|  |  |  | ventral lateral nucleus | 0.0059 | <0.05 | 2.3 | -16 | -8 | 12 |
| 12 | 2392 | L | cingulate gyrus | 0.0143 | <0.001 | 4.14 | -4 | 10 | 32 |
|  |  |  | cingulate gyrus | 0.0093 | <0.001 | 3.1 | 2 | 10 | 30 |
|  |  |  | cingulate gyrus | 0.006 | <0.05 | 2.32 | -12 | 4 | 28 |
| 13 | 2376 | L | precuneus | 0.0109 | <0.001 | 3.44 | 0 | -52 | 42 |
|  |  |  | precuneus | 0.0063 | <0.05 | 2.40 | -8 | -58 | 34 |
| 14 | 2080 | L | cingulate gyrus | 0.0127 | <0.001 | 3.81 | 0 | -16 | 30 |
|  |  |  | cingulate gyrus | 0.0056 | <0.05 | 2.22 | -8 | -14 | 38 |
| 15 | 1816 | L | inferior semi-lunar lobule | 0.0083 | <0.05 | 2.87 | -20 | -68 | -44 |
|  |  |  | inferior semi-lunar lobule | 0.0082 | <0.05 | 2.82 | -10 | -68 | -44 |
| 16 | 1784 | L | inferior frontal gyrus | 0.0109 | <0.001 | 3.45 | -54 | 12 | 30 |
| 17 | 1760 | R | inferior parietal lobule | 0.0098 | <0.001 | 3.22 | 44 | -63 | 51 |
|  |  |  | angular gyrus | 0.006 | <0.05 | 2.32 | 48 | -58 | 42 |
| 18 | 1752 | L | medial frontal gyrus | 0.0091 | <0.001 | 3.07 | -4 | 30 | -24 |
|  |  |  | medial frontal gyrus | 0.0086 | <0.05 | 2.93 | 2 | 30 | -24 |
|  |  |  | frontal lobe sub-gyral | 0.0083 | <0.05 | 2.86 | -12 | 32 | -22 |
| 19 | 1600 | L | superior temporal gyrus | 0.0107 | <0.001 | 3.4 | -66 | -24 | 6 |
| 20 | 1536 | L | insula | 0.0091 | <0.001 | 3.07 | -42 | 10 | 0 |
|  |  |  | insula | 0.0059 | <0.05 | 2.29 | -42 | 8 | 12 |
|  |  |  | insula | 0.0058 | <0.05 | 2.26 | -42 | 20 | 2 |
| 21 | 1344 | R | anterior cingulate | 0.0148 | <0.001 | 4.22 | 8 | 34 | 14 |
| 22 | 1280 | L | extra-nuclear | 0.0092 | <0.001 | 3.08 | -28 | -4 | -12 |
|  |  |  | amygdala | 0.006 | <0.05 | 2.31 | -30 | -8 | -20 |
| 23 | 1240 | L | fusiform gyrus | 0.0122 | <0.001 | 3.7 | -46 | -60 | -18 |
| 24 | 1208 | L | middle temporal gyrus | 0.0125 | <0.001 | 3.76 | -57 | -66 | 16 |
| 25 | 1160 | L | extra-nuclear | 0.0089 | <0.05 | 3 | -22 | 20 | 4 |
|  |  |  | extra-nuclear | 0.006 | <0.05 | 2.32 | -30 | 18 | 8 |
| 26 | 1000 | L | precentral gyrus | 0.0058 | <0.05 | 2.27 | -52 | -18 | 36 |
|  |  |  | postcentral gyrus | 0.0058 | <0.05 | 2.27 | -56 | -24 | 48 |
|  |  |  | postcentral gyrus | 0.0057 | <0.05 | 2.23 | -52 | -22 | 42 |
| 27 | 992 | R | postcentral gyrus | 0.0081 | <0.05 | 2.81 | 54 | -12 | 52 |
|  |  |  | precentral gyrus | 0.0059 | <0.05 | 2.29 | 52 | 0 | 54 |
| 28 | 960 | R | middle temporal gyrus | 0.0058 | <0.05 | 2.28 | 42 | 2 | -42 |
|  |  |  | middle temporal gyrus | 0.0057 | <0.05 | 2.25 | 48 | -2 | -38 |
|  |  |  | middle temporal gyrus | 0.0055 | <0.05 | 2.18 | 40 | -2 | -44 |
| 29 | 896 | R | putamen | 0.0097 | <0.001 | 3.19 | 24 | -2 | 6 |
| 30 | 808 | R | superior temporal gyrus | 0.0068 | <0.05 | 2.5 | 68 | -14 | 2 |
| 31 | 784 | R | caudate body | 0.0059 | <0.05 | 2.3 | 12 | 14 | 12 |
|  |  |  | caudate body | 0.0059 | <0.05 | 2.3 | 10 | 8 | 8 |
| 32 | 720 | L | inferior frontal gyrus | 0.0058 | <0.05 | 2.28 | -54 | 8 | 12 |
|  |  |  | precentral gyrus | 0.0058 | <0.05 | 2.28 | -54 | 4 | 16 |
| 33 | 688 | L | inferior frontal gyrus | 0.0058 | <0.05 | 2.27 | -42 | 34 | -12 |
|  |  |  | inferior frontal gyrus | 0.0058 | <0.05 | 2.27 | -48 | 30 | -9 |
| 34 | 688 | R | middle frontal gyrus | 0.0058 | <0.05 | 2.28 | 42 | 24 | 40 |
|  |  |  | middle frontal gyrus | 0.0058 | <0.05 | 2.27 | 44 | 30 | 42 |
| 35 | 656 | R | paracentral lobule | 0.0092 | <0.001 | 3.08 | 10 | -28 | 54 |
| 36 | 648 | R | frontal lobe sub-gyral | 0.0092 | <0.001 | 3.09 | 24 | 36 | -12 |
| 37 | 648 | L | frontal lobe sub-gyral | 0.0093 | <0.001 | 3.09 | -24 | 36 | -12 |
| 38 | 648 | R | lingual gyrus | 0.0098 | <0.001 | 3.21 | 18 | -90 | -6 |
| 39 | 640 | R | superior temporal gyrus | 0.0058 | <0.05 | 2.27 | 46 | 12 | -30 |
|  |  |  | superior temporal gyrus | 0.0057 | <0.05 | 2.22 | 38 | 18 | -32 |
| 40 | 640 | R | anterior cingulate | 0.0087 | <0.05 | 2.96 | 12 | 38 | 0 |
| 41 | 640 | L | medial frontal gyrus | 0.0058 | <0.05 | 2.27 | -4 | -6 | 72 |
|  |  |  | medial frontal gyrus | 0.0057 | <0.05 | 2.22 | -4 | -16 | 72 |
| 42 | 632 | R | superior frontal gyrus | 0.0058 | <0.05 | 2.27 | 28 | 54 | 0 |
|  |  |  | medial frontal gyrus | 0.0057 | <0.05 | 2.22 | 20 | 54 | 2 |
| 43 | 616 | R | caudate body | 0.0085 | <0.05 | 2.92 | 20 | 20 | 4 |
| 44 | 584 | R | extra-nuclear | 0.0084 | <0.05 | 2.89 | 16 | 18 | -8 |
| 45 | 584 | L | caudate body | 0.0058 | <0.05 | 2.27 | -10 | 14 | 8 |
|  |  |  | caudate body | 0.0057 | <0.05 | 2.26 | -10 | 18 | 8 |
| 46 | 584 | R | parietal lobe sub-gyral | 0.0058 | <0.05 | 2.27 | 26 | -36 | 60 |
| 47 | 568 | L | middle temporal gyrus | 0.0042 | <0.05 | 1.75 | -53 | 11 | -30 |
| 48 | 560 | L | superior temporal gyrus | 0.0057 | <0.05 | 2.22 | -50 | 4 | -12 |
|  |  |  | superior temporal gyrus | 0.0055 | <0.05 | 2.18 | -50 | -2 | -8 |
| 49 | 512 | R | anterior cingulate | 0.0059 | <0.05 | 2.31 | 2 | 44 | -12 |
| 50 | 512 | R | medial frontal gyrus | 0.0057 | <0.05 | 2.23 | 8 | -6 | 62 |
| 51 | 504 | R | middle temporal gyrus | 0.008 | <0.05 | 2.78 | 44 | -60 | 20 |

For each cluster volume, hemisphere, region, ALE value, peak p, z and MNI coordinates are provided.

**Table S9. Direction of effect analyses in PPD**

Fifteen experiments reported greater activity/connectivity or brain structure in PPD than in HC (384 participants) and 20 experiments reported less (397 participants). For PPD > HC, we observed significant clusters in: bilateral MFG, MTG, precuneus, superior frontal, and cingulate gyri, ACC and insula; right amygdala, sub-lobar lateral ventricle, angular and precentral gyri and inferior parietal lobule; left subcallosal, medial frontal, lingual and inferior occipital gyri, cuneus, posterior lobe declive, superior parietal lobule and ventral posterior lateral nucleus (thalamus). In contrast, for PPD < HC, we found clusters in: bilateral insula, precentral gyrus and MTG, ACC, putamen and inferior parietal lobule; right MFG, paracentral lobule and lingual gyrus; and left supramarginal, superior temporal, superior frontal, medial frontal, cingulate, inferior frontal and fusiform gyri and precuneus (Table S8; supplementary Figure S6; uncorrected P = 0.05, 500mm2). As some resulting clusters overlapped, we investigated the study contributors and found that, in the same regions (bilateral ACC and insula, right MFG, MTG and precentral gyrus, inferior parietal lobule, left medial frontal gyrus), structural correlates were increased but resting-state or task-based functional outcomes were decreased in PPD. However, in the remaining regions (left precuneus, superior frontal, cingulate and lingual gyri) mixed results were found, which may be explained by the different outcomes of each resting-state fMRI study (e.g., ReHo, fALFF).

**Figure S6. Results of the ALE meta-analyses on direction of effect (increased and decreased) in PPD patients (depicted in radiological convention).
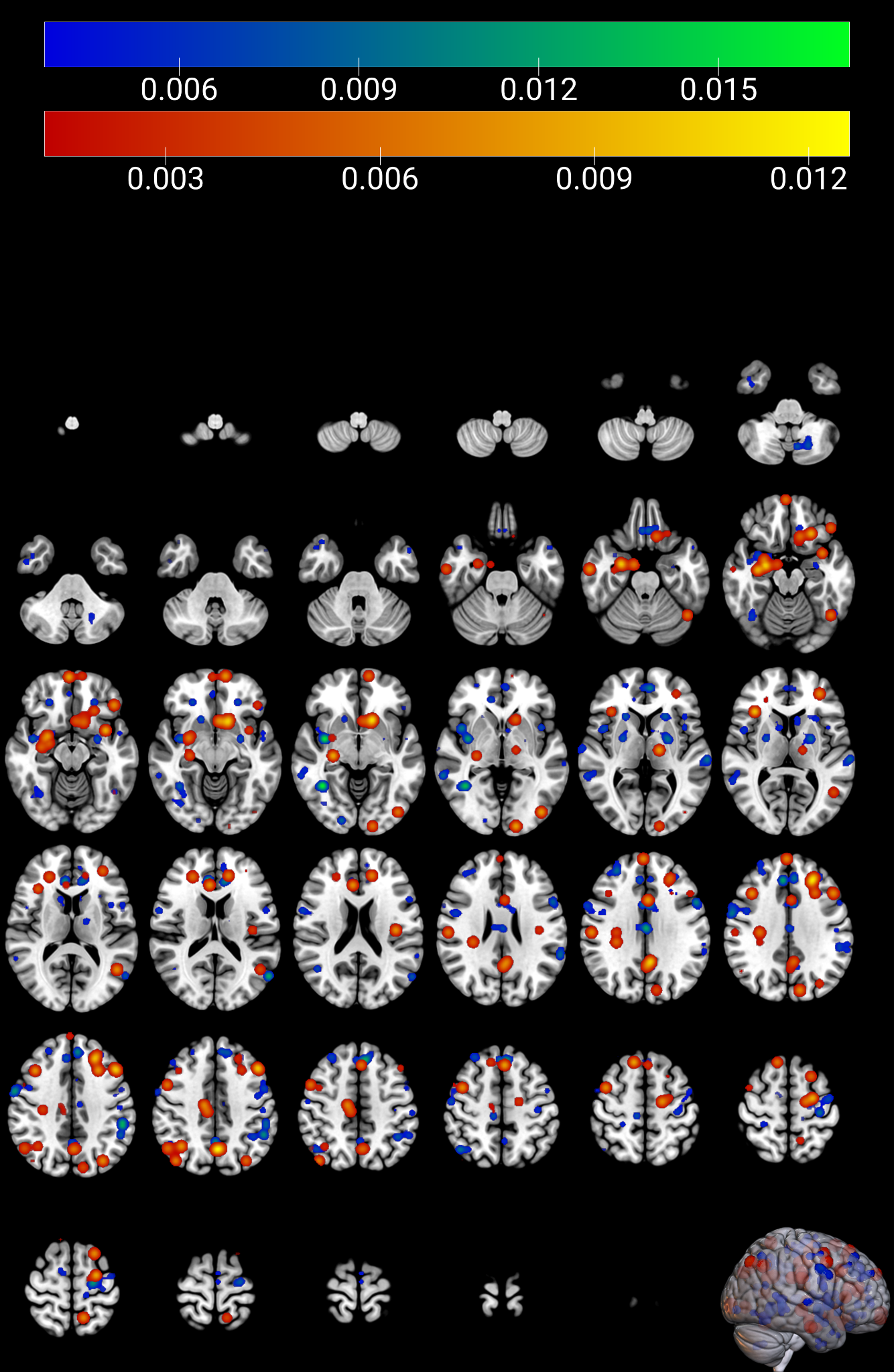
**

**Table S10. Results of the ALE meta-analyses on fMDD participants, in comparison to HC.**

| **Cluster** | **Volume (mm3)** | **Hem** | **Region** | **ALE** | **P** | **Z** | **MNI Coordinates** | | |
| --- | --- | --- | --- | --- | --- | --- | --- | --- | --- |
|  |  |  |  |  |  |  | **x** | **y** | **z** |
| **Structural** | | | | | | | | | |
| 1 | 1368 | R | extra-nuclear | 0.02 | <0.001 | 5.59 | 24 | 0 | -14 |
| 2 | 512 | L | extra-nuclear | 0.0118 | <0.001 | 4.08 | -28 | 10 | -10 |
|  |  |  | putamen | 0.0098 | <0.001 | 3.77 | -22 | 0 | -10 |
| **Task-based fMRI** | | | | | | | | | |
| 1 | 800 | L | anterior cingulate | 0.0129 | <0.001 | 4.14 | -6 | 40 | -8 |
| **Multimodal** | | | | | | | | | |
| 1 | 2664 | R | extra-nuclear | 0.0228 | <0.001 | 5.23 | 26 | 0 | -14 |
|  |  |  | uncus | 0.0123 | <0.001 | 3.56 | 26 | 6 | -28 |
| **Increased effect** | | | | | | | | | |
| 1 | 12344 | L | lateral globus pallidus | 0.0105 | <0.001 | 3.72 | -20 | -2 | -6 |
|  |  |  | extra-nuclear | 0.009 | <0.001 | 3.45 | -2 | -12 | 16 |
|  |  |  | ventral lateral nucleus | 0.0089 | <0.001 | 3.39 | -16 | -14 | 4 |
|  |  |  | medial globus pallidus | 0.0085 | <0.001 | 3.30 | -12 | 2 | -2 |
|  |  |  | pulvinar | 0.0085 | <0.001 | 3.29 | -18 | -28 | 8 |
|  |  |  | putamen | 0.0083 | <0.001 | 3.23 | -24 | 6 | 6 |
|  |  |  | ventral lateral nucleus | 0.0076 | <0.001 | 2.98 | -16 | -10 | 10 |
|  |  |  | lentiform nucleus | 0.0055 | <0.05 | 2.38 | -26 | -18 | 4 |
| 2 | 8512 | R | uncus | 0.0124 | <0.001 | 4.09 | 26 | 8 | -28 |
|  |  |  | lateral globus pallidus | 0.0097 | <0.001 | 3.57 | 20 | 2 | -2 |
|  |  |  | subcallosal gyrus | 0.0086 | <0.001 | 3.34 | 26 | 8 | -18 |
|  |  |  | inferior frontal gyrus | 0.0086 | <0.001 | 3.33 | 34 | 14 | -22 |
|  |  |  | putamen | 0.0078 | <0.001 | 3.04 | 30 | 6 | 2 |
| 3 | 5704 | R | medial frontal gyrus | 0.0106 | <0.001 | 3.75 | 8 | 12 | 46 |
|  |  |  | medial frontal gyrus | 0.0087 | <0.001 | 3.36 | 8 | 6 | 54 |
|  |  |  | sub-gyral | 0.0074 | <0.05 | 2.92 | 28 | 22 | 34 |
|  |  |  | cingulate gyrus | 0.0068 | <0.05 | 2.72 | 12 | 20 | 34 |
| 4 | 5296 | L | middle temporal gyrus | 0.0117 | <0.001 | 3.94 | -44 | -62 | 30 |
|  |  |  | middle temporal gyrus | 0.0083 | <0.001 | 3.23 | -44 | -74 | 20 |
|  |  |  | middle occipital gyrus | 0.0082 | <0.001 | 3.21 | -42 | -72 | 12 |
|  |  |  | inferior occipital gyrus | 0.005 | <0.05 | 2.28 | -44 | -74 | -4 |
| 5 | 4680 | L | anterior cingulate | 0.0111 | <0.001 | 3.83 | -10 | 48 | -8 |
|  |  |  | anterior cingulate | 0.0094 | <0.001 | 3.51 | -6 | 40 | -8 |
|  |  |  | superior frontal gyrus | 0.0084 | <0.001 | 3.29 | -18 | 58 | -8 |
| 6 | 4672 | L | cingulate gyrus | 0.0091 | <0.001 | 3.45 | -8 | 16 | 42 |
|  |  |  | cingulate gyrus | 0.0085 | <0.001 | 3.3 | -6 | 12 | 36 |
|  |  |  | cingulate gyrus | 0.0083 | <0.001 | 3.24 | -6 | 20 | 24 |
|  |  |  | cingulate gyrus | 0.0054 | <0.05 | 2.38 | -12 | -2 | 32 |
| 7 | 3440 | L | superior frontal gyrus | 0.0085 | <0.001 | 3.32 | -24 | 50 | 22 |
|  |  |  | middle frontal gyrus | 0.0075 | <0.05 | 2.95 | -38 | 50 | 8 |
|  |  |  | middle frontal gyrus | 0.007 | <0.05 | 2.8 | -36 | 48 | 24 |
| 8 | 3312 | R | lingual gyrus | 0.0086 | <0.001 | 3.45 | 12 | -72 | 2 |
|  |  |  | lingual gyrus | 0.0074 | <0.05 | 2.91 | 12 | -84 | -2 |
|  |  |  | lingual gyrus | 0.0071 | <0.05 | 2.8 | 0 | -80 | -2 |
| 9 | 3224 | L | anterior cingulate | 0.0071 | <0.05 | 2.8 | -2 | 24 | -16 |
|  |  | R | anterior cingulate | 0.0052 | <0.05 | 2.3 | 4 | 16 | -16 |
|  |  | R | extra-nuclear | 0.005 | <0.05 | 2.27 | 4 | 18 | -6 |
| 10 | 2416 | L | parahippocampal gyrus | 0.0093 | <0.001 | 3.49 | -24 | -30 | -14 |
|  |  |  | hippocampus | 0.0073 | <0.05 | 2.86 | -32 | -32 | -10 |
| 11 | 2344 | R | insula | 0.0085 | <0.001 | 3.32 | 42 | -16 | 6 |
|  |  |  | insula | 0.008 | <0.001 | 3.14 | 36 | -14 | 14 |
| 12 | 2336 | L | postcentral gyrus | 0.0086 | <0.001 | 3.35 | -42 | -20 | 32 |
|  |  |  | postcentral gyrus | 0.0072 | <0.05 | 2.86 | -50 | -20 | 24 |
| 13 | 2312 | L | superior frontal gyrus | 0.0083 | <0.001 | 3.22 | -22 | 28 | 42 |
|  |  |  | sub-gyral | 0.0076 | <0.001 | 2.98 | -20 | 26 | 30 |
| 14 | 2208 | L | inferior frontal gyrus | 0.0092 | <0.001 | 3.47 | -48 | 20 | -8 |
| 15 | 2200 | L | frontal lobe sub-gyral | 0.0105 | <0.001 | 3.72 | -31 | 10 | 40 |
| 16 | 2168 | R | postcentral gyrus | 0.0085 | <0.001 | 3.32 | 60 | -10 | 14 |
|  |  |  | insula | 0.0049 | <0.05 | 2.26 | 56 | -24 | 14 |
| 17 | 2112 | R | inferior parietal lobule | 0.0076 | <0.001 | 2.97 | 60 | -24 | 30 |
|  |  |  | precentral gyrus | 0.0071 | <0.05 | 2.82 | 62 | -16 | 36 |
| 18 | 2080 | L | substania graia | 0.0085 | <0.001 | 3.32 | -10 | -16 | -12 |
|  |  |  | substania graia | 0.005 | <0.05 | 2.28 | -2 | -28 | -10 |
| 19 | 1128 | L | frontal lobe sub-gyral | 0.0085 | <0.001 | 3.31 | -20 | -2 | 46 |
| 20 | 1112 | R | superior temporal gyrus | 0.0082 | <0.001 | 3.2 | 56 | -20 | -2 |
| 21 | 1112 | L | frontal lobe sub-gyral | 0.0083 | <0.001 | 3.24 | -14 | -16 | 64 |
| 22 | 1096 | L | medial frontal gyrus | 0.0067 | <0.05 | 2.7 | -4 | -6 | 68 |
| 23 | 1088 | L | cingulate gyrus | 0.0079 | <0.001 | 3.07 | -2 | -36 | 28 |
| 24 | 1088 | L | postcentral gyrus | 0.0071 | <0.05 | 2.82 | -60 | -20 | 32 |
| 25 | 1088 | L | middle frontal gyrus | 0.0066 | <0.05 | 2.65 | -50 | 20 | 32 |
| 26 | 1088 | R | frontal lobe sub-gyral | 0.0086 | <0.001 | 3.34 | 22 | 40 | 38 |
| 27 | 1088 | L | precentral gyrus | 0.0077 | <0.001 | 3 | -26 | -16 | 56 |
| 28 | 1080 | R | subthalamic nucleus | 0.0084 | <0.001 | 3.28 | 12 | -18 | -10 |
| 29 | 1080 | R | supramarginal gyrus | 0.0086 | <0.001 | 3.33 | 46 | -48 | 34 |
| 30 | 1072 | L | cingulate gyrus | 0.0081 | <0.001 | 3.17 | -10 | -52 | 30 |
| 31 | 1072 | R | paracentral lobule | 0.081 | <0.001 | 3.16 | 12 | -32 | 68 |
| 32 | 1064 | L | cuneus | 0.0084 | <0.001 | 3.29 | 0 | -90 | 16 |
| 33 | 1064 | R | inferior parietal lobule | 0.0084 | <0.001 | 3.27 | 40 | -58 | 46 |
| 34 | 1056 | R | middle frontal gyrus | 0.0075 | <0.05 | 2.96 | 50 | 18 | 26 |
| 35 | 1056 | R | precentral gyrus | 0.0084 | <0.001 | 3.29 | 38 | -12 | 50 |
| 36 | 1048 | L | caudate head | 0.0083 | <0.001 | 3.25 | -14 | 18 | -6 |
| 37 | 1040 | R | anterior cingulate | 0.0084 | <0.001 | 3.26 | 10 | 56 | -12 |
| 38 | 1040 | R | temporal lobe sub-gyral | 0.0084 | <0.001 | 3.26 | 48 | -44 | -2 |
| 39 | 1040 | R | precuneus | 0.0083 | <0.001 | 3.22 | 16 | -50 | 34 |
| 40 | 1032 | L | middle occipital gyrus | 0.0084 | <0.001 | 3.26 | -24 | -94 | 26 |
| 41 | 1024 | L | posterior lobe declive | 0.0083 | <0.001 | 3.24 | -18 | -74 | -14 |
| 42 | 960 | L | superior temporal gyrus | 0.0083 | <0.001 | 3.24 | -44 | 12 | -30 |
| 43 | 944 | R | culmen | 0.0083 | <0.001 | 3.24 | 12 | -48 | -20 |
| 44 | 944 | L | medial frontal gyrus | 0.0071 | <0.05 | 2.83 | -6 | 56 | 18 |
| 45 | 936 | L | superior temporal gyrus | 0.0054 | <0.05 | 2.37 | -56 | -22 | -2 |
| 46 | 904 | L | temporal lobe sub-gyral | 0.005 | <0.05 | 2.27 | -48 | -32 | -12 |
| 47 | 880 | R | frontal lobe sub-gyral | 0.0055 | <0.05 | 2.4 | 44 | 36 | -10 |
| 48 | 872 | R | parahippocampal gyrus | 0.049 | <0.05 | 2.25 | 32 | -20 | -26 |
| 49 | 856 | L | frontal lobe sub-gyral | 0.005 | <0.05 | 2.27 | -48 | 10 | 18 |
| 50 | 840 | R | extra-nuclear | 0.0049 | <0.05 | 2.26 | 14 | -36 | 4 |
| 51 | 816 | R | inferior frontal gyrus | 0.0071 | <0.05 | 2.83 | 58 | 24 | 2 |
| 52 | 816 | L | precuneus | 0.005 | <0.05 | 2.28 | -18 | -54 | 54 |
| 53 | 808 | R | frontal lobe sub-gyral | 0.005 | <0.05 | 2.28 | 22 | 30 | -22 |
| **Decreased effect** | | | | | | | | | |
| 1 | 16160 | R | extra-nuclear | 0.02 | <0.001 | 5.16 | 24 | 0 | -14 |
|  |  |  | medial dorsal nucleus | 0.0138 | <0.001 | 4.07 | 0 | -14 | 4 |
|  |  |  | insula | 0.0113 | <0.001 | 3.61 | 38 | 12 | -6 |
|  |  |  | claustrum | 0.0095 | <0.001 | 3.29 | 40 | 0 | 2 |
|  |  |  | extra-nuclear | 0.0092 | <0.001 | 3.19 | 10 | 2 | -16 |
|  |  |  | extra-nuclear | 0.0091 | <0.001 | 3.17 | 14 | 0 | 2 |
|  |  |  | ventral lateral nucleus | 0.0088 | <0.001 | 3.12 | 14 | -10 | 2 |
|  |  |  | extra-nuclear | 0.0086 | <0.001 | 3.03 | 34 | 0 | 2 |
|  |  |  | thalamus | 0.0081 | <0.05 | 2.89 | 14 | -6 | 16 |
|  |  |  | insula | 0.0079 | <0.05 | 2.86 | 44 | -6 | 6 |
| 2 | 12208 | R | corpus callosum | 0.0132 | <0.001 | 3.95 | 16 | 34 | 8 |
|  |  |  | extra-nuclear | 0.0114 | <0.001 | 3.62 | 20 | 18 | 10 |
|  |  |  | anterior cingulate | 0.0097 | <0.001 | 3.33 | 18 | 42 | 14 |
|  |  |  | medial frontal gyrus | 0.009 | <0.001 | 3.16 | 14 | 46 | 28 |
|  |  |  | sub-gyral | 0.0087 | <0.001 | 3.07 | 24 | 10 | 22 |
|  |  |  | medial frontal gyrus | 0.0071 | <0.05 | 2.64 | 16 | 52 | 18 |
|  |  |  | sub-gyral | 0.0069 | <0.05 | 2.59 | 36 | 16 | 22 |
|  |  |  | middle frontal gyrus | 0.0063 | <0.05 | 2.42 | 36 | 32 | 32 |
| 3 | 7472 | L | extra-nuclear | 0.0118 | <0.001 | 3.71 | -28 | 10 | -10 |
|  |  |  | putamen | 0.0098 | <0.001 | 3.35 | -22 | 0 | -10 |
|  |  |  | extra-nuclear | 0.0098 | <0.001 | 3.34 | -30 | 6 | -4 |
|  |  |  | insula | 0.0083 | <0.05 | 2.97 | -44 | -8 | -2 |
|  |  |  | extra-nuclear | 0.0079 | <0.05 | 2.84 | -30 | 6 | 6 |
|  |  |  | insula | 0.0074 | <0.05 | 2.7 | -40 | 0 | 6 |
| 4 | 7096 | L | brainstem | 0.0121 | <0.001 | 3.74 | -12 | -14 | -18 |
|  |  | R | brainstem | 0.0097 | <0.001 | 3.31 | 12 | -14 | -18 |
|  |  | R | parahippocampal gyrus | 0.0091 | <0.001 | 3.19 | 18 | -18 | -22 |
|  |  | L | brainstem | 0.0087 | <0.001 | 3.07 | 0 | -18 | -16 |
|  |  | R | hippocampus | 0.0083 | <0.05 | 2.97 | 22 | -22 | -10 |
|  |  | L | parahippocampal gyrus | 0.0081 | <0.05 | 2.91 | -26 | -14 | -22 |
| 5 | 6576 | L | superior frontal gyrus | 0.0184 | <0.001 | 4.91 | -24 | 50 | 20 |
|  |  |  | lateral ventricle | 0.0093 | <0.001 | 3.25 | -14 | 30 | 4 |
|  |  |  | corpus callosum | 0.0091 | <0.001 | 3.19 | -16 | 36 | 8 |
| 6 | 5816 | L | extra-nuclear | 0.0165 | <0.001 | 4.58 | -10 | -4 | 16 |
|  |  |  | lateral ventricle | 0.009 | <0.001 | 3.18 | -2 | 4 | 4 |
|  |  |  | - | 0.0087 | <0.001 | 3.67 | 2 | 8 | -4 |
|  |  |  | putamen | 0.0087 | <0.001 | 3.06 | -14 | 8 | 2 |
| 7 | 3968 | L | anterior cingulate | 0.0111 | <0.001 | 3.57 | 2 | 36 | 0 |
|  |  |  | anterior cingulate | 0.0098 | <0.001 | 3.34 | -6 | 46 | 0 |
| 8 | 3000 | L | parahippocampal gyrus | 0.0096 | <0.001 | 3.30 | -16 | -42 | -8 |
|  |  |  | parahippocampal gyrus | 0.009 | <0.001 | 3.16 | -26 | -52 | -4 |
|  |  |  | culmen | 0.0085 | <0.001 | 3.01 | -12 | -50 | -8 |
| 9 | 2400 | R | frontal lobe sub-gyral | 0.0076 | <0.05 | 2.74 | 30 | 32 | -6 |
|  |  |  | frontal lobe sub-gyral | 0.0055 | <0.05 | 2.25 | 44 | 36 | -10 |
|  |  |  | extra-nuclear | 0.0051 | <0.05 | 2.17 | 20 | 30 | -8 |
| 10 | 1928 | R | medial frontal gyrus | 0.0097 | <0.001 | 3.32 | 20 | -6 | 52 |
|  |  |  | sub-gyral | 0.0079 | <0.05 | 2.85 | 20 | 4 | 56 |
| 11 | 1920 | L | precuneus | 0.0076 | <0.05 | 2.73 | -2 | -66 | 60 |
|  |  |  | precuneus | 0.0072 | <0.05 | 2.65 | -6 | -60 | 48 |
| 12 | 1832 | L | frontal lobe sub-gyral | 0.0097 | <0.001 | 3.32 | -38 | 40 | 2 |
|  |  |  | frontal lobe sub-gyral | 0.0068 | <0.05 | 2.56 | -28 | 36 | -2 |
| 13 | 1832 | L | middle frontal gyrus | 0.0095 | <0.001 | 3.28 | -24 | 2 | 66 |
|  |  |  | middle frontal gyrus | 0.0093 | <0.001 | 3.23 | -18 | -8 | -62 |
| 14 | 1800 | R | temporal lobe sub-gyral | 0.0088 | <0.001 | 3.12 | 40 | -32 | 8 |
|  |  |  | insula | 0.0072 | <0.05 | 2.65 | 46 | -28 | 18 |
| 15 | 1784 | L | temporal lobe sub-gyral | 0.0079 | <0.05 | 2.84 | -50 | -46 | -4 |
|  |  |  | temporal lobe sub-gyral | 0.005 | <0.05 | 2.15 | -44 | -34 | -2 |
| 16 | 1728 | R | postcentral gyrus | 0.0084 | <0.05 | 2.98 | 42 | -32 | 52 |
|  |  |  | postcentral gyrus | 0.0076 | <0.05 | 2.79 | 42 | -26 | 54 |
| 17 | 1712 | R | superior temporal gyrus | 0.0072 | <0.05 | 2.66 | 54 | -52 | 29 |
|  |  |  | supramarginal gyrus | 0.0051 | <0.05 | 2.16 | 62 | -54 | 26 |
| 18 | 1680 | R | fusiform gyrus | 0.007 | <0.05 | 2.6 | 30 | -64 | -8 |
|  |  |  | lingual gyrus | 0.0055 | <0.05 | 2.24 | 36 | -74 | -10 |
| 19 | 1064 | L | calcarine | 0.0088 | <0.001 | 3.11 | 2 | -98 | 2 |
| 20 | 936 | L | posterior cingulate | 0.005 | <0.05 | 2.13 | -8 | -64 | 20 |
|  |  |  | posterior cingulate | 0.0049 | <0.05 | 2.11 | -2 | -64 | 20 |
| 21 | 904 | L | superior frontal gyrus | 0.0091 | <0.05 | 2.9 | -12 | 58 | -10 |
| 22 | 896 | - | - | 0.0086 | <0.001 | 3.02 | -19 | -69 | -54 |
| 23 | 896 | R | cerebellar tonsil | 0.0093 | <0.001 | 3.21 | 29 | -42 | -47 |
| 24 | 896 | R | cerebellar tonsil | 0.0086 | <0.001 | 3.02 | 17 | -63 | -40 |
| 25 | 896 | L | extra-nuclear | 0.0087 | <0.001 | 3.07 | -10 | 0 | -16 |
| 26 | 896 | L | medial frontal gyrus | 0.0083 | <0.05 | 2.95 | -4 | 8 | 64 |
| 27 | 880 | R | culmen | 0.0078 | <0.05 | 2.82 | 24 | -40 | -16 |
| 28 | 880 | R | lingual gyrus | 0.0095 | <0.001 | 3.27 | 21 | -90 | 6 |
| 29 | 856 | R | medial frontal gyrus | 0.0084 | <0.05 | 2.97 | 4 | 60 | 8 |
| 30 | 824 | R | medial frontal gyrus | 0.0076 | <0.05 | 2.72 | 14 | -18 | 58 |
| 31 | 808 | L | culmen | 0.0067 | <0.05 | 2.54 | -6 | -58 | 2 |
| 32 | 800 | R | inferior frontal gyrus | 0.0076 | <0.05 | 2.72 | 34 | 30 | -22 |
| 33 | 776 | R | inferior parietal lobule | 0.0067 | <0.05 | 2.54 | 60 | -32 | 26 |
| 34 | 768 | L | fusiform gyrus | 0.0067 | <0.05 | 2.54 | -36 | -38 | -20 |
| 35 | 768 | R | superior temporal gyrus | 0.0075 | <0.05 | 2.72 | 56 | -14 | -8 |
| 36 | 768 | L | sub-gyral | 0.0069 | <0.05 | 2.59 | -30 | -92 | 2 |
| 37 | 768 | R | extra-nuclear | 0.0065 | <0.005 | 2.46 | 20 | -48 | 21 |
| 38 | 768 | R | cingulate gyrus | 0.0075 | <0.05 | 2.72 | 8 | 28 | 40 |
| 39 | 768 | R | precuneus | 0.0067 | <0.05 | 2.54 | 8 | -54 | 52 |
| 40 | 752 | R | lingual gyrus | 0.0068 | <0.05 | 2.59 | 8 | -64 | 4 |
| 41 | 744 | L | lingual gyrus | 0.0091 | <0.001 | 3.17 | -4 | -84 | 0 |
| 42 | 744 | L | angular gyrus | 0.008 | <0.05 | 2.89 | -50 | -60 | 36 |
| 43 | 744 | R | paracentral lobule | 0.0071 | <0.05 | 2.65 | 4 | -12 | 46 |
| 44 | 720 | L | medial frontal gyrus | 0.0069 | <0.05 | 2.59 | -12 | 34 | -26 |
| 45 | 704 | R | inferior frontal gyrus | 0.0073 | <0.05 | 2.67 | 30 | 20 | -30 |
| 46 | 704 | L | posterior cingulate | 0.0084 | <0.05 | 2.99 | 0 | -42 | 22 |
| 47 | 656 | L | superior temporal gyrus | 0.0054 | <0.05 | 2.23 | -56 | -22 | -2 |
| 48 | 648 | L | cingulate gyrus | 0.0055 | <0.05 | 2.23 | -12 | -2 | 32 |
| 49 | 640 | L | lentiform nucleus | 0.0053 | <0.05 | 2.21 | -26 | -18 | 4 |
| 50 | 584 | L | posterior lobe declive | 0.005 | <0.05 | 2.15 | -24 | -84 | -18 |
| 51 | 552 | R | posterior lobe declive | 0.0049 | <0.05 | 2.15 | 6 | -80 | -20 |
| 52 | 552 | L | postcentral gyrus | 0.005 | <0.05 | 2.13 | -40 | -26 | 50 |

For each cluster volume, hemisphere, region, ALE value, peak p, z and MNI coordinates are provided.

**Table S11 and Figure S7. Direction of effect analyses in fMDD**

Eighteen experiments reported greater activity/connectivity or brain structure in fMDD than in HC (293 participants) and 24 experiments reported less (521 participants). For fMDD > HC, we observed significant clusters in: bilateral putamen, ACC and inferior frontal, postcentral and superior temporal gyri; right insula, uncus, lateral globus pallidus, subthalamic nucleus, inferior parietal lobule, precuneus, culmen and subcallosal, medial frontal, lingual, supramarginal and precentral gyri; left globus pallidus, ventral lateral nucleus, pulvinar, cuneus, caudate head and MTG, middle occipital, superior frontal, cingulate and parahippocampal gyri. For fMDD < HC, clusters were found in: bilateral ACC and corpus callosum; right medial dorsal nucleus, insula, ventral lateral nucleus, medial frontal and parahippocampal gyri; left putamen, MFG, superior frontal, parahippocampal and lingual gyri (Table S11; supplementary Figure S7). Regions overlapping in both analyses (bilateral ACC, right insula, right medial frontal and superior frontal gyri, left putamen) show increased resting-state or task-based activity/connectivity, alongside reduced structural correlates.

**Figure S7. Results of the ALE meta-analyses on direction of effect (increased and decreased) in fMDD patients (depicted in radiological convention).**


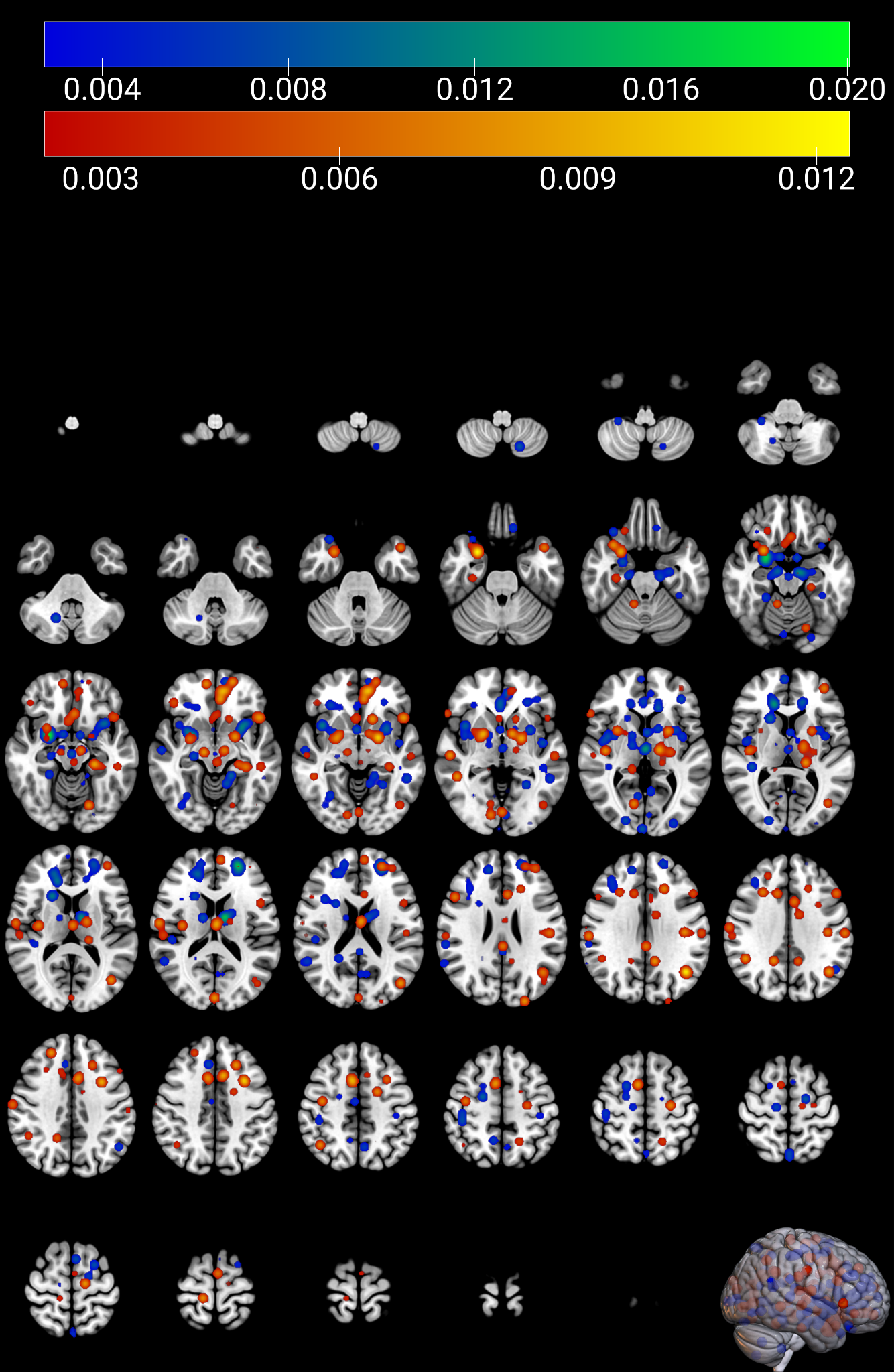


**Table S12. Results of the conjunction aALE meta-analysis between PPD and fMDD.**

| **Cluster** | **Volume (mm3)** | **Hem** | **Region** | **ALE** | **P** | **Z** | **MNI Coordinates** | | |
| --- | --- | --- | --- | --- | --- | --- | --- | --- | --- |
|  |  |  |  |  |  |  | **x** | **y** | **z** |
| **Conjunction** | | | | | | | | | |
| 1 | 4096 | R | extra-nuclear | 0.0197 | - | - | 26 | -2 | -14 |
|  |  |  | insula | 0.0082 | - | - | 40 | 10 | -2 |
|  |  |  | putamen | 0.0069 | - | - | 34 | 0 | -2 |
|  |  |  | extra-nuclear | 0.0067 | - | - | 38 | -2 | 0 |
|  |  |  | extra-nuclear | 0.0065 | - | - | 38 | 2 | -2 |
| 2 | 1016 | L | ventral lateral nucleus | 0.0088 | - | - | -16 | -14 | 4 |
|  |  |  | ventral lateral nucleus | 0.006 |  |  | -16 | -8 | 12 |
| 3 | 560 | R | putamen | 0.0072 | - | - | 24 | -2 | 8 |
| 4 | 528 | L | sub-gyral | 0.0076 | - | - | -18 | -12 | 60 |
|  |  |  | sub-gyral | 0.0065 | - | - | -18 | -16 | 64 |
| 5 | 488 | L | caudate | 0.0084 | - | - | -14 | 18 | -6 |
| 6 | 480 | L | extra-nuclear | 0.0075 | - | - | -30 | 10 | -12 |
|  |  |  | extra-nuclear | 0.0072 |  |  | -34 | 10 | -14 |
| 7 | 376 | L | cingulate gyrus | 0.0085 | - | - | -6 | 12 | 36 |
| 8 | 248 | L | extra-nuclear | 0.0084 | - | - | -26 | -2 | -12 |
| 9 | 216 | L | putamen | 0.0065 | - | - | -24 | 2 | 6 |

For each cluster volume, hemisphere, region, ALE value, peak p, z and MNI coordinates are provided.

**Figure S8. Results of the ALE meta-analysis with an age-matched subgroup of fMDD studies (displayed in radiological convention)**


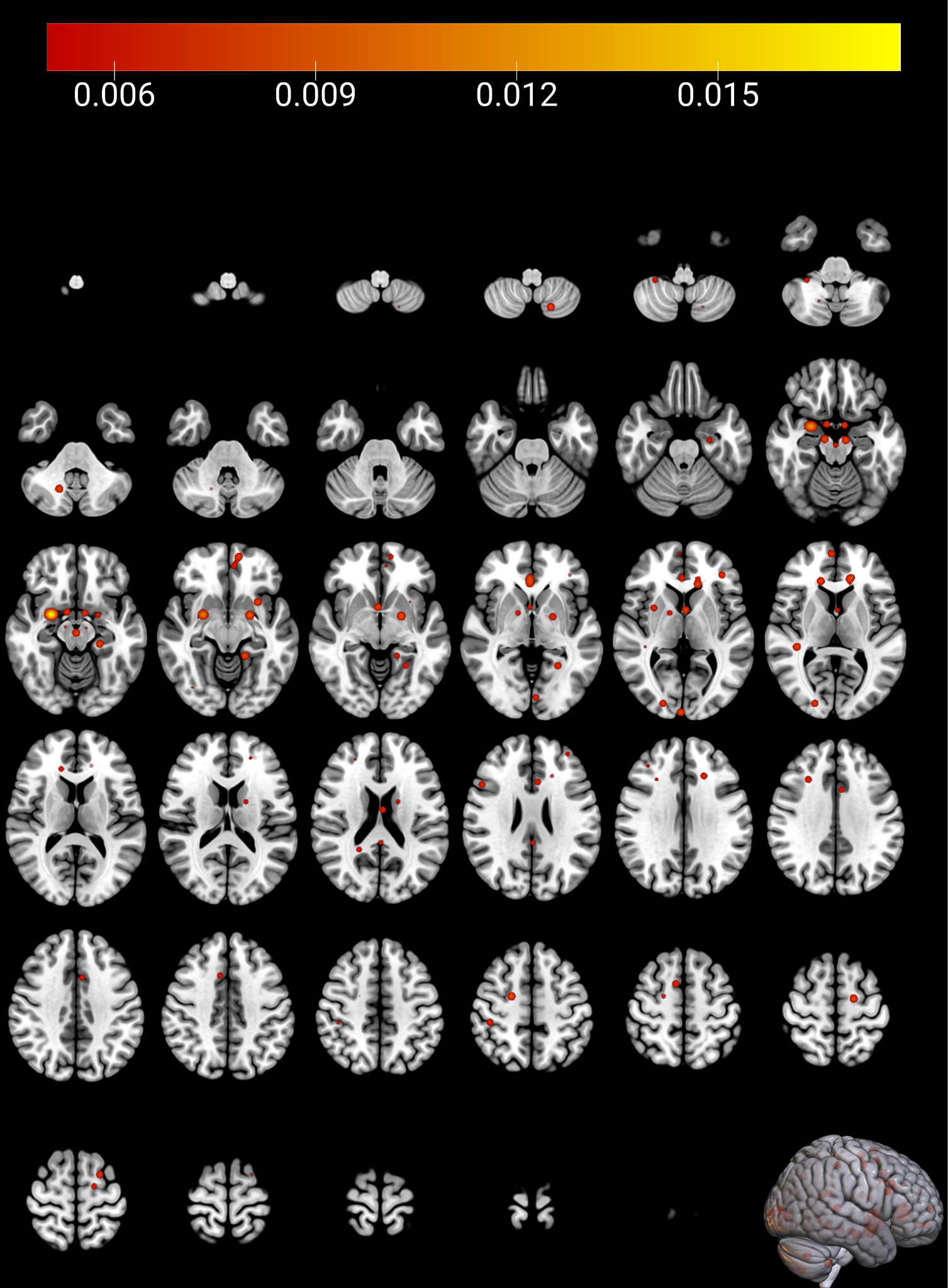


**Simulated dataset for specificity analysis**

//Reference=MNI

//Study 1, Random Study, fMRI task Reference=MNI

//Subjects=32

34 -54 18

28 -60 24

36 -50 16

//Study 2, Random Study, fMRI task Reference=MNI

//Subjects=15

-28 20 -8

-30 18 -12

//Study 3, Random Study, fMRI task Reference=MNI

//Subjects=42

12 6 40

//Study 4, Random Study, fMRI task Reference=MNI

//Subjects=20

-46 -68 24

-50 -70 18

//Study 5, Random Study, fMRI task Reference=MNI

//Subjects=38

52 -12 16

//Study 6, Random Study, fMRI task Reference=MNI

//Subjects=12

-36 50 -20

-40 52 -18

-32 48 -22

//Study 7, Random Study, fMRI task Reference=MNI

//Subjects=27

24 -86 12

22 -84 10

26 -88 16

//Study 8, Random Study, fMRI task Reference=MNI

//Subjects=45

-8 32 -4

-12 30 -2

-4 34 -6

-10 28 -8

//Study 9, Random Study, fMRI task Reference=MNI

//Subjects=18

18 -18 56

//Study 10, Random Study, fMRI task Reference=MNI

//Subjects=36

-50 -22 8

-48 -20 12

//Study 11, Random Study, fMRI task Reference=MNI

//Subjects=23

4 -40 -8

6 -38 -6

//Study 12, Random Study, fMRI task Reference=MNI

//Subjects=14

30 -4 28

32 -6 30

//Study 13, Random Study, fMRI task Reference=MNI

//Subjects=50

-60 12 36

-62 14 38

-58 10 34

//Study 14, Random Study, fMRI task Reference=MNI

//Subjects=19

6 24 -16

4 22 -14

//Study 15, Random Study, fMRI task Reference=MNI

//Subjects=28

-34 -6 50

-36 -4 52

-32 -8 48

//Study 16, Random Study, fMRI task Reference=MNI

//Subjects=30

28 54 8

30 56 6

26 52 10

//Study 17, Random Study, fMRI task Reference=MNI

//Subjects=22

-48 14 -12

//Study 18, Random Study, fMRI task Reference=MNI

//Subjects=16

20 -76 18

18 -74 20

//Study 19, Random Study, fMRI task Reference=MNI

//Subjects=40

-56 42 -6

-58 44 -8

-54 40 -4

//Study 20, Random Study, fMRI task Reference=MNI

//Subjects=25

8 -26 36

//Study 21, Random Study, fMRI task Reference=MNI

//Subjects=34

40 -12 -20

42 -14 -22

//Study 22, Random Study, fMRI task Reference=MNI

//Subjects=11

-12 40 24

//Study 23, Random Study, fMRI task Reference=MNI

//Subjects=37

16 -44 28

18 -46 26

14 -42 30

//Study 24, Random Study, fMRI task Reference=MNI

//Subjects=29

-22 30 -14

-24 28 -12

//Study 25, Random Study, fMRI task Reference=MNI

//Subjects=21

10 -58 40

12 -60 38

8 -56 42
